# Discovery of a Peripherally Directed CB_1_ Receptor Antagonist That Restores Insulin Signaling in Adipocytes

**DOI:** 10.1021/acsomega.6c07565

**Published:** 2026-10-13

**Authors:** Jhonny Azuaje, Rubén Prieto-Díaz, Claudia Gioé-Gallo, Sandra Ortigueira, Jacobo Soilán, Diego Triñanes, Hugo Fojo, María I. Loza, Marialessandra Contino, Maria Grazia Perrone, Giuseppe Felice Mangiatordi, Pietro Delre, Nerea Lago-Baameiro, Adriana Estévez-López, Maria Pardo, Angela Stefanachi, Giovanni Graziano, José Brea, Maria Majellaro, Eddy Sotelo

**Affiliations:** †Centro Singular de Investigación en Química Biolóxica e Materiais Moleculares (CiQUS), ‡Departamento de Química Orgánica, Facultade de Farmacia, 430234 Universidade de Santiago de Compostela, 15782 Santiago de Compostela, Spain; § Centro Singular de Investigación en Medicina Molecular e Enfermidades Crónicas (CIMUS), Universidade de Santiago de Compostela, 15782 Santiago de Compostela, Spain; ∥ Dipartimento di Farmacia-Scienze del Farmaco, Università degli Studi di Bari ALDO MORO, via Orabona 4, 70125 Bari, Italy; ⊥ CNR—Institute of Crystallography, Via Giovanni Amendola, 122/O, 70126 Bari, Italy; #Grupo Obesidómica, Área de Endocrinología,Nutrición y Metabolismo. Instituto de Investigación Sanitaria de Santiago (IDIS).Complexo Hospitalario Universitario de Santiago de Compostela, ∇CIBER de Fisiopatología de la Obesidad y la Nutrición, Instituto de Salud Carlos III, Santiago de Compostela 15706, Spain; ○ Celtarys Research, Avenida de Barcelona, 7, Santiago de Compostela 15706, Spain

## Abstract

The antagonism of cannabinoid 1 receptor (CB_1_R) signaling has demonstrated clinical efficacy in treating obesity, improving insulin resistance, enhancing cardiovascular health, and mitigating metabolic risk factors in humans. Despite these positive outcomes, the clinical use of rimonabant was limited by severe central nervous system adverse effects, which reduced interest in CB_1_R and hindered the exploration of new therapeutic opportunities. Selective targeting of peripheral CB1R represents a promising strategy to overcome these limitations, creating therapeutic opportunities not only for obesity and metabolic disorders but also for smoking and alcohol addiction. Herein, we report a library of 53 novel, potent, and subtype-selective CB_1_R antagonists bearing polar groups, designed through convergent Ugi-based synthetic pathways. This approach enabled the identification of key structure–activity relationship features within this series and the effective modulation of molecular properties governing blood–brain barrier permeability. Among the compounds evaluated, the piperazine-1-ium derivative **24a** stands out as the first permanently charged CB_1_R antagonist, exhibiting nanomolar affinity (*K*_*i*_ = 115 nM) and high CB_1_R/CB_2_R selectivity with a favorable physicochemical profile consistent with peripheral restriction and limited blood-brain barrier penetration. Importantly, **24a** exhibits robust functional activity in a biologically relevant model of metabolic dysfunction. In insulin-resistant, lipid-hypertrophied adipocytes, **24a** restores insulin-stimulated Akt phosphorylation and significantly enhances lipolytic activity, with effects comparable or superior to those of rimonabant at equivalent concentrations. These findings establish **24a** as a promising peripherally restricted CB_1_R antagonist with demonstrated functional efficacy in a metabolic disease-relevant cellular model.

## Graphical abstract

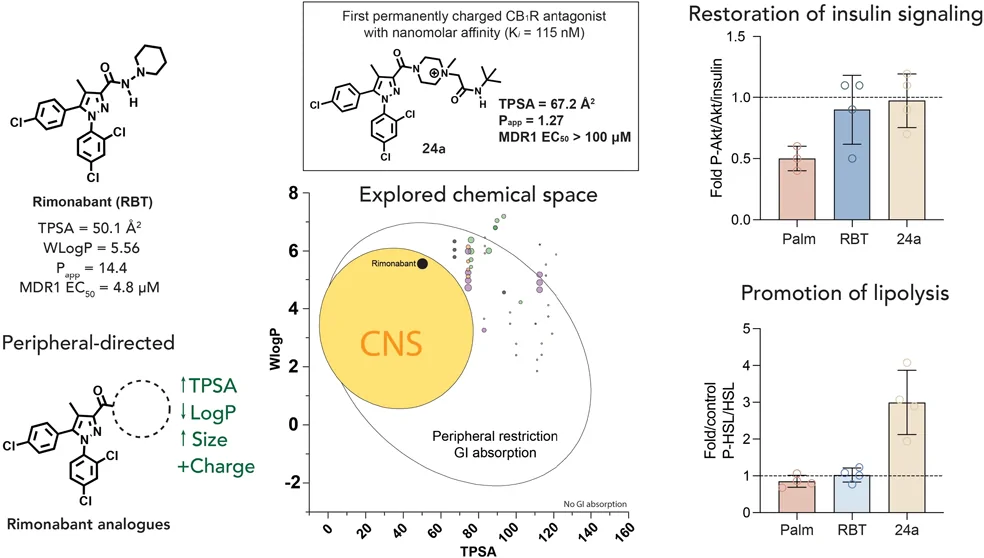

## Introduction

The endocannabinoid system (ECS) comprises cannabinoid receptors (CBRs), transporters, endogenous ligands (endocannabinoids), and enzymes involved in endocannabinoid biosynthesis, transport, and degradation.^1,2^ It plays a central role in appetite regulation, energy balance, and metabolic homeostasis, including insulin signaling in adipose tissue and liver.^3^ ECS modulation encompasses innovative therapeutic approaches to address unmet medical needs^4^ arising from serious diseases,^2,5,6^ such as inflammation, obesity, and diabetes.^7–10^ Two distinct cannabinoid receptors have been identified, CB_1_R and CB_2_R, both belonging to the rhodopsin-like class A G-protein-coupled receptors.^11^ These receptors mediate both the physiological effects of endocannabinoids and most of the pharmacological effects of the bioactive components of *Cannabis sativa*.^12–14^

CB_2_R is primarily located on blood and immune cells,^15,16^ where it modulates inflammatory responses. In contrast, CB_1_R is expressed widely in both central and peripheral nerve terminals,^17–19^ as well as in metabolically relevant peripheral tissues.^20,21^ Moreover, CB_1_R activation in peripheral tissues plays a significant role in energy balance and metabolic processes.^22–25^ Importantly, CB_1_R overactivation in adipose tissue and liver has also been associated with impaired Akt phosphorylation, disrupted insulin signaling and insulin resistance in the context of obesity and metabolic disorders,^26,27^ underscoring the therapeutic potential of peripheral CB_1_R antagonism beyond appetite suppression. Growing evidence confirms that blocking CB_1_R in peripheral tissues can suppress food intake, increase energy expenditure, and reduce lipogenesis.^25,28,29^ Recent data have also highlighted the beneficial effects of rimonabant and other CB_1_R antagonists on hepatic steatosis.^30^

Endocannabinoids acting through hypothalamic CB_1_R exert a well-established appetite-promoting role,^31^ and ECS overactivation prompted the development of CB_1_R antagonists for treating obesity and its metabolic complications.^10,32^ These efforts culminated in the development of several clinical candidates (Figure 1, Cpds **1–5**).^33–36^ Rimonabant, approved in 2006 for obesity, effectively reduced body weight and cardiometabolic complications.^37^ However, Rimonabant central CB_1_R action led to severe neuropsychiatric side effects, including anxiety, depression, and suicidal ideation, which eventually led to its withdrawal.^28,38–40^ These side effects also halted the clinical development of other structurally unrelated CB_1_R antagonists (Figure 1, Cpds **2–5**).^41^

**Figure 1:**
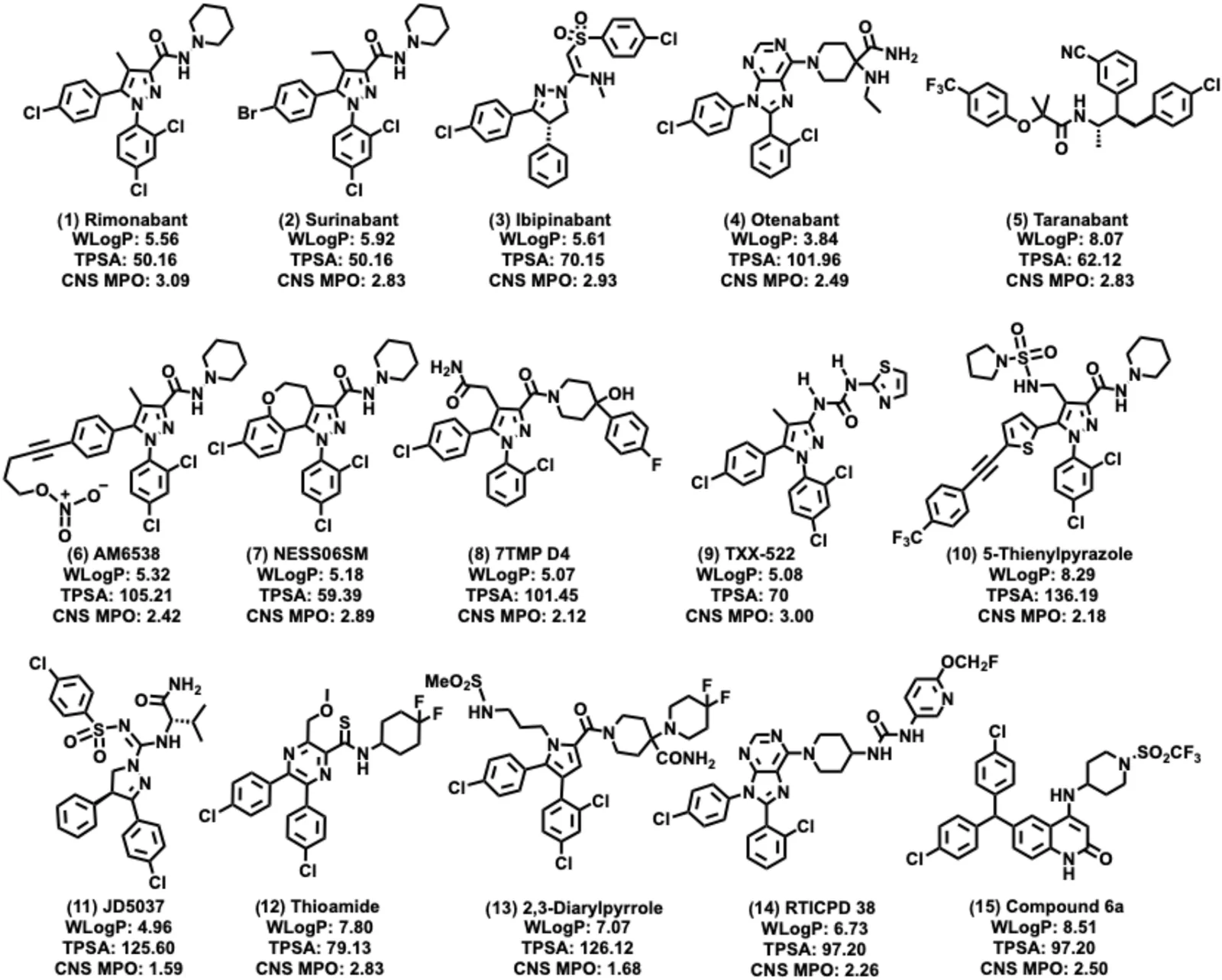
CB_1_R ligands that entered in clinical studies (**1–5**) and representative peripheral selective CB_1_R ligands.^12,33–36,42–50^ Molecular descriptors [TPSA (Topological Polar Surface Area) and W log *P*] and CNS MPO were calculated using RDKit and the Instant JChem software (Chemaxon) respectively.

The recognition of the clinical value of peripheral CB_1_R modulation for controlling obesity and its metabolic comorbidities has led to the development of novel generations of CB_1_R antagonists (Figure 1, Cpds **6–15**).^12,33–36,42–50^ The primary design criterion for these families is peripheral restriction—limiting brain penetration to avoid CNS activity—achieved by exploiting the physiological properties of the blood-brain barrier (BBB).^51,52^ Three effective structural modifications within the development of peripherally restricted drugs include:^52^ (1) introducing polar functional groups to reduce Log *P* and increase TPSA values, (2) incorporating permanently charged moieties that reduce the drug’s ability to passively cross the BBB, and (3) introducing structural features that make the ligand a substrate for efflux proteins present at the BBB level such as the P-glycoprotein (P-gp). Multiparameter optimization algorithms such as CNS MPO^53^ integrate various physicochemical parameters to predict BBB permeation, with scores above 4 indicating penetration and below 3 indicating restriction.^53^ Examples include **TXX522** ^44^ and **JD5037** ^46^ (Figure 1), two candidates that retain metabolic efficacy with limited CNS exposure, exemplifying the peripheral restriction strategy pursued in the present work.

Beyond their standalone therapeutic utility, peripherally restricted CB_1_R antagonists have recently emerged as promising combination partners for glucagon-like peptide-1 receptor (GLP-1R) agonists. Preclinical evidence indicates that simultaneous CB_1_R and GLP-1R modulation yields additive or synergistic reductions in food intake and fat mass through complementary mechanisms of energy balance regulation.^54^ The clinical relevance of this combination has been validated by the CBeyond Phase 2a trial, in which nimacimab, a peripherally restricted CB_1_R inhibitory antibody, combined with semaglutide produced significantly greater weight loss than semaglutide alone, with a favorable safety profile and no neuropsychiatric adverse events.^55^

Drawing inspiration from rimonabant and adopting a pharmacokinetic selectivity approach, we report here a focused library of novel pyrazole derivatives bearing polar substituents assembled through a convergent Ugi-based synthetic strategy enabling time- and cost-effective structural diversification. This approach accelerated SAR analysis and modulation of BBB-permeability-related properties. Several ligands in this series combine high CB_1_R affinity, outstanding selectivity, and physicochemical profiles consistent with peripheral restriction. Notably, the piperazine-1-ium derivative **24a** stands out as the first permanently charged CB_1_R antagonist, combining nanomolar affinity (*K*_*i*_ = 115 nM), complete CB_1_R/CB_2_R selectivity, and a physicochemical profile that is consistent with limited BBB penetration. Importantly, **24a** restores insulin-stimulated Akt phosphorylation and promotes lipolysis in insulin-resistant, lipid-hypertrophied adipocytes, with effects exceeding those of rimonabant at equivalent concentrations.

## Results and Discussion

### Design and Synthesis

The design of the novel CB_1_R antagonists presented here was inspired by the proposed binding mode of rimonabant (**1**) with CB_1_R.^56^ Systematic studies on **1**, its analogues, and the first-generation CB_1_R antagonists/inverse agonists (Figure 1, compounds **1–10**) contributed to the establishment of the current pharmacophoric model for CB_1_R antagonists and inverse agonists (Figure 2). Intensive investigation into rimonabant derivatives explored the impact of structural diversification in both halogenated phenyl rings (arms 1 and 2 in Figure 2), bioisosteric replacements (including rigidification) in the central heterocyclic core, and the introduction of groups at position 4. These efforts resulted in the development of valuable molecular probes and ligands with improved profiles (Figure 1).^12,33–36,42–50^ Exploration of position 3 of pyrazole ring (arm 3) has recently yielded promising results, leading to the discovery of attractive ligands. The availability of crystal structures of CB_1_R, along with docking studies, has confirmed these findings, demonstrating that the group in arm 3 (R^3^) can access a spacious binding pocket capable of accommodating both polar and nonpolar groups (see Figure 2).^56,57^

**Figure 2:**
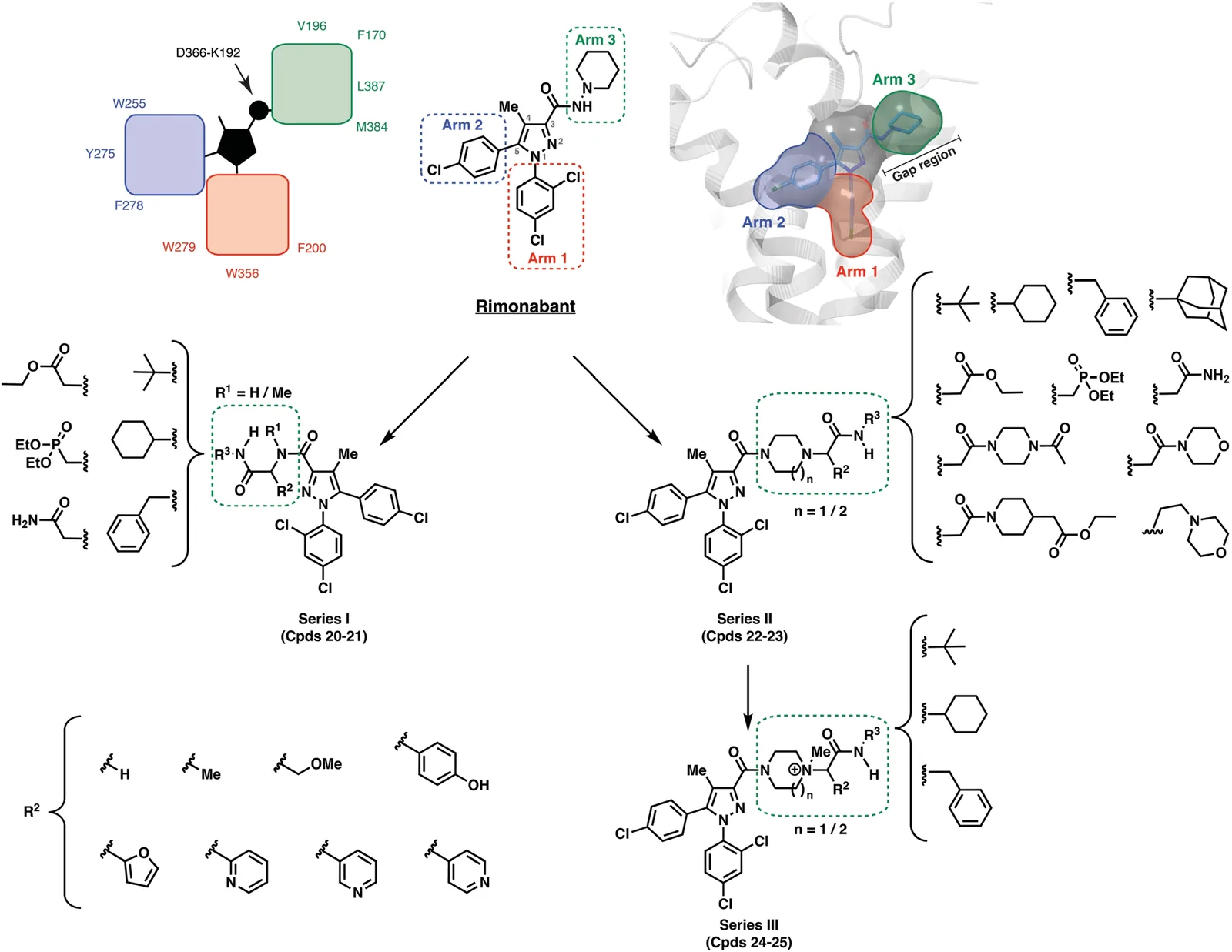
Structure of rimonabant (center), the 3 arms (green, red, and purple) pharmacophoric model of CB_1_R ligands (left), proposed binding modes of CB_1_R ligands (right) and general structure of the three series of herein described CB_1_R ligands.

For this project, we decided to keep the central scaffold, the methyl group at position 4, and both chloro-substituted phenyl rings at positions 1 and 5, which are involved in key aromatic stacking interactions. Our primary focus was on structurally diversifying arm 3 in the general pharmacophore model (highlighted by green squares in Figure 2). Given that the substituent at arm 3 (position 3 of the scaffold) is oriented toward the membrane, our design strategy had a dual purpose: to identify groups able to preserve favorable binding interactions while optimizing the physicochemical properties of the resulting ligands for a lower likelihood of BBB penetration.

The novel series were designed by decorating position 3 of the pyrazole core (Figure 2) with two different amide residues [acyclic (Series **I**) and heterocyclic (Series **II** and **III**)]. The proposed amide frameworks were conceived to occupy the gap region exposed to the membrane (Figure 2). The carboxamide carbonyl connecting the pyrazole core to arm 3 is proposed to anchor the scaffold within the binding site via an interaction with the D366-K192 salt bridge. The final ligands differ in topology, binding modes, and physicochemical features, enabling the capture of diverse conformational states within the CB_1_R and the optimization of pharmacokinetic parameters to obtain peripheral selective ligands while retaining attractive CB_1_R affinity and selectivity profiles. A distinctive feature of the design strategy is that both amide series (Figure 2) can be assembled in an efficient, convergent, and highly exploratory fashion from the carboxylic acid **16** through prototypic versions of the Ugi reaction (Scheme 1).^58^

**Scheme 1:**
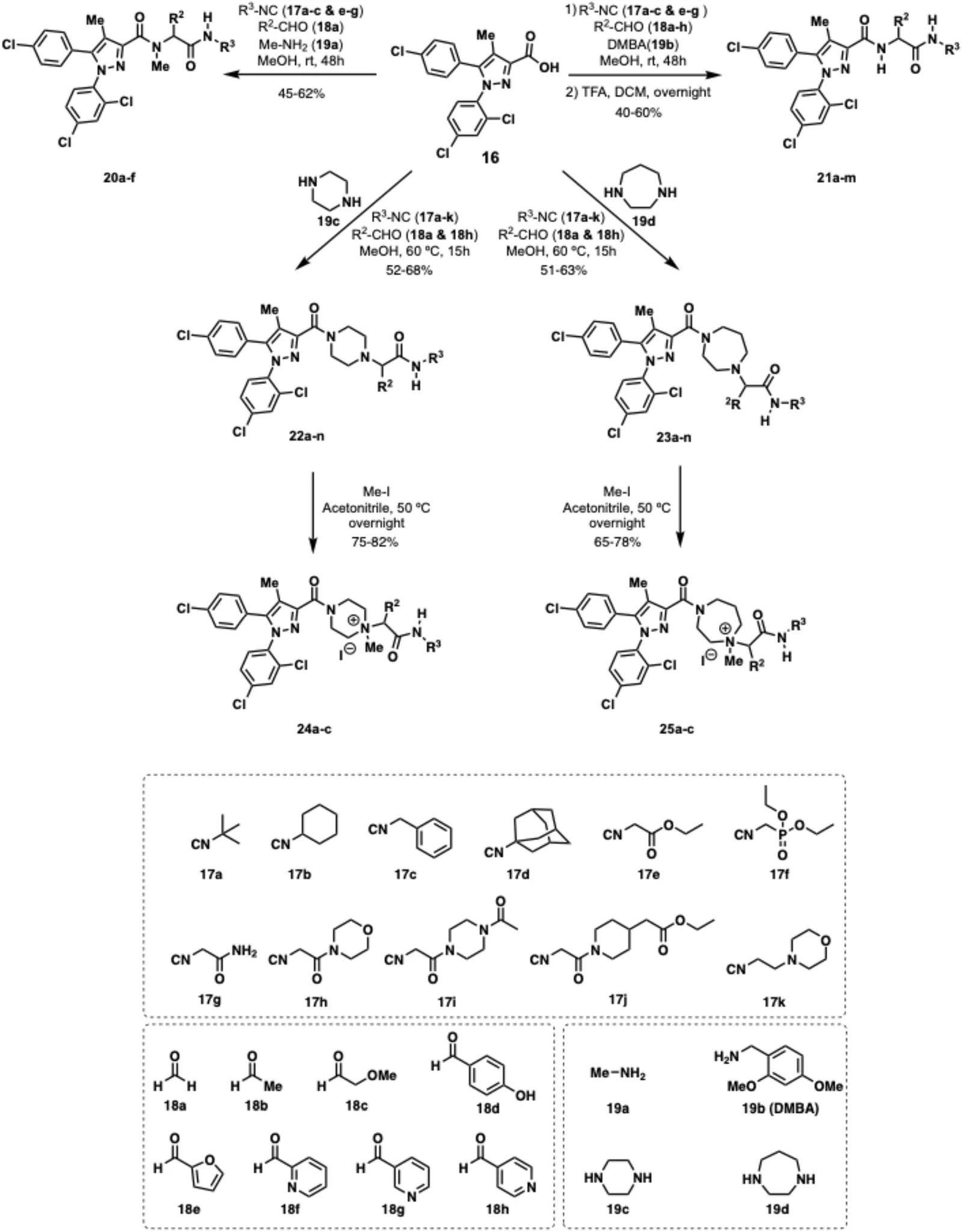
Synthetic Pathway for the Preparation of the Different Series of Pyrazole Derivatives Described Herein, Together with the Diversity Elements Employed

### Chemistry

A focused library, comprising 53 pyrazole derivatives (Scheme 1, Cpds **20–25**), was assembled using carboxylic acid **16** as the key precursor (Scheme 1). A characteristic feature of the envisioned synthetic strategy (Scheme 1) is that it enables the one-pot transformation of key precursor (**16**) into two structurally distinct frameworks (Figure 2, Series **I–III**). It also allows the time and cost-effective decoration through the simultaneous introduction of diversity elements offered by the precursors of the Ugi reaction (e.g., amines, aldehydes, and isocyanides). This facilitates the concurrent and effective optimization of binding modes and physicochemical properties.

The first amide subsets (Scheme 1, Cpds **20** and **21**) were obtained by following the general conditions of the highly reliable Ugi four-component reaction (U-4CR).^58,59^ Two amide subsets (R^1^ = Me or H) were prepared in moderate to satisfactory yields (see the Experimental Section) by reacting **16** with the required amine (**19a** or **19b**), isocyanides (**17**), and formaldehyde (**18a**). The isocyanide subset (**17a**–**f**) was chosen to investigate the influence of diverse structural features of R^3^ on ligand binding and physicochemical properties. The most promising ligand identified within Series I (**21b**) served as a template for exploring the effects of introducing polar groups at R^2^ (ligands **21f**–**l**). This exploration involved using a small collection of aldehydes (**18b**–**h**), which encompassed alkyl, aryl, and heteroaryl groups.

For the synthesis of the heterocyclic amides (Series **II** and **III**, Cpds **22–25**), we employed the split-Ugi reaction,^60,61^ an isocyanide-based multicomponent reaction resembling the U-4CR. However, instead of using a primary amine, this transformation utilizes a symmetric secondary diamine (as depicted in Scheme 1). This approach enabled us to efficiently and selectively desymmetrize the diamine core, yielding targeted ligands with satisfactory to excellent yields. Given the promising CB_1_R binding data obtained from the evaluation of representative members within this subset (Table 2, Cpds **22a**–**22c**), we expanded the series by incorporating 11 isocyanides (Scheme 1). The selected isocyanides, as outlined in Tables 2 and 3, provide notably higher TPSA and lower lipophilicity. This selection was made with the expectation that these isocyanides would offer a wide range of structural and physicochemical parameters, facilitating a detailed exploration of the influence of R^3^. As in the earlier series, we briefly investigated the impact of introducing a group at R^2^ in three representative ligands using three pyridyl carbaldehydes. Additionally, we took advantage of the nucleophilic properties of one of the nitrogen atoms in subsets **22** and **23**. This allowed us to transform three derivatives into their ammonium salts (**24** and **25**) through treatment with methyl iodide under mild conditions, as depicted in Scheme 1. A comprehensive description of the synthetic methods, structural, spectroscopic, and analytical data for all compounds is available in the Experimental Section and Supporting Information.

### Biological Evaluation

The affinities of the 53 pyrazole derivatives at the two human cannabinoid receptor subtypes (*h*CBRs), CB_1_R and CB_2_R, were assessed *in vitro* using radioligand binding assays, and the results are provided in Tables 1,2,3, and 4. The study employed *h*CBs expressed in transfected HEK cells. [^3^H]­CP55940 served as the radiotracer during the binding assays. As detailed in the tables and the Supporting Information, all compounds bearing a stereogenic center were evaluated as racemic mixtures.

**Table 1:**
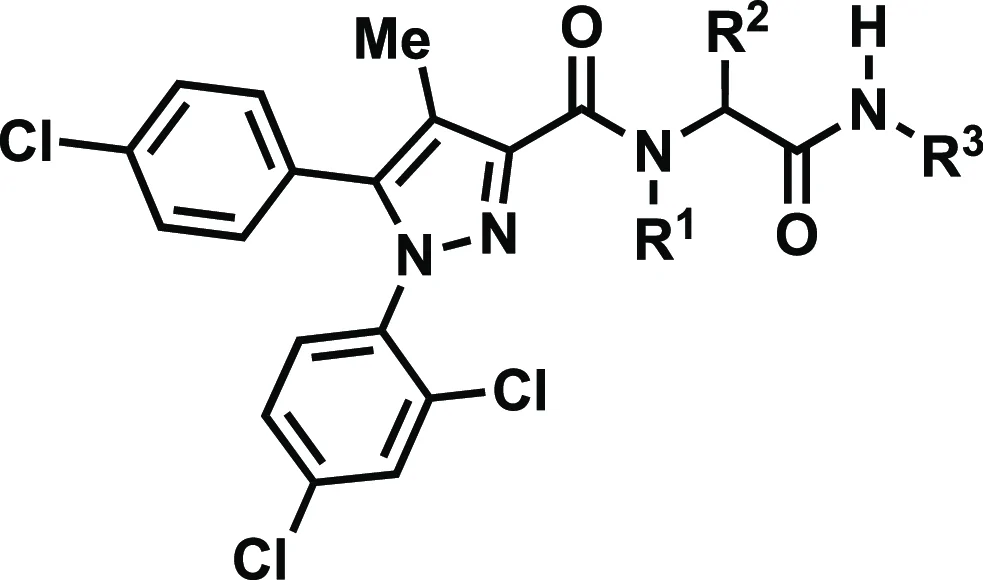

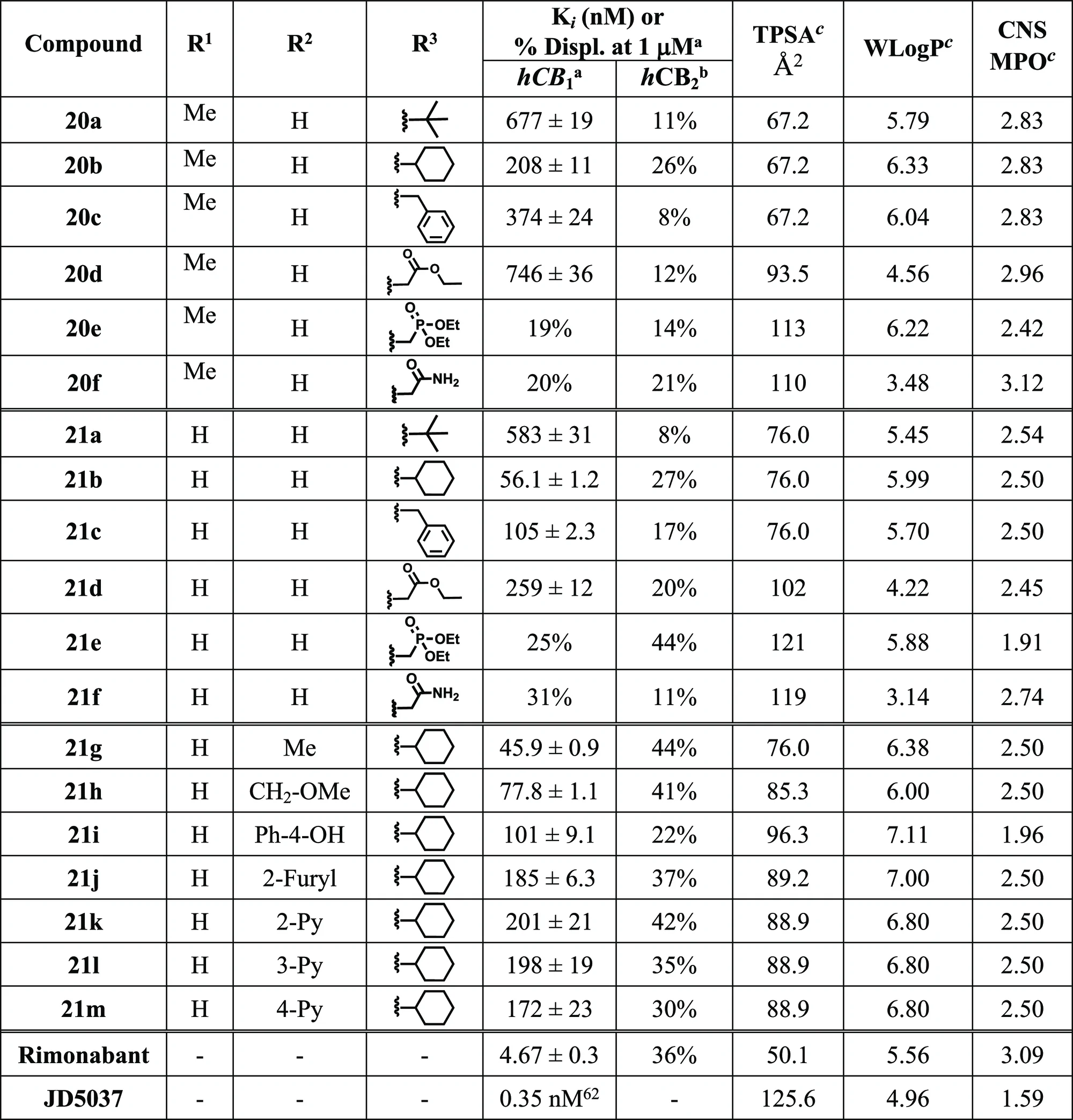
Structure, Binding Data at Human Cannabinoid Receptors, and Physicochemical Descriptors of Pyrazole Derivatives **20a–f** and **21a–m**

a Displacement of specific [^3^H]­CP55940 binding in human HEK cells expressed as *K*_*i*_ in nM ± SEM (*n* = 3) or percentage displacement of specific binding at a concentration of 1 μM (*n* = 2).

b Displacement of specific [^3^H]­CP55940 binding in human HEK cells expressed as *K*_*i*_ in nM ± SEM (*n* = 3) or percentage displacement of specific binding at a concentration of 1 μM (*n* = 2).

c Molecular descriptors (TPSA and W log *P*) and CNS MPO were calculated using RDKit and the Instant JChem software (Chemaxon) respectively.

**Table 2:**
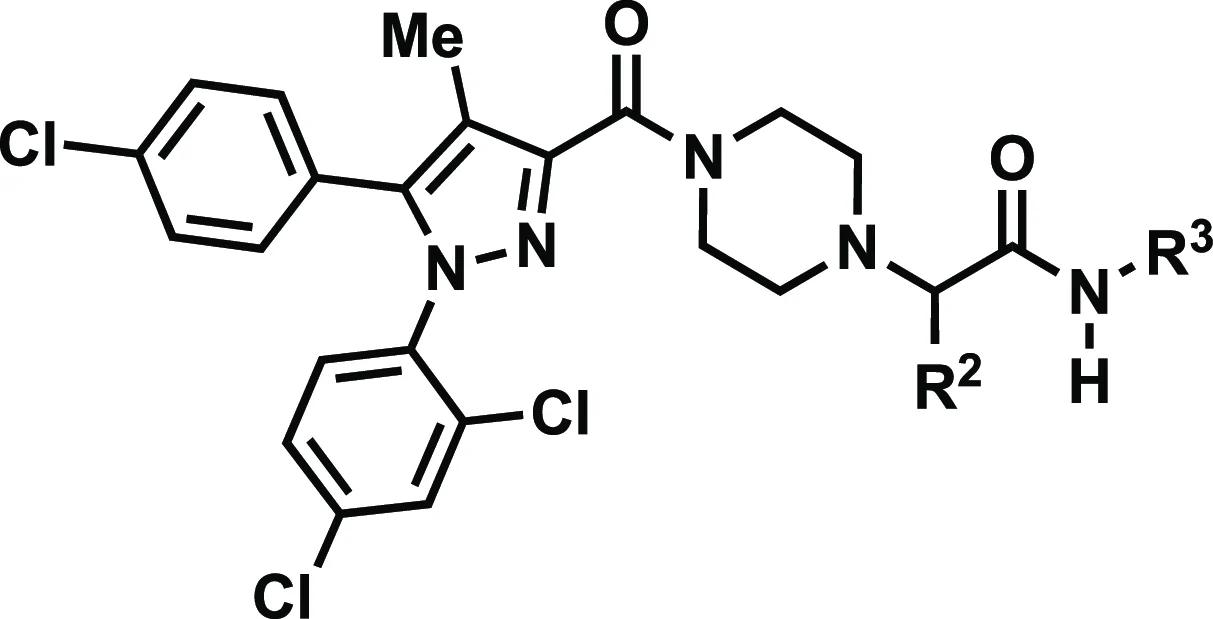

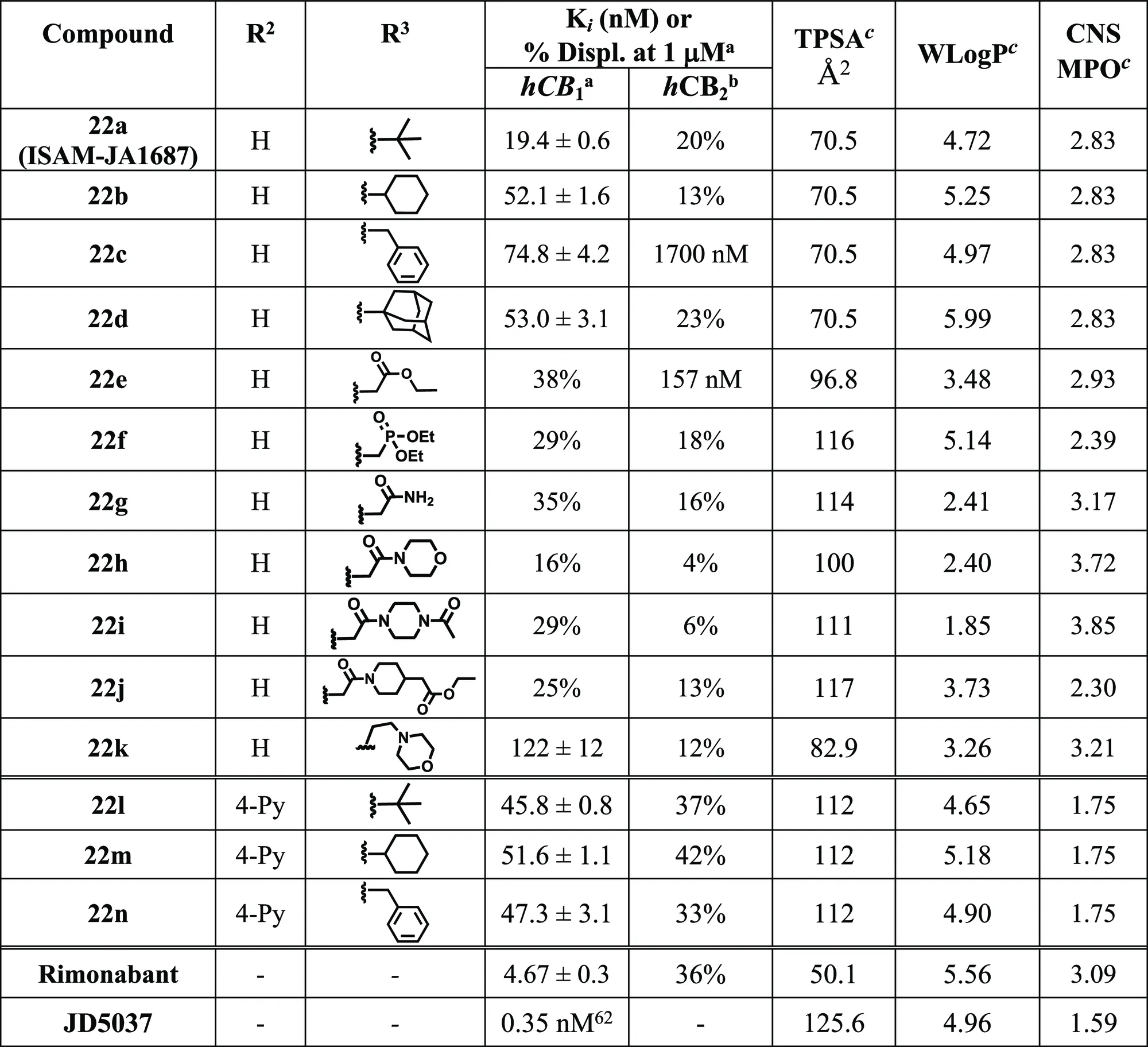
Structure, Binding Data at Human Cannabinoid Receptors, and Physicochemical Descriptors of Pyrazole Derivatives **23a**–**n**

a Displacement of specific [^3^H]­CP55940 binding in human HEK cells expressed as *K*_*i*_ in nM ± SEM (*n* = 3) or percentage displacement of specific binding at a concentration of 1 μM (*n* = 2).

b Displacement of specific [^3^H]­CP55940 binding in human HEK cells expressed as *K*_*i*_ in nM ± SEM (*n* = 3) or percentage displacement of specific binding at a concentration of 1 μM (*n* = 2).

c Molecular descriptors (TPSA and W log *P*) and CNS MPO were calculated using RDKit and the Instant JChem software (Chemaxon) respectively.

**Table 3:**
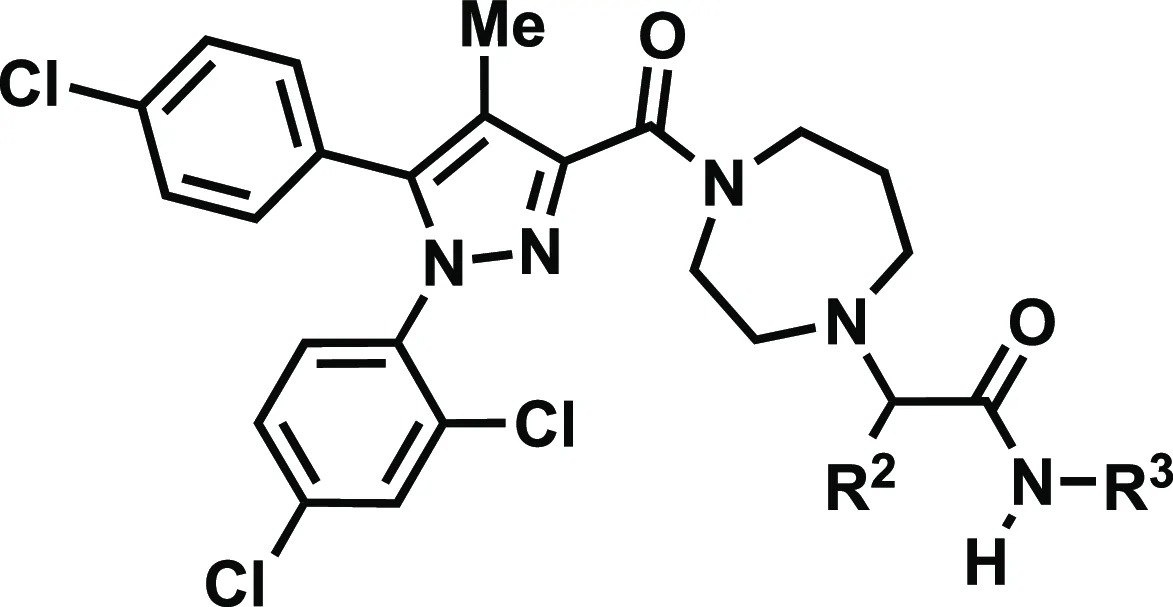

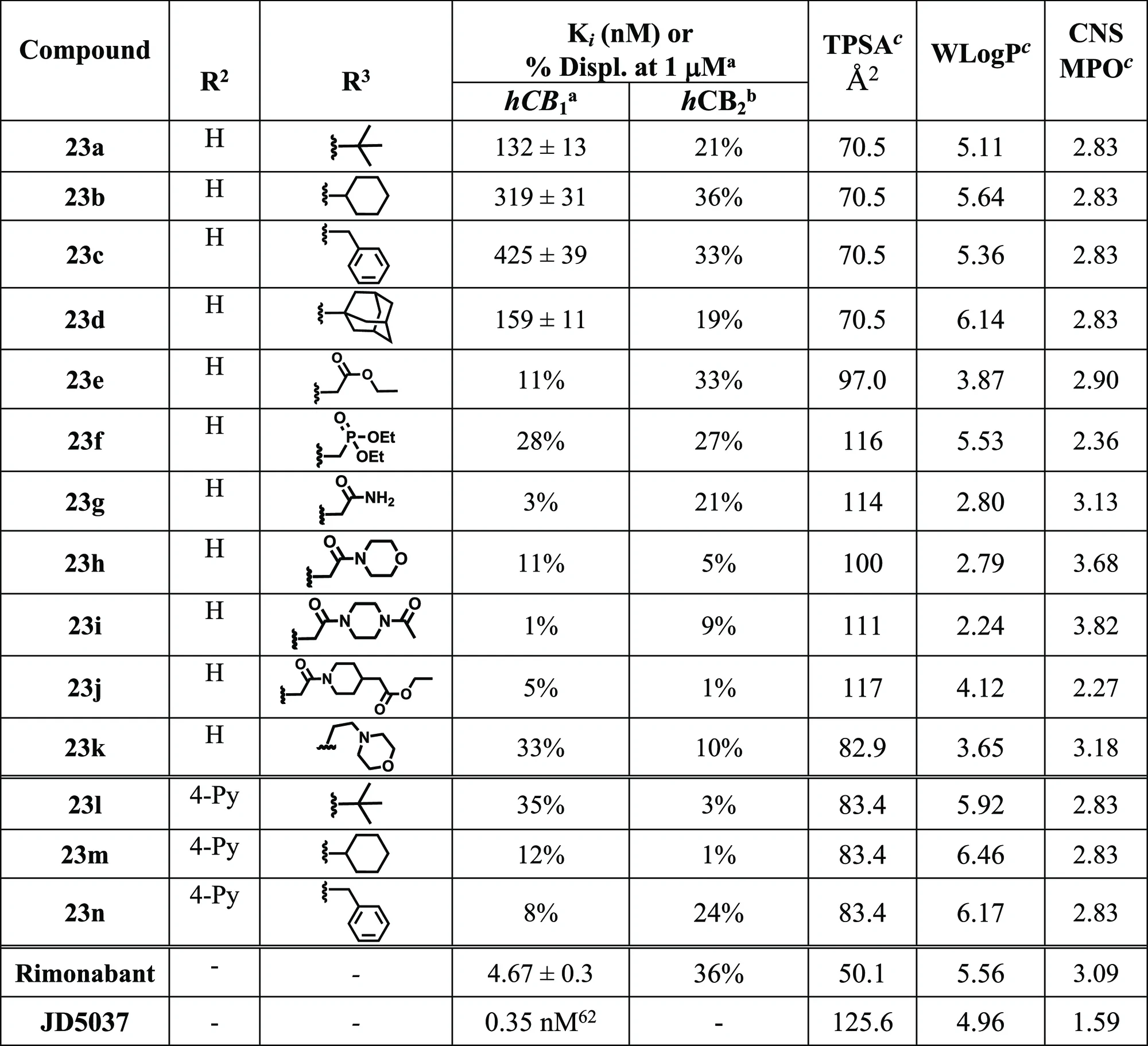
Structure, Binding Data at Human Cannabinoid Receptors, and Physicochemical Descriptors of Pyrazole Derivatives **23a**–**n**

a Displacement of specific [^3^H]­CP55940 binding in human HEK cells expressed as *K*_*i*_ in nM ± SEM (*n* = 3) or percentage displacement of specific binding at a concentration of 1 μM (*n* = 2).

b Displacement of specific [^3^H]­CP55940 binding in human HEK cells expressed as K_*i*_ in nM ± SEM (*n* = 3) or percentage displacement of specific binding at a concentration of 1 μM (*n* = 2).

c Molecular descriptors (TPSA and W log *P*) and CNS MPO were calculated using RDKit and the Instant JChem software (Chemaxon) respectively.

**Table 4:**
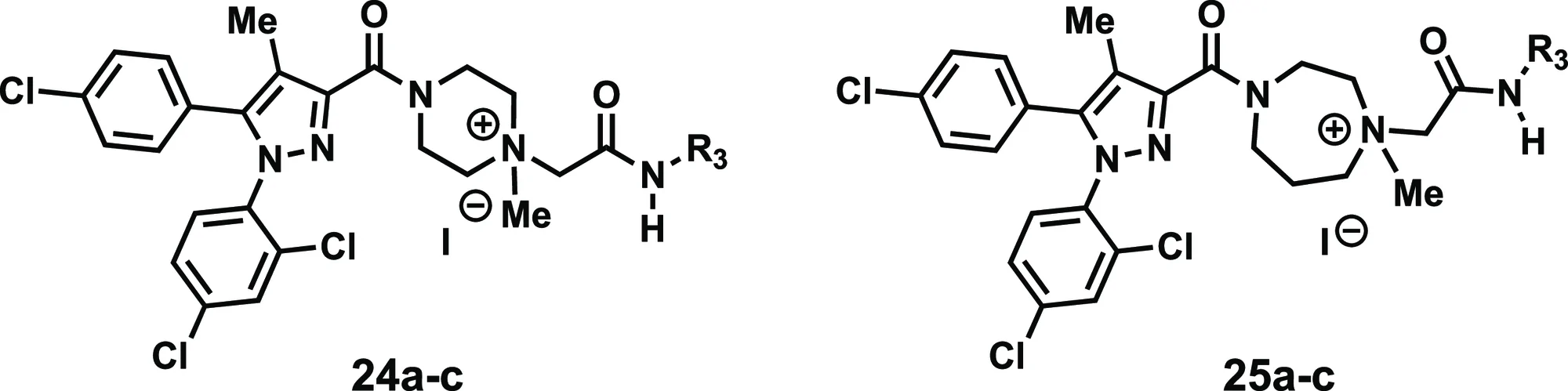

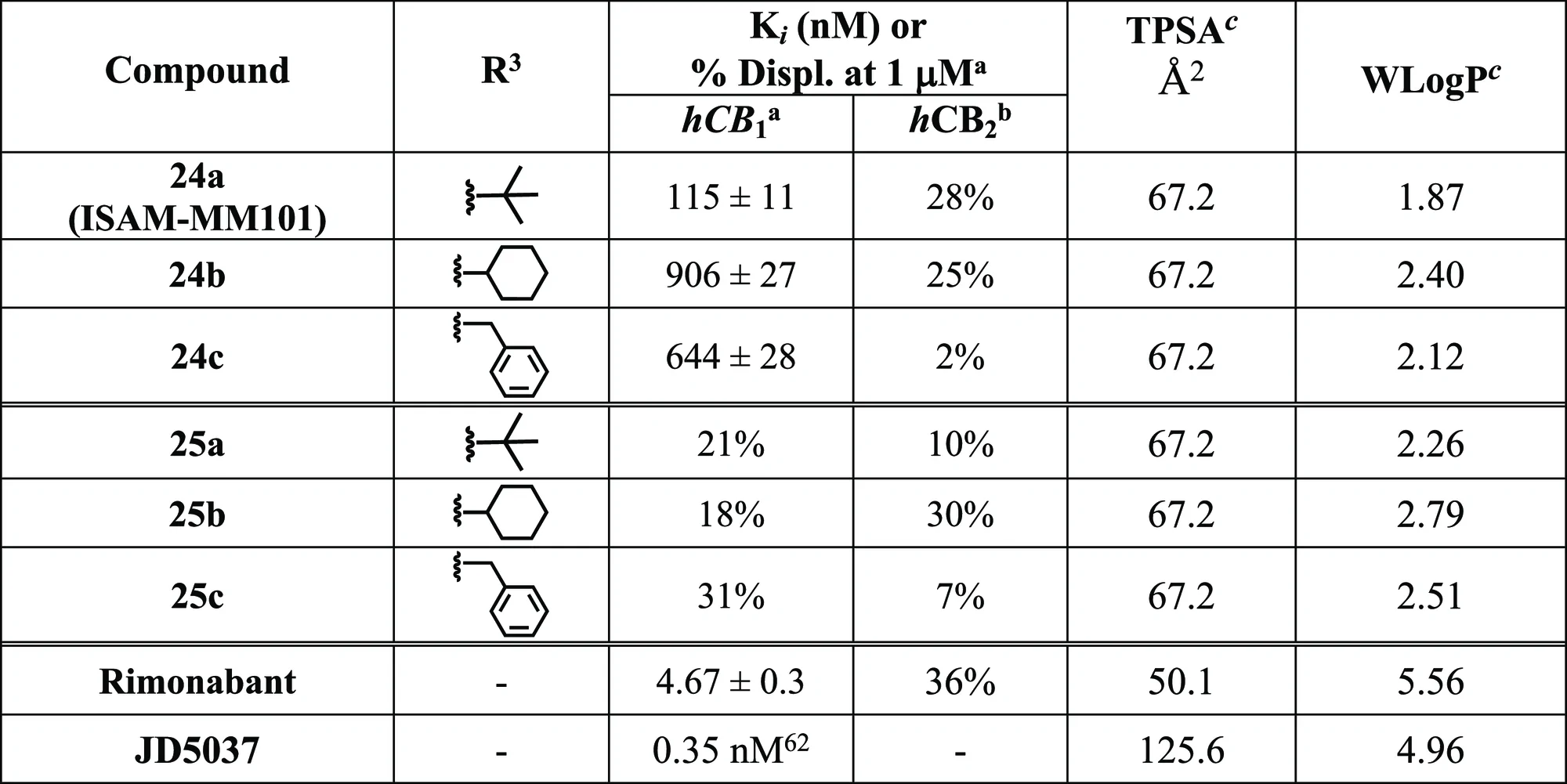
Structure, Binding Data at Human Cannabinoid Receptors, and Physicochemical Descriptors of Pyrazole Derivatives **24a**–**c** and **25a**–**c**

a Displacement of specific [^3^H]­CP55940 binding in human HEK cells expressed as *K*_*i*_ in nM ± SEM (*n* = 3) or percentage displacement of specific binding at a concentration of 1 μM (*n* = 2).

b Displacement of specific [^3^H]­CP55940 binding in human HEK cells expressed as *K*_*i*_ in nM ± SEM (*n* = 3) or percen-tage displacement of specific binding at a concentration of 1 μM (*n* = 2).

c Molecular descriptors (TPSA and W log *P*) were calculated using RDKit.

### Functional Experiments

Two representative ligands of the series, **22a** and **24a**, were subjected to additional assays to evaluate their functional behavior. For this purpose, the amount of cAMP produced by human cannabinoid receptor activity (see Experimental Section) was determined. Both compounds, together with Rimonabant (reference ligand) exhibited K_B_ values (24.7, 77.0, and 1.8 nM respectively) in the low nanomolar range, thus validating the antagonistic profile of the new derivatives herein documented.

### Structure–Activity Relationship Analysis

Analysis of the binding data (Tables 1–4) revealed multiple pyrazole derivatives with potent affinity and excellent selectivity for CB_1_R. Notable examples include derivatives **21b**, **21g**, and **21h** (Table 1), as well as compounds **22a**, **22b**, **22d**, **22l**, **22m**, and **22n** (Table 2). The data obtained during the functional studies with two representative ligands (**22a** and **24a**) unequivocally confirmed the antagonistic behavior (*K*_B_ = 24.7 and 77.0 nM respectively) of the tested derivatives. While series **I** produced some attractive CB_1_R ligands (e.g., Cpds **21b** and **21g**, Table 1), derivatives originating from series **II**, which feature a piperazine motif within arm 3, generally exhibited a superior CB_1_R affinity profile (Table 2). The data obtained exemplify the competitive advantages of multicomponent reaction approaches in medicinal chemistry. These pathways not only enable rapid library assembly and diversity exploration but also facilitate the development of potent ligands and the optimization of physicochemical properties relevant to BBB permeability (e.g., TPSA: 67–121 Å^2^, * *W Log *P*: 1.27–8.33). The most notable features of the structure–activity relationships (SAR) are briefly analyzed within each subset of compounds. Finally, the global SAR data will be interpreted in the context of a computational model of the CB_1_R.

We initially focused on pyrazole derivatives that featured simple amides at arm 3 (Series I, Table 1). The amide groups, with heteroatoms and hydrogen bond donors/acceptors, contributed to achieving higher polar surface area (TPSA) values. We started with two initial subsets: one with a methyl group (**20a**–**e**) and the other with hydrogen (**21a**–**e**) at R^1^. These subsets displayed variable CB_1_R affinity (Table 1) and generally negligible binding at CB_2_R. To assess the structural requirements of arm 3, particularly R^3^, and its impact on CB_1_R affinity, as well as to fine-tune key physicochemical properties, we selected six isocyanides (**17a**–**f**) with varying topologies, natures, and polarity (as shown in Scheme 1 and Table 1). A comparative analysis of the calculated TPSA and W Log *P* values of rimonabant (TPSA: 50.1 Å^2^, W Log *P*: 5.56) and the new series revealed significant modifications in physicochemical properties. Some derivatives demonstrated substantial improvements in key physicochemical descriptors (TPSA: 67–121 Å^2^, W Log *P*: 1.27–8.33). Upon reviewing the pharmacological data presented in Table 1, it became evident that subset **21** (with R^1^ = H) exhibited superior CB_1_R binding affinity compared to subset **20** (with R^1^ = Me). Notably, the methylation effect, only marginally impacting physicochemical properties (Table 1), consistently proved detrimental to CB_1_R binding (Table 1), irrespective of the residue at R^3^.

The obtained data also reveal that polar groups at R^3^ (**20d**–**f** and **21d**–**f**) are generally not well tolerated, with the ester **21d** being the only ligand exhibiting moderate affinity (*K*_*i*_ = 259 nM) and reasonable physicochemical properties (TPSA: 102.3 Å^2^, Log *P*: 4.29) in this subset. In contrast, the introduction of alkyl, aromatic, or cycloalkyl groups (*t*-Bu, Bn, Cy) at R^3^ yielded compounds (**20a**–**c** and **21a**–**c**) with modest to high affinity (*K*_*i*_ = 746–56 nM). In both subsets (R^1^ = H or Me), a cyclohexyl group proved to be the best-tolerated residue at R^3^ (e.g., **20b** and **21b**). The ligand exhibiting the highest CB_1_R affinity in this series (**21b**, *K*_*i*_ = 56.1 nM) was selected as a model compound to assess the effect of introducing assorted polar residues at R^2^, aiming to modify both affinity profiles and physicochemical features (Table 1, compounds **21g**–**21m**). As observed, all derivatives in this subset retained good affinity (*K*_*i*_ = 45.9–201 nM); however, a 2–4-fold decrease in CB_1_R affinity was noted with the introduction of the new substituent at R^2^ compared to the pattern compound (**21b**). Additionally, an incipient binding to CB_2_R was observed across the subset (Table 1). Compound **21g**, with a binding affinity in the same range (*K*_*i*_ = 45.9 nM) as the model ligand (**21b**), was an exception to this trend. Another compelling compound in this series is **21i**, showcasing satisfactory affinity (*K*_*i*_ = 101 nM) and moderate selectivity. Furthermore, it boasts appealing TPSA and CNS MPO data (96.3 and 1.96, respectively). Nonetheless, it is characterized by an excessively high lipophilicity, as indicated by a W Log *P* value of 7.1. While the modifications carried out generally enhanced the TPSA values (Table 1), the simultaneous substantial increase in Log *P* to suboptimal ranges for peripheral restriction prevents further development of this subset.

The binding affinities obtained for the twenty-eight heterocyclic amides (**22a**–**n** and **23a**–**n**) are presented in Tables 2 and 3. Each subset comprises 14 derivatives and differs in the size of the heterocyclic core (piperazine or homopiperazine) present in the amide group at position 3. Based on the promising CB_1_R data obtained during the initial exploration of the series (Compounds **22a**–**c** and **23a**–**c**, Tables 2 and 3) we further investigate the impact of the group at R^3^. This investigation involved the use of a collection of 11 isocyanides (**17**), encompassing a diverse range of structural, topological, and physicochemical features (see Tables 2 and 3). Despite the subtle structural differences between the piperazine and homopiperazine subsets and their similar physicochemical properties (Tables 2 and 3), comparative analysis of the binding data revealed that the piperazine derivatives (Table 2, Cpds **22**) consistently display higher CB_1_R affinities (3–8-fold). As observed earlier in Series **I**, lipophilic groups at R^3^ consistently demonstrated superior CB_1_R affinity profiles (Tables 2 and 3, Cpds **22a**–**d** and **23a**–**d**), while the introduction of polar residues, whether simple (esters or amides) or more structurally complex (**22h**–**k** and **23h**–**k**), typically resulted in inactive derivatives in both subsets (Tables 2 and 3, Cpds **22e**–**j** and **23e**–**k**). The morpholine derivative **22k**, with a *K*_*i*_ of 122 nM, stands as an exception to this affinity trend and exhibits physicochemical properties compatible with limited CNS exposure.

The most remarkable ligand in these series (**22a**) exhibits excellent CB_1_R affinity (*K*_*i*_ = 19.4 nM), outstanding subtype selectivity (20% at 1 μM), and maintains balanced and attractive TPSA, W Log *P* and CNS MPO data (Table 2). Similarly, derivatives **22b** and **22d** also display attractive affinity (*K*_*i*_ = 52.1 and 53.0 nM, respectively) and selectivity, albeit with a detrimental increase in lipophilicity. In addition to compound **22a**, three derivatives from this subset (**22l**, **22m**, and **22n**) captured our interest. These derivatives, designed by introducing a 4-pyridyl group in R^2^, collectively present a compelling combination of affinity and physicochemical properties, although with a deleterious increased affinity toward CB_2_R (Table 2). When compared to **22a**, the most potent derivative in this subset (*K*_*i*_ = 19.4 nM), we observe a slight decrease (2.5 times) in affinity (*K*_*i*_ = 45.8, 51.6, and 47.3 nM, respectively). However, this reduction is counterbalanced by an improvement in the values of molecular descriptors predicting peripheral restriction (Table 2). As pointed out earlier, the majority of homopiperazine derivatives (Table 3) featuring polar groups at R^3^ are inactive, which aligns with the data observed within piperazines (Table 2). In clear contrast, homopiperazine derivatives with nonpolar residues at R^3^ (Table 3, Cpds **23a**–**d**) exhibit moderate CB_1_R affinity (*K*_*i*_ = 132–425 nM). The best ligand in this subset (Cpd **23a**, *K*_*i*_ = 132 nM), featuring a *tert*-butyl group at R^3^, is 7-fold less potent than its homologue in the piperazine series (Table 2, Cpd **22a**, *K*_*i*_ = 19.4 nM). While some of these derivatives have a high W Log *P*, the presence of the amine type piperazine nitrogen atom, providing basic properties to the ligands and susceptible to protonation under physiological conditions, may help mitigate issues such as the low aqueous solubility often associated with nonpolar compounds.

As previously outlined, the design of the documented series (Tables 1–3) was guided by synthetic feasibility, docking studies, and the monitoring of the physicochemical profiles of the obtained libraries. To offer a more comprehensive and integrated analysis of the impact of physicochemical properties on BBB permeability, two distinct computational approaches were employed: the CNS MPO criteria (Tables 1–3) and a predictive model (BOILED-egg) to preliminarily estimate the pharmacokinetic profile of the series. The CNS MPO integrates diverse physicochemical parameters and is routinely employed to estimate the ability of small molecules to permeate the BBB.^53^ For instance, ligands with CNS MPO > 4 are expected to permeate the BBB, while drug candidates with CNS MPO < 3 are generally considered less likely to cross the BBB. Remarkably, most of the ligands uncovered in this study, demonstrating appealing CB_1_R affinity (as detailed in Tables 1–3), align with the CNS MPO criteria (CNS MPO < 3). Their predicted values closely resemble those reported for the second and third generations of peripherally restricted CB_1_R ligands, strongly suggesting that they are unlikely to penetrate the BBB. In terms of CNS MPO values, ligands **21g**–**m** (CNS MPO = 2.5) and **22l**–**n** (CNS MPO = 1.75) stand out as particularly appealing.^62^

BOILED-egg (Brain Or Intestinal EstimateD permeation method) is a predictive model that calculates the lipophilicity and polarity of small molecules, enabling the prediction of their brain and intestinal permeation profiles.^63^ This model relies on two physicochemical descriptors and has demonstrated its capacity to deliver swift and accurate predictions of brain access and gastrointestinal absorption, complemented by a clear and intuitive graphical representation. In this study, the collection of pyrazole derivatives presented in Tables 1–3, along with a subset of well-known CB_1_R ligands, including first-generation CB_1_R ligand Rimonabant (Figure 1), and two derivatives illustrating peripherally selective CB_1_R ligands [e.g., JD5037 (Cpd **11**), and RTICPD38 (Cpd **14**) Figure 1], underwent in silico evaluation using the BOILED-egg model. The study’s results are depicted in Figure 3. In the representation, compounds exhibiting higher affinity for the CB_1_R receptor are indicated with a larger circle. As expected, rimonabant is located within the region associated with brain penetration, consistent with its known central activity, whereas JD5037 and RTICPD38 fall outside this region, in agreement with their reported peripherally restricted profiles. Notably, all new compounds described here, with the exception of permanently charged derivatives **24** and **25**—excluded from this analysis given the limited applicability of the BOILED-Egg model to ionized species^63^—are positioned outside the yellow region, suggesting a reduced likelihood of BBB penetration.

**Figure 3:**
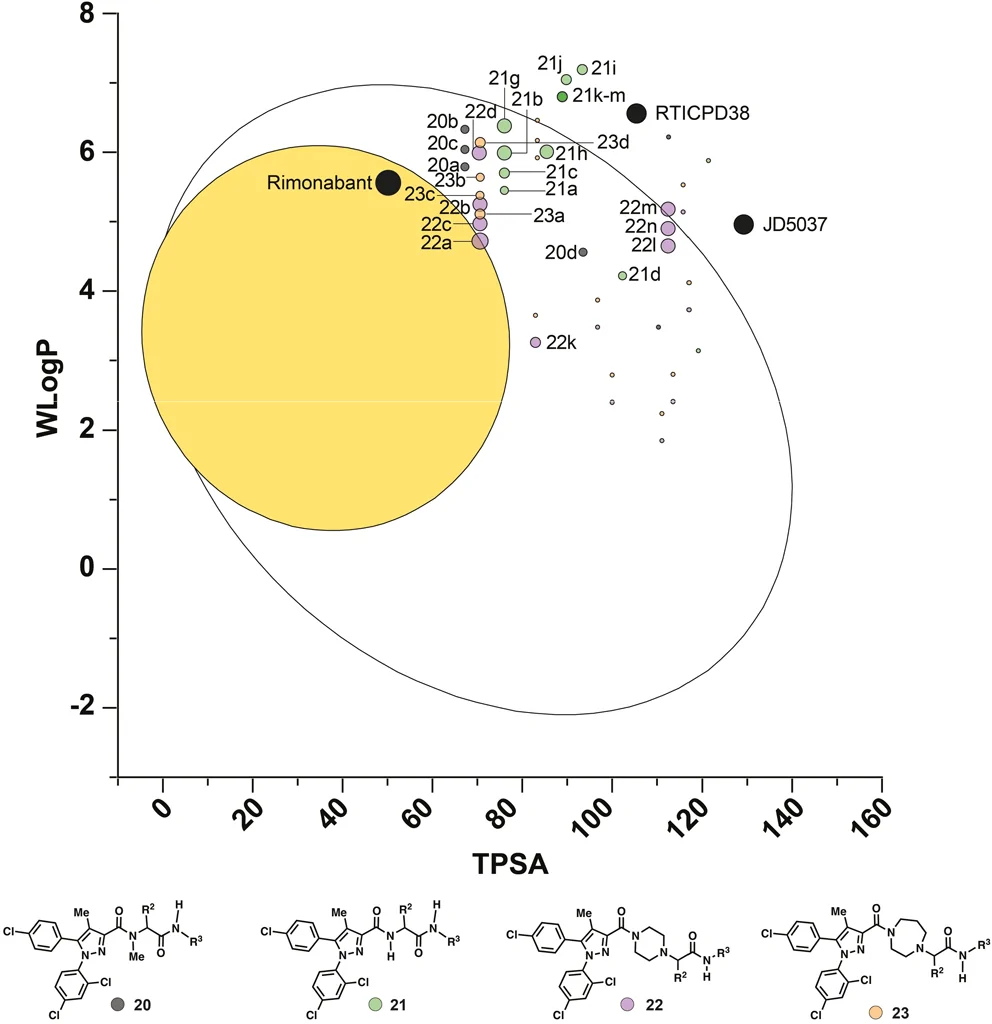
Representation of brain permeability and gastrointestinal absorption predictions by using the BOILED-egg model [W Log *P* (lipophilicity) vs TPSA (apparent polarity)]. The representation has been done with GraphPad Prism 10 using the p*K*_*i*_ for CB_1_ as the size of the represented bubble.

Overall, the integrated analysis of the affinity data and the physicochemical properties of the obtained series (Tables 1–3) revealed that piperazine and homopiperazine amides (series **II**) yielded the most attractive ligands. The CB_1_R possesses a highly lipophilic binding site; hence, CB_1_R agonists and antagonists typically are lipophilic entities. Upon scrutinizing the structures of the best-performing ligands and aligning with the objective of identifying chemical space more conducive to CNS exclusion, we explored a strategy to modify these lead compounds, with the goal of reducing their permeation across the BBB.

We then conceived the design of novel compounds (series **III**, Table 4) incorporating a permanently charged moiety within the molecule, which was expected to positively influence the selected molecular descriptors, as it is well established that charged small molecules have restricted access to the BBB.^54,55^ Therefore, we hypothesized that incorporating a positive charge (ammonium group) into the molecular scaffold would impede penetration into the brain, thereby mitigating neuropsychiatric liabilities associated with central CB_1_R occupancy. Leveraging the nucleophilic character of one of the heterocyclic nitrogen atoms in selected piperazine and homopiperazine derivatives, six ammonium salts were synthesized (Scheme 1, subsets **24** and **25**) through treatment with methyl iodide under mild conditions. Subsequently, they underwent pharmacological screening in human cannabinoid receptors (CBRs) to assess their affinity (Table 4).

As observed, the formation of heterocyclic ammonium salts led to a partial (1.5–6-fold) reduction (**24**) or complete abolition (**25**) of CB_1_R affinity, with the extent of impact dependent on the size of the group (R^3^) at the terminal amide (Table 4). Among the obtained ammonium derivatives, compound **24a** stands out by displaying reasonably good CB_1_R affinity (*K*_*i*_ = 115 nM), considering its charged nature, while maintaining high selectivity and exhibiting optimal physicochemical properties (Table 4). To the best of our knowledge, piperazinium salt **24a** represents the first example of a permanently charged nitrogen (ammonium) ligand that retains low nanomolar affinity for CB_1_R.

An integrated analysis of the CB_1_R affinity data and the physicochemical properties of the ligands obtained in this study (Tables 1–4) served as criteria for prioritizing compounds for further characterization (Table 5). While BBB permeability can be controlled by adjusting key structural parameters appropriately (e.g., TPSA, lipophilicity, etc.), enhancing affinity for efflux transporters can serve as an alternative strategy to reduce exposure to the central nervous system.^55^ P-glycoprotein (P-gp), also known as MDR1/ABCB1, stands as one of the most important drug efflux transporters expressed on the luminal side of brain capillary endothelial cells that form the blood-brain barrier. This transporter has been utilized to restrict central exposure in the development of drugs with neuropsychiatric side effects.^55,64^ For instance, it is well-documented that P-gp plays a critical role in limiting brain penetration to ensure the peripheral beneficial effects of drugs like domperidone and loperamide, which would otherwise induce unwanted side effects if distributed to the brain. P-gp transports a diverse range of substrates in an ATP-dependent manner, serving as a crucial gatekeeper of the brain.^55^

**Table 5:**
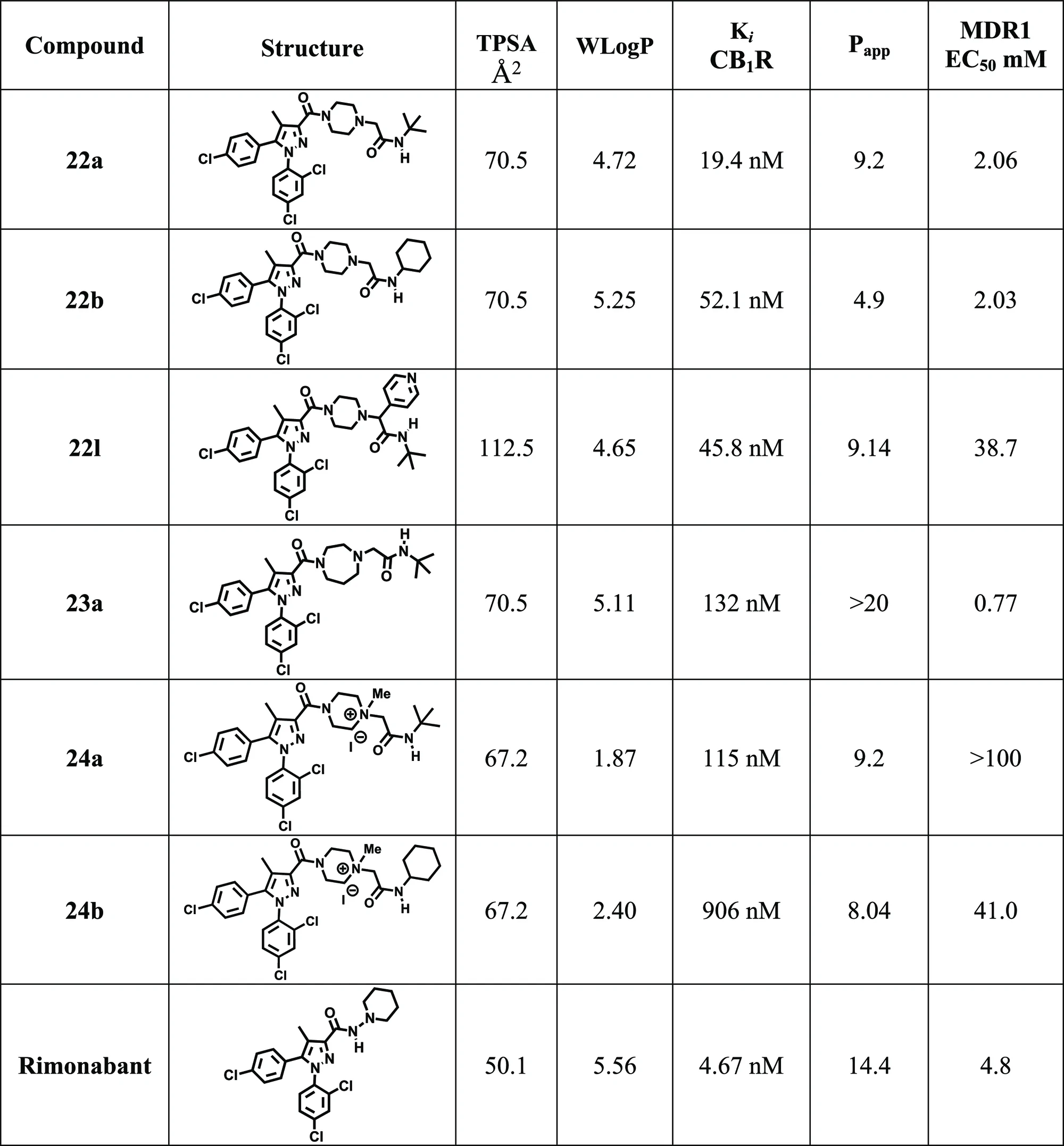
Structures, Physicochemical Properties, CB_1_R Binding Data, and Permeability Parameters for Representative Pyrazole Derivatives

### In Vitro Evaluation of the Glycoprotein-P (P-gp) Interacting Profile and Permeability

Selected derivatives (Table 5) were tested for their ability to interact with P-gp. Thus, the following experiments were performed: (i) the inhibition of the transport of the profluorescent probe Calcein-AM (P-gp substrate) in cells overexpressing P-gp (MDCK-MDR1); (ii) the apparent permeability (*P*_app_) determination in the Caco-2 cell monolayer mimicking the BBB. Calcein-AM is a P-gp substrate that, if the pump is active, is unable to enter cells, while, in the presence of a ligand interacting with P-gp, it enters cell membrane, where it is hydrolyzed by the cytosolic esterases, into the fluorescent Calcein. Calcein, being hydrophilic and not being transported by P-gp, remains in the cytosol emitting a fluorescence signal.

The apparent permeability (*P*_app_) assay measures compound transport across the polarized Caco-2 cell monolayer in both directions: from the basolateral to the apical compartment (BA) and from the apical to the basolateral compartment (AB). Since passive diffusion occurs in both directions, the BA transport mainly reflects compound permeability in the absence of P-gp-mediated efflux, being P-gp apically present. In contrast, transport in the AB direction is opposed by the presence of P-gp, which actively pumps its substrates back into the apical compartment, thereby reducing their net absorptive flux. The efflux ratio (ER = *P*_app_BA/*P*_app_AB) is therefore used to estimate the contribution of P-gp-mediated efflux. An ER > 2 indicates that the compound may behave as a P-gp substrate, whereas an ER < 2 suggests that its permeability is not significantly affected by P-gp-mediated efflux.

As observed in Table 5, the tested compounds exhibited moderate to low ability to interact with Calcein AM transport (EC_50_ ranging from 2.03 to >100 mM). Only compound **23a** interacted strongly with the pump, whereas all compounds showing a *P*_app_ > 2 were P-gp substrates. Taken together, these data suggest that the CNS exposure of the derivatives would be significantly restricted in humans, as these compounds would not be able to cross the BBB.

### Molecular Docking Simulations

To better understand the molecular aspects behind the SAR analysis, we conducted molecular docking simulations of the most intriguing compounds. For this purpose, we utilized the X-ray structure of CB_1_R, which was published in 2016 by Hua et al. and is available in the Protein Data Bank (PDB code: 5TGZ).^12^ Notably, this specific structure was chosen from the available structures in the Protein Data Bank as a suitable template for our study. The structure was resolved in complex with **AM6538** (Figure 1, Cpd **6**), one of the CB_1_R antagonists/inverse agonists that has entered clinical trials and shares a similar substructure with rimonabant (Figure 1). Consequently, it is reasonable to assume that its binding site conformation is responsible for the molecular recognition of our derivatives, supporting its appropriateness for conducting reliable docking simulations. Within this framework, JD5037 represents a relevant peripheral reference with a distinct substitution topology. Its bulky polar substituent is linked to the nitrogen atom of the dihydropyrazole core, whereas in the present analogues the pyrazole nitrogen bears the 2,4-dichlorophenyl group and structural diversification is introduced through the C3 carboxamide-containing arm 3. The compatibility of the JD5037 substitution topology with an alternative CB_1_R binding-site conformation was examined through a dedicated docking analysis using the MRI-1891-bound 9BA0 structure, as reported in the Supporting Information (Supporting Figure S1).

Figure 4 displays the top-scored docking poses of the most promising ligands from series **21**, specifically **21b** and **21g**. It is important to note that the presence of a chiral carbon atom in **21g** required accounting for both enantiomers, here referred to as (*R*)-**21g** and (*S*)-**21g**. As expected, all the compounds are predicted to share the binding mode experimentally observed for the parent antagonist **AM6538**, and previously proposed for Rimonabant (Figure 1), which is characterized by (i) well-oriented π–π interactions with F268 and F170 and (ii) ligand-protein shape complementarity.

**Figure 4:**
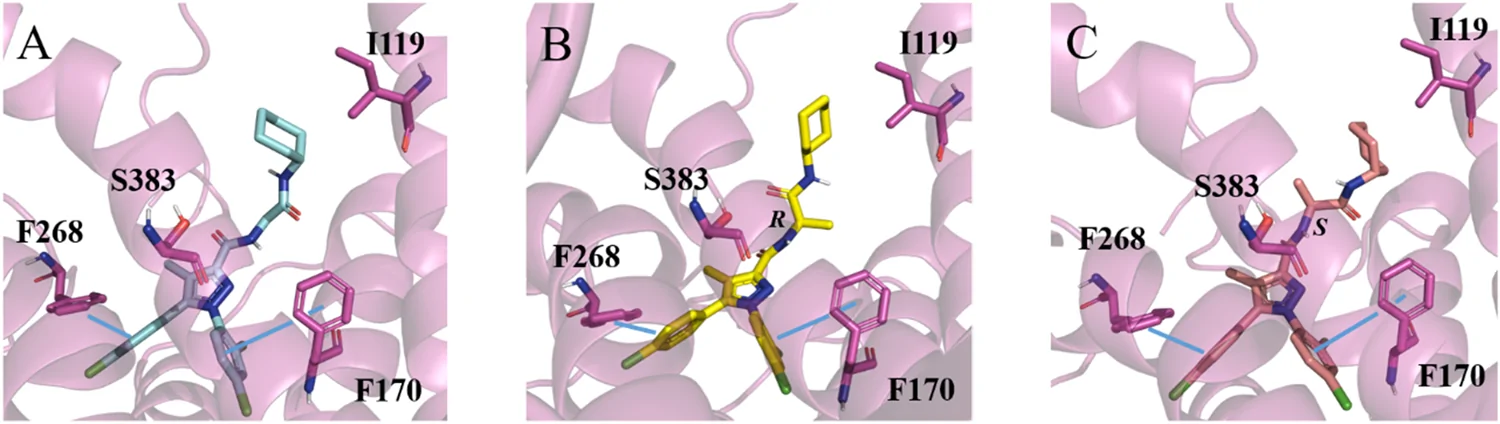
Top-scored docking poses returned by docking simulations performed on (A) **21b**, (B) (*R*)-**21g** (B) and (C) (*S*)-**21g** within the binding site of CB_1_R (PDB code: 5TGZ).^12^ Ligands and key residues are shown as sticks, whereas the protein is shown as a cartoon. π-π interactions are indicated by blue lines. For the sake of clarity, only polar hydrogen atoms are shown.

It is worth noting that the computed protein–ligand binding free energies (MMGBSA scores) align with the experimental data, indicating a slight increase in affinity toward CB_1_R after the introduction of a methyl substituent at R^2^ into the model compound **21b** (*K*_*i*_ = 56.1 nM, MMGBSA score = −91.07 kcal/mol). Specifically, (*R*)-**21g** and (*S*)-**21g** returned MMGBSA scores of −97.81 kcal/mol and −94.98 kcal/mol, respectively. To gain a deeper understanding of these results, we conducted an analysis of the individual contributions included in the total MMGBSA score. Notably, a significant portion of the computed binding energy arises from lipophilic and van der Waals interactions, reflecting the favorable shape complementarity between the ligands and the binding site. This is evident when examining the corresponding MM-GBSA lipo contributions, which are −45.57 kcal/mol, −47.22 kcal/mol, and −45.99 kcal/mol for **21b**, (*R*)-**21g**, and (*S*)-**21g**, respectively, as well as the MMGBSA VdW contributions, which amount to −73.55 kcal/mol, −71.71 kcal/mol, and −72.41 kcal/mol for **21b**, (*R*)-**21g**, and (*S*)-**21g**, respectively.

Notably, a significant difference in terms of Coulomb interactions is observed when comparing **21b** to (*R*)-**21g**, with the MM-GBSA Coulomb score being 0.17 kcal/mol for **21b** and −6.68 kcal/mol for (*R*)-**21g**. In other words, our results suggest that introduction of the methyl substituent at R^2^ does not impose a major steric penalty and may, particularly in the case of the R enantiomer, slightly enhance the electrostatic complementarity between the ligand and the receptor. Encouraged by these findings, we also conducted docking simulations of the most interesting ligand from the **22** series (**22a**, *K*_*i*_ = 19.4 nM) and its homopiperazine analogue (**23a**, *K*_*i*_ = 132 nM). As shown in Figure 5, these docking simulations reaffirm the binding mode observed in the other derivatives.

**Figure 5:**
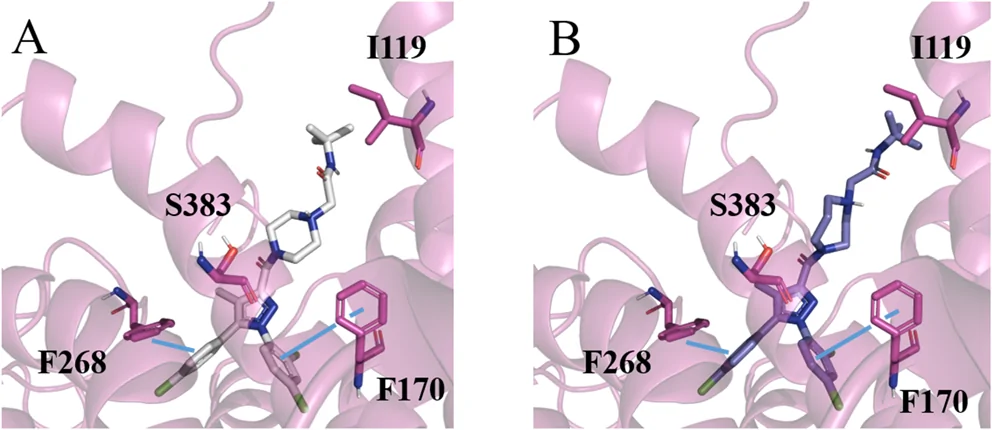
Top-scored docking poses returned by docking simulations performed on **22a** (A) and **23a** (B) within the binding site of CB_1_R (PDB code: 5TGZ).^12^ Ligands and key residues are shown as sticks, whereas the protein is shown as a cartoon. π-π interactions are indicated by blue lines. For the sake of clarity, only polar hydrogen atoms are shown.

Again, the computed protein–ligand binding free energies are consistent with the experimentally measured affinity data. Specifically, MMGBSA scores of −109.49 kcal/mol and −101.15 kcal/mol were calculated for **22a** (*K*_*i*_ = 19.4 nM) and **23a** (*K*_*i*_ = 132 nM), respectively. The analysis of the individual contributions to the total binding free energy suggests that the observed affinity difference for CB_1_R may be attributed to variations in protein–ligand electrostatic complementarity, with the MMGBSA Coulomb contribution ranging from +8.63 kcal/mol (**23a**) to +0.25 kcal/mol (**22a**). Lastly, for the sake of completeness, we conducted docking simulations of **24a**, one of the most interesting compounds presented in this study, as it represents, to the best of our knowledge, the first permanently charged CB_1_R antagonist with nanomolar affinity (*K*_*i*_ = 115 nM) and a physicochemical profile consistent with limited BBB penetration. The top-scored docking pose is displayed in Figure 6.

**Figure 6:**
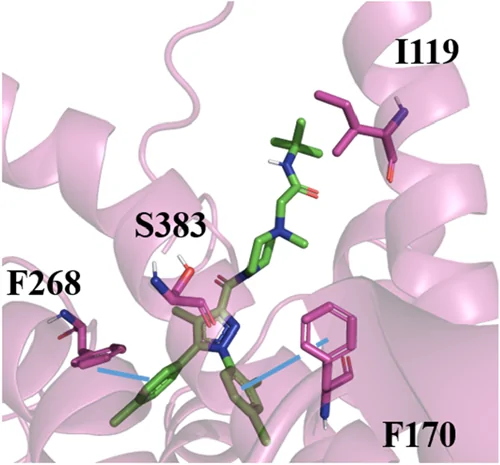
Top-scored docking poses returned by docking simulations performed on **24a** within the binding site of CB_1_R (PDB code: 5TGZ).^12^ Ligands and key residues are shown as sticks, whereas the protein is shown as a cartoon. π–π interactions are indicated by blue lines. For the sake of clarity, only polar hydrogen atoms are shown.

Consistent with the experimental data, **24a** exhibited a slightly less favorable binding free energy than **22a** (−104.03 kcal/mol vs −109.49 kcal/mol). Notably, the more unfavorable electrostatic contribution (MMGBSA Coulomb contribution of +12.78 kcal/mol for **24a** and +0.25 kcal/mol for **22a**) is offset by the observed enhancement of van der Waals interactions (MMGBSA VdW contribution of −80.73 kcal/mol for **24a** and −78.70 kcal/mol for **22a**).

### Effects of Selected CB_1_R Antagonists on Insulin Signaling and Lipolysis in Adipocytes

Given the well-established role of peripheral CB_1_R signaling in the regulation of adipocyte metabolism and insulin responsiveness, we next explored whether the most promising ligand identified in this study, **24a**, was able to modulate insulin signaling pathways in adipocytes in comparison with the reference antagonist rimonabant. For these studies, **24a** was used as its iodide salt, which was selected solely for synthetic convenience and should not be regarded as the optimal counterion form. Given the potential concerns associated with iodide, including photostability, chemical stability, and thyroid-related effects, alternative counterions will be evaluated in future studies.

The experiments were performed using differentiated adipocytes derived from the murine mesenchymal stem cell line C3H10T1/2. Two experimental conditions were analyzed: normal differentiated adipocytes (lean condition) and adipocytes with lipid hypertrophy induced by palmitate treatment, a widely used cellular model that reproduces the insulin resistance observed in obese adipose tissue. Under these conditions, the activation of the insulin signaling pathway was monitored through the phosphorylation of Akt at Ser473 after insulin stimulation. In addition, lipolytic activity was assessed by analyzing the phosphorylation of hormone-sensitive lipase (HSL) at Ser563. Representative immunoblots and densitometric analyses are shown in Figure 7.

**Figure 7:**
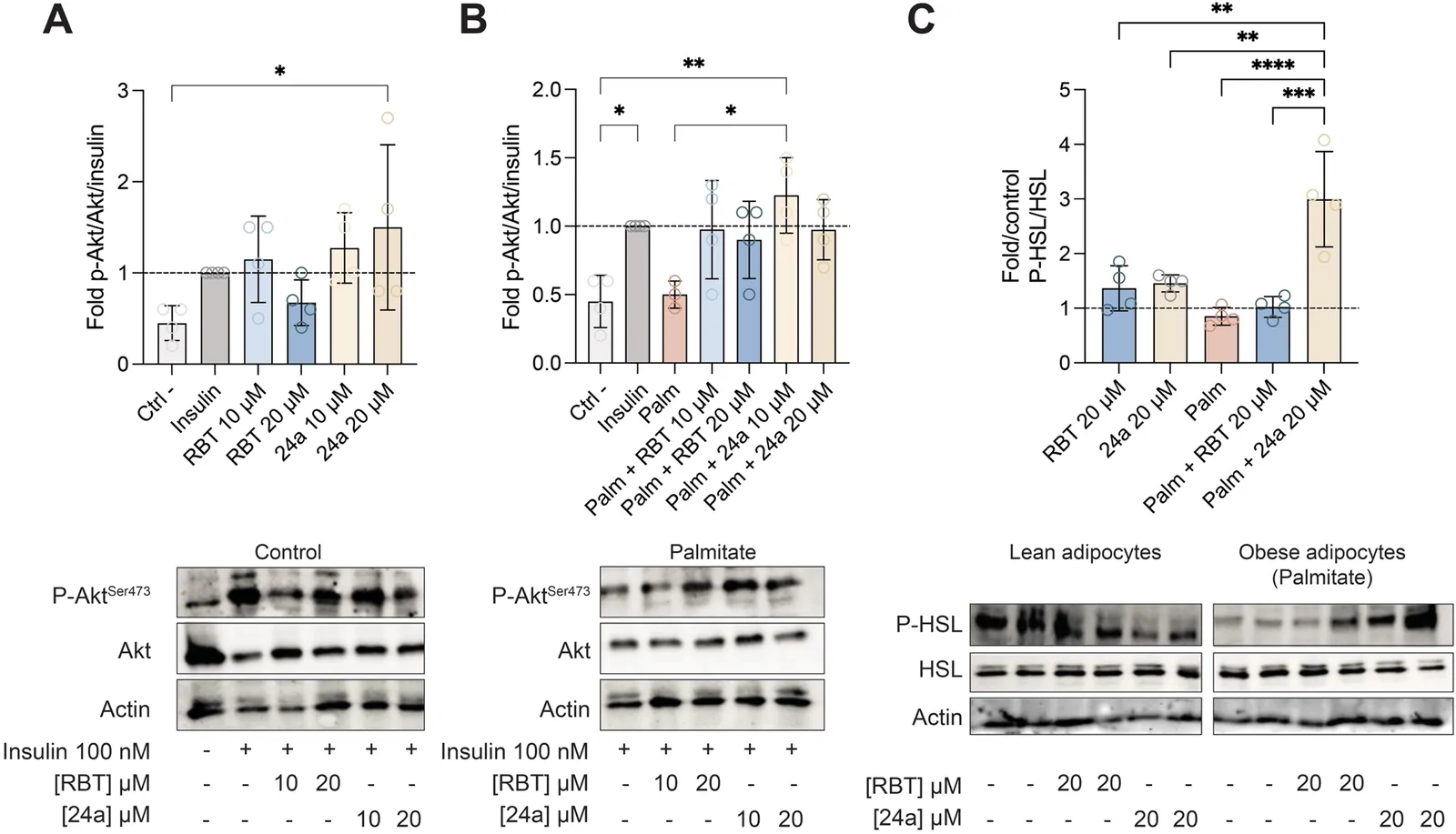
**24a** enhances insulin signaling and promotes lipolysis in adipocytes, particularly under conditions of lipid hypertrophy and insulin resistance. The impact of **24a** (10 and 20 μM) and rimonabant (RBT, 10 and 20 μM) on the insulin signaling pathway was evaluated in both control (lean) and lipid-hypertrophied/insulin-resistant (obese) adipocytes, as depicted in panels (A, B). Representative immunoblots and densitometric analysis of P-Akt (Ser473) normalized to total Akt, following insulin stimulation (100 nM, 10 min), are presented [*n* = 4 independent lysates]. The influence of **24a** and rimonabant on adipocyte lipolysis was assessed by analyzing P-HSL (Ser563) normalized to total HSL, as shown in panel (C). Statistical differences were determined using one-way ANOVA followed by Tukey’s multiple comparison test (*P* ≤ 0.05). Palm indicates palmitic acid-treated adipocytes.

As expected, insulin stimulation strongly increased Akt phosphorylation in lean adipocytes, confirming the responsiveness of the model system. In contrast, palmitate-induced lipid hypertrophy markedly impaired this response, resulting in a significant reduction of insulin-stimulated Akt phosphorylation and confirming the establishment of an insulin-resistant phenotype (Figure 7A–B). Treatment with CB_1_R antagonists restored insulin signaling to different extents depending on the compound and experimental condition. In lean adipocytes, both **24a** and rimonabant produced only modest effects on insulin signaling, although **24a** at the highest concentration tested produced a detectable enhancement of insulin-stimulated Akt phosphorylation (Figure 7A). In contrast, a more pronounced pharmacological effect was observed in lipid-hypertrophied insulin-dependent adipocytes. Under these conditions, both compounds increased insulin-stimulated Akt phosphorylation compared with untreated insulin-resistant cells; however, only **24a** achieved statistical significance at both concentrations tested (Figure 7B). Notably, **24a** produced a particularly robust effect, with a statistically significant increase in Akt phosphorylation already observed at the lower concentration tested (Figure 7B).

In addition to restoring insulin signaling, we examined whether **24a** could influence adipocyte lipolysis. As shown in Figure 7C, **24a** induced a concentration-dependent increase in p-HSL (Ser563) in lipid-hypertrophied adipocytes, indicating activation of the lipolytic pathway. This effect was particularly evident at the higher concentration tested and was markedly more pronounced than that observed with rimonabant under the same experimental conditions.

Overall, these experiments demonstrate that **24a** reproduces the key metabolic effects associated with CB_1_R antagonism in adipocytes. The compound restores insulin signaling in adipocytes with lipid hypertrophy and insulin resistance and simultaneously promotes lipolysis through activation of HSL. Importantly, the magnitude of these effects meets or exceeds that observed for rimonabant. These findings support the hypothesis that peripherally restricted CB_1_R antagonists derived from this series can retain the beneficial metabolic effects of first-generation CB_1_R blockers while avoiding the central adverse effects that limited their clinical use. The ability of **24a** to restore insulin signaling and promote lipolysis in metabolically compromised adipocytes further validates the design strategy implemented in this study and highlights this scaffold as a promising platform for the development of peripherally selective CB_1_R antagonists with potential applications in metabolic disorders such as obesity and insulin resistance. These results are particularly noteworthy in light of clinical evidence showing that rimonabant improves insulin sensitivity and glycemic control in patients with obesity and type 2 diabetes,^64^ and preclinical studies documenting that CB_1_R modulates both adipocyte insulin responsiveness^65^ and HSL-dependent lipolysis.^66,67^ Crucially, **24a** achieves these effects as a permanently charged, peripherally restricted molecule, establishing for the first time that ionic peripheral restriction is not only compatible with high CB_1_R affinity but also with the preservation of functionally relevant metabolic activity in insulin-resistant adipose tissue.

## Conclusions

Targeting CB_1_R with antagonists (or inverse agonists) with limited CNS exposure holds promise for controlling obesity and its metabolic comorbidities, while circumventing the neuropsychiatric adverse effects associated with first generation CB_1_R blockers. Drawing inspiration from the pharmacophoric model of CB_1_R ligands and employing an efficient and cost-effective Ugi-based approach, we have developed potent and subtype-selective rimonabant-like derivatives bearing polar residues at arm 3. The study highlighted the main structural features of the SAR within the series, addressing effective modulation of physicochemical properties. It also explored the functional behavior of the most intriguing ligands and provided a docking-based mechanistic rationale for the observed pharmacological data. Notably, selected derivatives showed limited propensity to interact with Calcein AM transport, displayed features consistent with P-glycoprotein (P-gp) substrate behavior, and showed in silico profiles consistent with reduced CNS exposure. Among the reported derivatives, the piperazine-1-ium derivative **24a** emerges as the first permanently charged CB_1_R antagonist, combining nanomolar affinity (*K*_*i*_ = 115 nM) with a permanently cationic nature and a physicochemical profile consistent with limited BBB penetration. Importantly, compound **24a** restores insulin signaling and enhances lipolytic activity in lipid-hypertrophied, insulin-resistant adipocytes, providing evidence that a permanently charged CB_1_R antagonist with a peripheral-targeting profile can retain the key metabolic effects associated with CB_1_R blockade. In addition to its standalone therapeutic potential, the peripheral restriction profile of **24a** positions it as a candidate for combination strategies with GLP-1R agonists, an approach that is rapidly gaining traction in the obesity and metabolic disease field. The mechanistic complementarity between peripheral CB_1_R blockade and GLP-1R activation—operating through distinct yet convergent pathways regulating energy homeostasis, insulin sensitivity, and lipid metabolism—provides a compelling rationale for such combinations. The clinical validation of this strategy through the CBeyond trial further supports the translational relevance of peripherally restricted CB1R antagonists of the type described herein. Ongoing studies include screening of pharmaceutically acceptable counterions (e.g., chloride, mesylate) to identify a salt form suitable for translational use, alongside evaluation of the metabolic impact of **24a** in relevant in vivo models of obesity and insulin resistance.

## Experimental Section

### Chemistry

Commercially available starting materials, reagents and solvents were purchased and used without further purification. The general synthetic procedure employed to prepare the noncommercially available isocyanides (**17g**–**j**) is reported in the Supporting Information. After extraction from aqueous phases, the organic solvents were dried over anhydrous sodium sulfate. The reactions were monitored by thin-layer chromatography (TLC) with 2.5 mm Merck silica gel GF 254 strips, and the purified compounds each showed a single spot; unless stated otherwise, UV light and/or iodine vapor were used for detection of compounds. The reactions were performed in coated Kimble vials on a PLS (6 × 4) Organic Synthesizer with orbital stirring. Purity and identity of all tested compounds were established by a combination of HPLC, mass spectrometry, HRMS and NMR spectra as described below. Purification of isolated products was carried out by column chromatography (Kieselgel 0.040–0.063 mm, E. Merck) or medium pressure liquid chromatography (MPLC) on a CombiFlash Companion (Teledyne ISCO) with RediSep prepacked normal-phase silica gel (35–60 μm) columns followed by recrystallization. Melting points were determined on a Gallenkamp melting point apparatus and are uncorrected. The NMR spectra were recorded on Bruker AM300 and XM500 spectrometers. Chemical shifts are given as δ values against tetramethylsilane as internal standard and J values are given in Hz. Mass spectra were obtained on a Varian MAT-711 instrument. High-resolution mass spectra were obtained on an Autospec Micromass spectrometer. Analytical HPLC was performed on a Waters Alliance system using a XSelect HSS T3 Silica 100 Å, 4.6 mm × 150 mm, 5 μm column with gradient elution using the mobile phases (A) Water and 0.1% of formic acid (B) Acetonitrile with a flow rate of 1 mL/min. The purity of all tested compounds was determined to be >95%. All compounds bearing a stereogenic center were isolated and biologically evaluated as racemates. A detailed description of synthetic methodologies as well as analytical and spectroscopic data for all described compounds is provided in the Supporting Information.

#### General Procedure for the Synthesis of Compounds **20a**–**f**

A mixture of formaldehyde **18a** (0.52 mmol), methylamine **19a** (0.52 mmol), 5-(4-chlorophenyl)-1-(2,4-dichlorophenyl)-4-methyl-1*H*-pyrazole-3-carboxylic acid **16** (0.52 mmol) and the corresponding isocyanide **17a-c/e-g** (0.52 mmol) in methanol (3 mL) was stirred with orbital stirring at room temperature for 24 h. After completion of the reaction, as indicated by TLC, dichloromethane and *p*-TsOH polymer bound (1.56 mmol) were added. The reaction mixture was stirred at room temperature until complete consumption of the unreacted isocyanide. The polystyrene resin was filtered off and subsequently washed with dichloromethane and then methanol. After the evaporation of the organic solvents, the product was isolated by column chromatography and/or preparative thin layer chromatography, using a methanol dichloromethane mixture as mobile phase.

#### General Procedure for the Synthesis of Compounds **21a**–**m**

A mixture of aldehyde **18a**–**h** (0.52 mmol), dimethoxybenzylamine **19b** (0.52 mmol), 5-(4-chlorophenyl)-1-(2,4-dichlorophenyl)-4-methyl-1*H*-pyrazole-3-carboxylic acid **16** (0.52 mmol) and the corresponding isocyanide **17a-c/e-g** (0.52 mmol) in methanol (3 mL) was stirred with orbital stirring at room temperature for 24 h. After completion of the reaction, as indicated by TLC, dichloromethane and *p*-TsOH polymer bound (1.56 mmol) were added. The reaction mixture was stirred at room temperature until complete consumption of the unreacted isocyanide. The polystyrene resin was filtered off and subsequently washed with dichloromethane and then methanol. The obtained residue after evaporation of the organic solvents was treated with trifluoroacetic acid (30% v/v) in dichloromethane and stirred overnight. NaHCO_3_ saturated solution was added to raise the pH to 8, and the water phase was extracted with dichloromethane. The organic phase was dried with Na_2_SO_4_ and purified by column chromatography and/or preparative thin layer chromatography, using a methanol dichloromethane mixture as mobile phase.

#### General Procedure for the Synthesis of Compounds **22a**–**n** and **23a**–**n**

A mixture of aldehyde **18a** or **18h** (0.52 mmol), piperazine or homopiperazine **19c**–**d** (0.52 mmol), 5-(4-chlorophenyl)-1-(2,4-dichlorophenyl)-4-methyl-1*H*-pyrazole-3-carboxylic acid **16** (0.52 mmol) and the corresponding isocyanide **17a**–**k** (0.52 mmol) in MeOH (3 mL) was stirred with orbital stirring at 60 °C for 15h. After completion of the reaction, as indicated by TLC, dichloromethane and *p*-TsOH polymer bound (1.56 mmol) were added. The reaction mixture was stirred at room temperature until complete consumption of the unreacted isocyanide. The polystyrene resin was filtered off and subsequently washed with dichloromethane and then methanol. N,N-Diisopropylethylamine (1.07 mL) was added to the solution, and the mixture was stirred at room temperature for 1h. The polymer was filtered off and washed with methanol and dichloromethane. After the evaporation of the solvent, the product was precipitated in ether, isolated and purified by column chromatography and/or preparative thin layer chromatography, using a methanol dichloromethane mixture as mobile phase.

#### General Procedure for the Synthesis of Compounds **24a**–**c** and **25a**–**c**

The corresponding compound **22a**–**c** or **23a**–**c** (0.084 mmol) was dissolved in acetonitrile (2 mL) and methyl iodide (0.84 mmol) was added. The reaction was stirred at 50 °C overnight. After the evaporation of the solvent, the product was isolated by column chromatography and/or preparative thin layer chromatography, using a methanol dichloromethane mixture as mobile phase.

### Binding Sssays

Radioligand binding competition assays of CB_1_ receptors were carried out in polypropylene 96-well plates by incubating 5 μg of membranes from Chinese hamster ovary (CHO)-CB_1_ C3 cell line (PerkinElmer) with 1 nM [^3^H]-CP55940 (PerkinElmer) and test compounds in binding buffer (50 mM Tris-HCl, 5 mM MgCl_2_, 1 mM EDTA, 0.5% BSA. pH: 7.4), as already reported.^68^ Nonspecific binding was determined in the presence of 10 μM Surinabant. The reaction mixture was incubated at 30 °C for 60 min, then 200 μL were transferred to GF/C 96-well plate (Millipore, Madrid, Spain) and washed four times with 250 μL wash buffer (50 mM Tris-HCl, 5 mM MgCl_2_, 1 mM EDTA, 0.5% BSA. pH: 7.4). Radioactivity was detected in a microplate β scintillation counter (Microbeta Trilux, PerkinElmer, Madrid, Spain). Radioligand binding competition assays of CB_2_ receptors were carried out in polypropylene 96-well plates by incubating 5 μg of membranes from Human embryonic kidney 293 cells (HEK)-CB_2_ cell line with 0.2 nM [^3^H]-CP55940 (PerkinElmer) and test compounds in binding buffer (50 mM Tris-HCl, 5 mM MgCl_2_, 2.5 mM EGTA, 0.1% BSA. pH: 7.4). Nonspecific binding was determined in the presence of 10 μM GW405833. The reaction mixture was incubated at 30 °C for 90 min, then 200 μL were transferred to GF/C 96-well plate (Millipore, Madrid, Spain) and washed four times with 250 μL wash buffer (50 mM Tris-HCl, 5 mM MgCl_2_, 2.5 mM EGTA, 1%BSA. pH: 7.4). Data was fitted to 4-parameter logistic equation by employing GraphPad Prism software (V7.0) and *K*_*i*_ data was calculated with the equation: $K_{i}=\frac{IC_{50}}{1+\left(F/K_{D}\right)}$ ; where IC_50_ was the concentration of the ligand that displaced the specific binding of the radioligand in a 50%; *K_D_* is the dissociation constant of the radioligand, and F is the concentration of the radioligand employed in the assay.

### Functional Assays

Human Cannabinoid CB_1_ receptor functional experiments were carried out in the CHO–CB_1_ C3 cell line. 48 h before the assay, 2500 cells were seeded in 100 μL culture medium with FBS dialyzed on a 96-well white plate half area (Costar 3688) and incubated for 24 h incubation in an atmosphere of 95% air and 5% CO_2_ at 37 °C. The growth medium is replaced by medium free-serum and cells were incubated for 18 h incubation in an atmosphere of 95% air and 5% CO_2_ at 30 °C. Antagonist compounds and Standard control (Rimonabant) were added and incubated for 15 min at 37 °C. CP55.940 3 nM was added in their corresponding wells and incubated for 15 min at 37 °C. Then, Forskolin 10 μM (Sigma 17018) was added and incubated for 5 min at 37 °C. Reagents from the kit (#CISBIO 62AM4PEC) were added and after incubation for 1 h at RT with gentle stirring (90 rpm) and protected from light, HTRF (λ_Ex_: 320 nm; λ_Em_: 620–665 nm) from each well was measured using a Tecan Infinite M1000 Pro.

### In Vitro Evaluation of the Glycoprotein-P (P-gp) Interacting Profile

Reagents were purchased from Celbio S.R.L (Milano, Italy). Culture Plate 96/wells plates were purchased from PerkinElmer Life Science (Waltham, MA) and Falcon (BD Biosciences, Bedford, MA). Calcein-AM was obtained from Sigma-Aldrich (Milan, Italy). **Cell cultures**. MDCK-MDR1cells are a gift of Prof. P. Borst, NKI-AVL Institute, Amsterdam, The Netherlands. Caco-2 cells were a gift of Dr. Aldo Cavallini and Dr. Caterina Messa from the Laboratory of Biochemistry, National Institute for Digestive Diseases, “S. de Bellis”, Bari (Italy). MDCK-MDR1 and Caco-2 cells were grown in DMEM high glucose supplemented with 10% fetal bovine serum, 2 mM glutamine, 100 U/mL penicillin, 100 μg/mL streptomycin, in a humidified incubator at 37 °C with a 5% CO_2_ atmosphere.

### Calcein-AM Experiment

The experiments were carried out as described by Contino et al. with minor modifications.^69^ Each cell line (30,000 cells per well) was seeded into black Culture Plate 96/wells plate with 100 μL medium and allowed to become confluent overnight. 100 μL of test compounds, solubilized in culture medium, were added to monolayers (final concentrations ranging from 0.1 to 100 μM). After a 30 min incubation time at 37 °C, Calcein-AM was added in 100 μL of Phosphate Buffered Saline (PBS) to the 96/wells plate to yield a final concentration of 2.5 μM; the plate was then incubated for 30 min. Each well was washed 3 times with ice cold PBS and saline buffer was added to each well and the plate read with Victor3 (PerkinElmer) at excitation and emission wavelengths of 485 and 535 nm, respectively. In these experimental conditions, Calcein cell accumulation in the absence and in the presence of tested compounds was evaluated and fluorescence basal level was estimated with untreated cells. In treated wells, the increase of fluorescence with respect to basal level was measured. EC_50_ values were determined by fitting the fluorescence increase percentage versus log­[dose].

### Preparation of Caco-2 Monolayer

The experiments were carried out as described by Contino et al. with minor modifications.^69^ Caco-2 cells were seeded onto a Millicell assay system (Millipore), in which a cell monolayer was set in between a filter cell and a receiver plate at a density of 20000 cells/well. The culture medium was replaced every 48 h, and the cells were kept for 21 days in culture. The trans epithelial electrical resistance (TEER) of the monolayers was measured daily, before and after the experiment, by using an epithelial voltohmmeter (Millicell-ERS). Generally, TEER values greater than 1000 Ω for a 21-day culture are considered optimal.

### Drug-Transport Experiment

After 21days of Caco-2 cell growth, the medium was removed from the filter wells and from the receiver plate, and they were filled twice with fresh Hank’s balanced salt solution (HBSS) buffer (Invitrogen). The plates were incubated at 37 °C for 30 min. After the incubation time, the HBSS buffer was removed, and drug solutions in HBSS buffer were added to the filter well at a concentration of 100 μM, whereas fresh HBSS was added to the receiver plate. The plates were incubated at 37 °C for 120 min. Afterward, samples were removed from the apical (filter well) and basolateral (receiver plate) side of the monolayer to measure their concentrations. The apparent permeability (*P*_app_), in units of nm s^–1^, was calculated by using the same equation reported below.

$$P_{\mathrm{app}}=\frac{\mathrm{VA}}{\mathrm{area}\times \mathrm{time}}\times \frac{{\left[\mathrm{drug}\right]}_{\mathrm{acceptor}}}{{\left[\mathrm{drug}\right]}_{\mathrm{initial}}}$$

in which VA is the volume (in mL) in the acceptor well, the area is the surface area of the membrane (0.11 cm^2^ of the well), time is the total transport time (7200 s), [drug]_acceptor_ is the concentration of the drug measured by UV spectroscopy, and [drug]_initial_ is the initial drug concentration (1 × 10^–4^) in the apical or basolateral wells.

### Molecular Descriptors Calculations

The partition coefficient between octanol and water (W Log *P*) and the Total Polar Surface Area (TPSA) of a series of molecules were calculated using the RDKit cheminformatics toolkit. For this purpose, the SMILES representations of the molecules were first converted into RDKit molecular objects. The W Log *P* values were then computed using RDKit’s *Crippen.MolLogP­()* function, and the TPSA values were determined using the *rdMolDescriptors.CalcTPSA­()* function in RDKit, which computes the TPSA by considering the contribution of polar atoms and their bonded hydrogens. The CNS Multiparameter Optimization (MPO) score was evaluated using InstantJChem software from Chemaxon.

### Molecular Docking Simulations

**21b, 21g, 22a, 23a** and **24a** were docked on the binding site of the X-ray structure of CB_1_R in complex with the antagonist AM6538 (resolution 2.80 Å - PDB code: 5TGZ^12^) The downloaded.pdb file was prepared using the Protein Preparation Workflow, available from the Schrodinger suite 2022–4.^70^ This tool allows reconstructing incomplete side chains, adding missing hydrogen atoms, assigning favorable protonation states at physiological pH, removing water molecules, optimizing the hydrogen bonding network and performing a force field-based minimization (OPLS-4).^71^ All the ligands subjected to docking simulations were prepared using LigPrep, available from the Schrodinger suite 2022–4. We generated all the possible tautomers and ionization states at a pH value of 7.0 ± 2.0. Grid-based ligand docking with energetics (GLIDE) was employed for docking simulations. During the docking process, full flexibility was allowed for the ligand whereas the remaining part of the protein was held fixed. Furthermore, the extra precision (XP) protocol and the default Force Field OPLS_2005 ^72^ were employed. A cubic grid having an edge of 10.0 Å (29.5 Å) for the inner (outer) box and centered on the cognate ligand AM6538 was used. This procedure was challenged by redocking the cognate ligand into its corresponding binding site. Remarkably, after applying the described docking protocol, AM6538 moved back to the original position with a root-mean-square deviation (RMSD), calculated accounting for all the heavy atoms, equal to 0.6 Å.

### MMGBSA Calculations

To perform a postdocking minimization of the obtained top-scored docking poses, the MM-GBSA method was employed using Prime,^73^ available from the Schrödinger suite 2022–4.^74^ In doing that, default settings were used and a flexibility of the residues at a maximum distance of 3 Å from the ligand was allowed. The software returned, for each compound, a score, which is an estimation of the protein–ligand binding free energy, named Δ*G* (Bind) and computed as follows

MMGBSAΔG(Bind)=Complex−Ligand−Receptor

where Complex is the energy contribution returned by the optimized ligand–receptor complex, and Ligand and Receptor are the energy contributions computed from the optimized free ligand and free receptor, respectively. Notably, more negative values of Δ*G* (Bind) are indicative of a stronger binding. Finally, to better rationalize the obtained data, an analysis of the single contributions responsible for the total MM-GBSA score (*i.e*., MMGBSA Covalent, MMGBSA Coulomb, MMGBSA Lipo, MMGBSA Hbond, MMGBSA vdw and MMGBSA Solv) was also performed.

### Cell Cultures

The murine mesenchymal stem cell line, C3H10T1/2, was generously provided by Prof. Eduardo Domínguez Medina from the USC. The cells were cultured at 37 °C with 5% CO_2_ in DMEM (4.5 g/L glucose, Corning, USA) supplemented with 10% fetal bovine serum (FBS; Sigma-Aldrich, MO, USA) and 1% penicillin and streptomycin (P/S, Biowest) until differentiation into adipocytes, as previously described.^75^ Briefly, differentiation was initiated postconfluence using an induction medium, which consisted of the maintenance medium supplemented with 1 μM dexamethasone, 0.5 mM isobutylmethylxanthine, 1 μM rosiglitazone (Sigma-Aldrich, MO, USA), and 5 μg/mL insulin (Actrapid, NovoNordisk). Two days following induction, the medium was replaced with maintenance medium containing 5 μg/mL insulin (Actrapid, NovoNordisk). Lipid hypertrophy and insulin resistance cell model was established as described previously.^76,77^ Briefly, cells were differentiated until day 6, washed three times with PBS, and incubated for 2 h in low-glucose (1 g/L) and serum-free cell culture medium. Cells were then cultured in palmitate (500 μM in 2% fatty acid-free BSA [Capricorn Scientific, USA]) in serum-free medium for 18 h. For pAkt/Akt pathway analysis, cells were washed three times in PBS and stimulated with insulin (100 nM) for 10 min. CB_1_R expression was confirmed in both lean and palmitate-treated adipocytes by Western blot (Supporting Figure S2).

### Immunoblotting

Protein extracts were prepared as previously described.^76^ In brief, protein samples were extracted through homogenization using the TissueLyser II (QIAGEN, Tokyo, Japan) in cold RIPA buffer supplemented with protease and phosphatase inhibitors (Sigma-Aldrich, St Louis, MO, USA). Thirty micrograms of cell lysates from a minimum of three independent experiments were separated on 10% SDS-PAGE gels and subsequently transferred onto nitrocellulose membranes, as previously described.^76,78^ Primary antibodies against pAkt (Ser473), Akt and pHSL (Ser563) were procured from Cell Signaling Technology (Danvers, MA, USA), while antibody against HSL were sourced from Abcam, UK. Data are presented as fold change relative to the control, adjusted for the nonphosphorylated form and actin (Invitrogen, Thermo Fisher Scientific, Massachusetts, USA), and expressed in arbitrary units in Western blots with mean ± SEM.

## Supplementary Material





## Notes

Experimental details of the synthesis and characterization of all compounds described; HPLC chromatograms for purity analysis of representative compounds (PDF)
SMILES strings, binding data and calculated physicochemical properties (XLSX)
