# Supplementary material for: Discovery of a Peripherally Directed CB1 Receptor Antagonist That Restores Insulin Signaling in Adipocytes
Source: ACS Omega. 2026 Oct 13;11(42):65239–58. doi: 10.1021/acsomega.6c07565 (PMC13721475; doi:10.1021/acsomega.6c07565)
Supplement: Supplementary file 1 [file ao6c07565_si_001.pdf]

Discovery of a Peripherally Directed CB_1 Receptor Antagonist that Restores Insulin Signaling in Adipocytes

Jhonny Azuaje,[1,2] Rubén Prieto-Díaz,[1,2] Claudia Gioé-Gallo,[1] Sandra Ortigueira,[1] Jacobo Soilán,[1] Diego Triñanes,[1] Hugo Fojo,[1] María I. Loza,[3] Marialessandra Contino,[4] Maria Grazia Perrone, [4] Giuseppe Felice Mangiatordi,[5] Pietro Delre,[5] Nerea Lago-Baameiro,[6] Adriana Estévez-López,[6] Maria Pardo,[6,7] Angela Stefanachi,[4] Giovanni Graziano,[1] José Brea,[3*] Maria Majellaro,[1,8*] and Eddy Sotelo[1,2*]

[1]Centro Singular de Investigación en Química Biolóxica e Materiais Moleculares (CIQUS), [2]Departamento de Química Orgánica, Facultade de Farmacia. Universidade de Santiago de Compostela, 15782. Santiago de Compostela, Spain. [3]Centro Singular de Investigación en Medicina Molecular e Enfermidades Crónicas (CIMUS). Universidade de Santiago de Compostela, 15782. Santiago de Compostela, Spain. [4]Dipartimento di Farmacia-Scienze del Farmaco, Università degli Studi di Bari ALDO MORO, via Orabona 4, 70125, Bari, Italy. [5]CNR – Institute of Crystallography, Via Giovanni Amendola, 122/O, 70126, Bari, Italy. [6]Grupo Obesidómica, Área de Endocrinología, Nutrición y Metabolismo. Instituto de Investigación Sanitaria de Santiago (IDIS). Complexo Hospitalario Universitario de Santiago de Compostela, [7]CIBER de Fisiopatología de la Obesidad y la Nutrición, Instituto de Salud Carlos III, Spain. [8]Celtarys Research, Avenida de Barcelona, 7. Santiago de Compostela, 15706, Spain.

Keywords: *Cannabinoid receptor CB1, peripheral CB1 antagonists, insulin resistance, adipocytes, metabolic syndrome, obesity, type 2 diabetes, endocannabinoid system, pyrazole derivatives, glucose homeostasis.*

*To whom correspondence should be addressed:
(ES) +34 881815732, Fax.: +34-881815704, e-mail: e.sotelo@usc.es
(JMB) +34 881815459, Fax.: +34-881815474, e-mail: pepo.brea@usc.es
(MM) +34 881815743, Fax.: +34-881815704, e-mail: maria.majellaro@celtarys.com

TABLE OF CONTENTS

SPECTROSCOPIC AND ANALYTICAL DATA FOR INTERMEDIATES 17G-J 3

SPECTROSCOPIC AND ANALYTICAL DATA FOR ALL COMPOUNDS DESCRIBED...........6

HPLC PURITY ANALYSIS OF REPRESENTATIVE COMPOUNDS 32

NMR SPECTRA OF REPRESENTATIVE COMPOUNDS 42

SUPPLEMENTARY FIGURES...50

REFERENCES 51

Chemistry. Commercially available starting materials, reagents and solvents were purchased and used without further purification. The general synthetic procedure employed to prepare the non-commercially available isocyanides (**17g-j**) is reported in the Supporting Information. After extraction from aqueous phases, the organic solvents were dried over anhydrous sodium sulphate. The reactions were monitored by thin-layer chromatography (TLC) with 2.5 mm Merck silica gel GF 254 strips, and the purified compounds each showed a single spot; unless stated otherwise, UV light and/or iodine vapour were used for detection of compounds. The reactions were performed in coated Kimble vials on a PLS (6X4) Organic Synthesiser with orbital stirring. Purity and identity of all tested compounds were established by a combination of HPLC, mass spectrometry, HRMS and NMR spectra as described below. Purification of isolated products was carried out by column chromatography (Kieselgel 0.040–0.063 mm, E. Merck) or medium pressure liquid chromatography (MPLC) on a CombiFlash Companion (Teledyne ISCO) with RediSep pre-packed normal-phase silica gel (35-60 μm) columns followed by recrystallization. Melting points were determined on a Gallenkamp melting point apparatus and are uncorrected. The NMR spectra were recorded on Bruker AM300 and XM500 spectrometers. Chemical shifts are given as δ values against tetramethylsilane as internal standard and J values are given in Hz. Mass spectra were obtained on a Varian MAT-711 instrument. High-resolution mass spectra were obtained on an Autospec Micromass spectrometer. Analytical HPLC was performed on a Waters Alliance system using a XSelect HSS T3® Silica 100 Å, 4.6 mm × 150 mm, 5 μm column with gradient elution using the mobile phases (A) Water and 0.1% of formic acid (B) Acetonitrile with a flow rate of 1 mL/min. The purity of all tested compounds was determined to be >95%. A detailed description of synthetic methodologies as well as analytical and spectroscopic data for all described compounds is provided in the Supporting Information.

General procedure for the synthesis of isocyanides 17g-j

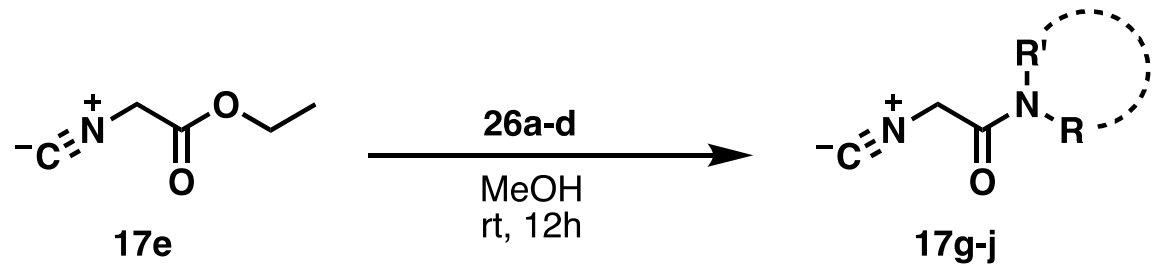

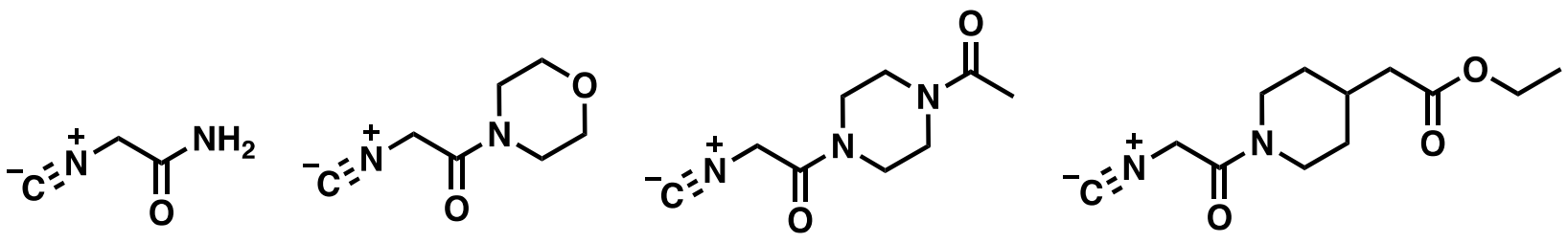

17g 17h 17i 17j

Followed the procedure in the literature.[1] A mixture of methyl 2-isocyanoacetate **17e** (1 mmol) and the corresponding amine **26a** (ammonia), **26b** (morpholine), **26c** (1-(piperazin-1-yl)ethan-1-one), or **26d** (ethyl 2-(piperidin-4-yl)acetate) (1.5 mmol) in methanol (3 mL) was stirred at room temperature for 12 h. After completion of the reaction, as indicated by TLC, the solvent was evaporated, and the product was isolated by column chromatography using a methanol dichloromethane mixture (1% MeOH in DCM) as mobile phase.

Spectroscopic and analytical data for intermediates 17g-j

2-isocyanoacetamide (17g): Yield: 62%. ^{1}H NMR (300 MHz, DMSO-d_6) δ 7.54 (s, 1H, NH_2), 7.35 (s, 1H, NH_2), 4.27 (s, 2H, -CH_2CON-).

2-isocyano-1-morpholinoethan-1-one (17h): Yield: 70%. ^{1}H NMR (300 MHz, $CDCl_3$) δ 4.28 (s, 2H, -CH_2CON-), 3.76–3.68 (m, 4H, CO(NC_4H_8O)), 3.67–3.59 (m, 2H, CO(NC_4H_8O)), 3.46 – 3.36 (m, 2H, CO(NC_4H_8O)).

1-(4-acetylpiperazin-1-yl)-2-isocyanoethan-1-one (17i): Yield: 66%. ^{1}H NMR (300 MHz, $CDCl_3$) δ 4.33 (s, 2H, -CH_2CON-), 3.76–3.34 (m, 8H, CO(NC_4H_8N)), 2.15 (s, 3H, $COCH_3$).

Ethyl 2-(1-(2-isocyanoacetyl)piperidin-4-yl)acetate (17j): Yield: 73%. ^{1}H NMR (300 MHz, $CDCl_3$) δ 4.61–4.50 (m, 1H, -CO(NC_5H_9)CH_2CO-), 4.28 (s, 2H, -CH_2CON-), 4.15 (q, *J* = 7.1 Hz, 2H, COOCH_2CH_3), 3.68–3.57 (m, 1H, -CO(NC_5H_9)CH_2CO-), 3.20–3.05 (m, 1H, -CO(NC_5H_9)CH_2CO-), 2.81–2.59 (m, 1H, -CO(NC_5H_9)CH_2CO-), 2.31–2.23 (m, 2H, -CO(NC_5H_9)CH_2CO-), 2.16–1.98 (m, 1H, -CO(NC_5H_9)CH_2CO-), 1.93–1.74 (m, 2H, -CO(NC_5H_9)CH_2CO-), 1.27 (m, 5H, -CO(NC_5H_9)CH_2CO-, COOCH_2CH_3).

General Procedure for the synthesis of compounds 20a-f: A mixture of formaldehyde **18a** (0.52 mmol), methylamine **19a** (0.52 mmol), 5-(4-chlorophenyl)-1-(2,4-dichlorophenyl)-4-methyl-1H-pyrazole-3-carboxylic acid **16** (0.52 mmol) and the corresponding isocyanide **17a-c/e-g** (0.52 mmol) in methanol (3 mL) was stirred with orbital stirring at room temperature for 24 h. After completion of the reaction, as indicated by TLC, dichloromethane and *p*-TsOH polymer bound (1.56 mmol) were added. The reaction mixture was stirred at room temperature until complete consumption of the unreacted isocyanide. The polystyrene resin was filtered off and subsequently washed with dichloromethane and then methanol. After the evaporation of the organic solvents, the product was isolated by column chromatography and/or preparative thin layer chromatography, using a methanol dichloromethane mixture as mobile phase.

General Procedure for the synthesis of compounds 21a-m: A mixture of aldehyde **18a-h** (0.52 mmol), dimethoxybenzylamine **19b** (0.52 mmol), 5-(4-chlorophenyl)-1-(2,4-dichlorophenyl)-4-methyl-1H-pyrazole-3-carboxylic acid **16** (0.52 mmol) and the corresponding isocyanide **17a-c/e-g** (0.52 mmol) in methanol (3 mL) was stirred with orbital stirring at room temperature for 24 h. After completion of the reaction, as indicated by TLC, dichloromethane and *p*-TsOH polymer bound (1.56 mmol) were added. The reaction mixture was stirred at room temperature until complete consumption of the unreacted isocyanide. The polystyrene resin was filtered off and subsequently washed with dichloromethane and then methanol. The obtained residue after evaporation of the organic solvents

was treated with trifluoroacetic acid (30% v/v) in dichloromethane and stirred overnight. $NaHCO_3$ saturated solution was added to raise the pH to 8 and the water phase was extracted with dichloromethane. The organic phase was dried with Na_2SO_4 and purified by column chromatography and/or preparative thin layer chromatography, using a methanol dichloromethane mixture as mobile phase.

General Procedure for the synthesis of compounds 22a-n and **23a-n**: A mixture of aldehyde **18a** or **18h** (0.52 mmol), piperazine or homopiperazine **19c-d** (0.52 mmol), 5-(4-chlorophenyl)-1-(2,4-dichlorophenyl)-4-methyl-1H-pyrazole-3-carboxylic acid **16** (0.52 mmol) and the corresponding isocyanide **17a-k** (0.52 mmol) in MeOH (3 mL) was stirred with orbital stirring at 60 ºC for 15h. After completion of the reaction, as indicated by TLC, dichloromethane and *p*-TsOH polymer bound (1.56 mmol) were added. The reaction mixture was stirred at room temperature until complete consumption of the unreacted isocyanide. The polystyrene resin was filtered off and subsequently washed with dichloromethane and then methanol. N,N-Diisopropylethylamine (1.07 mL) was added to the solution and the mixture was stirred at room temperature for 1h. The polymer was filtered off and washed with methanol and dichloromethane. After the evaporation of the solvent, the product was precipitated in ether, isolated and purified by column chromatography and/or preparative thin layer chromatography, using a methanol dichloromethane mixture as mobile phase.

General Procedure for the synthesis of compounds 24a-c and **25a-c***:* The corresponding compound **22a-c** or **23a-c** (0.084 mmol) was dissolved in acetonitrile (2 mL) and methyl iodide (0.84 mmol) was added. The reaction was stirred at 50 ºC overnight. After the evaporation of the solvent, the product was isolated by column chromatography and/or preparative thin layer chromatography, using a methanol dichloromethane mixture as mobile phase.

Spectroscopic and analytical data for all compounds described.

***N*-(2-(*tert*-butylamino)-2-oxoethyl)-5-(4-chlorophenyl)-1-(2,4-dichlorophenyl)-*N*,4-dimethyl-1*H*-pyrazole-3-carboxamide (20a):** Yield: 40%; Mp: 159 – 160ºC. ^{1}H NMR (300 MHz, $CDCl_3$) δ 7.50 – 7.35 (m, 1H, Ar-H), 7.30 (d, *J* = 8.3 Hz, 2H, Ar-H), 7.27 – 7.22 (m, 1H, Ar-H), 7.20 – 7.08 (m, 1H, Ar-H), 7.06 (d, *J* = 8.5 Hz, 2H, Ar-H), 6.38 (br s, 1H, NH), 4.06 (s, 2H, -N(CH_3)CH_2-), 3.34 (s, 1H, NCH_3), 3.14 (s, 2H, NCH_3), 2.22 (s, 3H, Ar-CH_3), 1.34 (s, 3H, NHC(CH_3)$_3$), 1.13 (s, 6H, NHC(CH_3)$_3$). ^{13}C NMR (75 MHz, $CDCl_3$) δ: 168.0, 164.5, 145.7, 142.5, 136.1, 135.6, 135.2, 133.1, 130.7, 130.6, 130.4, 130.3, 129.0, 127.9, 126.8, 117.7, 57.1, 51.0, 34.0, 28.7, 28.3, 9.1. LRMS (CI) calcd. for $C_{24}H_{26}Cl_3N_4O_2$ $[M+H]^+$: 507.11 found: 507.14.

5-(4-chlorophenyl)-*N*-(2-(cyclohexylamino)-2-oxoethyl)-1-(2,4-dichlorophenyl)-*N*,4-dimethyl-1*H*-pyrazole-3-carboxamide (20b): Yield: 38%; Mp: 135 – 137ºC. ^{1}H NMR (300 MHz, $CDCl_3$) δ 7.53 – 7.37 (m, 1H, Ar-H), 7.31 (d, *J* = 8.3 Hz, 2H, Ar-H), 7.26 (dd, *J* = 8.5, 2.2 Hz, 1H, Ar-H), 7.19 – 7.10 (m, 1H, Ar-H), 7.08 (d, *J* = 8.4 Hz, 2H, Ar-H), 6.48 (br s, 1H, NH), 4.15 (s, 2H, -N(CH_3)CH_2-), 3.90 – 3.62 (m, 1H, N(*CH*-C_5H_{10})), 3.35 (s, 1H, NCH_3), 3.14 (s, 2H, NCH_3), 2.24 (s, 3H, Ar-CH_3), 2.03 – 1.40 (m, 5H, NH(CH-C_5H_{10})), 1.35 – 1.12 (m, 3H, NH(CH-C_5H_{10})), 1.08 – 0.78 (m, 2H, NH(CH-C_5H_{10})). ^{13}C NMR (75 MHz, $CDCl_3$) δ 167.9, 164.6, 145.6, 142.5, 136.2, 135.7, 135.2, 133.2, 130.7, 130.4, 130.3, 129.0, 127.8, 126.8, 117.8, 56.3, 47.7, 32.4, 25.5, 24.2, 9.1. LRMS (CI) calcd. for $C_{26}H_{28}Cl_3N_4O_2$ $[M+H]^+$: 533.13 found: 533.11.

N-(2-(benzylamino)-2-oxoethyl)-5-(4-chlorophenyl)-1-(2,4-dichlorophenyl)-N,4-dimethyl-1H-pyrazole-3-carboxamide (20c): Yield: 43%; Mp: 153 – 155ºC. ^{1}H NMR (300 MHz, $CDCl_3$) [rotamers mixture] δ 7.93 (br s, 1H, NH), 7.49 – 6.87 (m, 12 H, Ar-H), 4.57 – 4.32 (m, 2H, CH_2-Ph), 4.25 (s, 2H, -N(CH_3)$\mathit{CH_2}$-), 3.38 (s, 1H, NCH_3), 3.18 (s, 2H, NCH_3), 2.29 – 2.10 (m, 3H, Ar-CH_3). ^{13}C NMR (75 MHz, $CDCl_3$) δ 168.8, 164.4, 145.5, 142.5, 137.7, 135.9, 135.1, 132.6, 130.6, 130.4, 130.0, 129.0, 128.4, 127.9, 127.5, 127.2, 126.8, 118.0, 56.0, 43.5, 34.2, 9.1. LRMS (CI) calcd. for $C_{27}H_{24}Cl_3N_4O_2$ $[M+H]^+$: 541.10 found: 541.14.

Ethyl N-(5-(4-chlorophenyl)-1-(2,4-dichlorophenyl)-4-methyl-1H-pyrazole-3-carbonyl)-N-methylglycylglycinate (20d): Yield: 48%; Mp: 179 – 181 ºC. ^{1}H NMR (300 MHz, $CDCl_3$) δ 7.81 (br s, 1H, NH), 7.44 (d, J = 2.2 Hz, 1H, Ar-H), 7.31 (d, J = 8.5 Hz, 2H, Ar-H), 7.27 – 7.21 (m, 1H, Ar-H), 7.20 – 7.13 (m, 1H, Ar-H), 7.07 (d, J = 8.3 Hz, 2H, Ar-H), 4.37 – 3.90 (m, 4H, COO($\mathit{CH_2}CH_3$), -N(CH_3)$\mathit{CH_2}$-), 3.74 (s, 1H, -NH$\mathit{CH_2}$CO-), 3.64 (s, 1H, -NH$\mathit{CH_2}$CO-), 3.39 (s, 1H, NCH_3), 3.18 (s, 2H, NCH_3), 2.24 (s, 3H, Ar-CH_3), 1.35 – 1.13 (m, 3H, COO($CH_2\mathit{CH_3}$)). ^{13}C NMR (75 MHz, $CDCl_3$) δ 169.2, 165.7, 164.6, 145.6, 142.4, 135.8, 135.5, 135.0, 132.8, 130.7, 130.5, 130.3, 129.0, 127.9, 127.0, 118.0, 61.3, 41.2, 41.1, 34.3, 14.1, 9.1. LRMS (CI) calcd. for $C_{24}H_{24}Cl_3N_4O_4$ $[M+H]^+$: 537.09 found: 537.06

Diethyl ((2-(5-(4-chlorophenyl)-1-(2,4-dichlorophenyl)-N,4-dimethyl-1H-pyrazole-3-carboxamido)acetamido)methyl)phosphonate (20e): Yield: 30%; Mp: 179 – 181ºC.^{1}H NMR (300 MHz, $CDCl_3$) δ 7.55 (br s, 0.5H, NH), 7.44 (d, *J* = 2.2 Hz, 1H, Ar-H), 7.30 (d, *J* = 8.3 Hz, 2H, Ar-H), 7.27 – 7.22 (m, 1H, Ar-H), 7.20 – 7.11 (m, 1H, Ar-H), 7.05 (d, *J* = 8.1 Hz, 2H, Ar-H), 6.97 (br s, 0.5H, NH), 4.35 – 4.18 (m, 2H, -N(CH_3)$\mathit{CH_2}$-), 4.16 – 3.97 (m, 4H, PO(O$\mathit{CH_2}$$CH_3$)$_2$), 3.80 – 3.54 (m, 2H, NH$\mathit{CH_2}$PO(OCH_2CH_3)$_2$), 3.36 (s, 1H, NCH_3), 3.14 (s, 2H, NCH_3), 2.23 (s, 3H, Ar-CH_3), 1.38 – 1.13 (m, 6H, PO($OCH_2$$\mathit{CH_3}$)$_2$). ^{13}C NMR (75 MHz, $CDCl_3$) δ 168.7, 165.6, 145.6, 142.4, 142.2, 135.9, 135.5, 135.1, 133.0, 132.7, 130.7, 130.4, 129.0, 128.0, 127.1, 127.0, 62.5, 55.5, 38.6, 34.7, 16.4, 16.3, 9.1. LRMS (CI) calcd. for $C_{25}H_{29}Cl_3N_4O_5P$ $[M+H]^+$: 601.10 found: 601.13.

***N*-(2-((2-amino-2-oxoethyl)amino)-2-oxoethyl)-5-(4-chlorophenyl)-1-(2,4-dichlorophenyl)-*N*,4-dimethyl-1*H*-pyrazole-3-carboxamide (20f):** Yield: 42%; Mp: 100-102ºC. ^{1}H NMR (300 MHz, $CDCl_3$) δ 7.75 (t, *J* = 5.8 Hz, 1H, N*H*CH_2), 7.44 (d, *J* = 2.1 Hz, 1H, Ar-H), 7.41 – 7.14 (m, 4H, Ar-H), 7.07 (d, *J* = 8.1 Hz, 2H, Ar-H), 6.33 (br s, *J* = 256.5 Hz, 2H, -CH_2CON$\mathit{H_2}$), 4.22 (d, *J* = 5.6 Hz, 2H, -C$\mathit{H_2}$CONH_2), 3.91 (s, 2H, -N(CH_3)C$\mathit{H_2}$-), 3.29 (s, 3H, -N(C$\mathit{H_3}$)CH_2-), 2.19 (s, 3H, CH_3). ^{13}C NMR (75 MHz, $CDCl_3$) δ 171.8, 169.2, 165.2, 145.6, 145.5, 136.5, 135.3, 135.1, 132.9, 130.7, 130.5, 129.0, 128.2, 127.9, 118.0, 55.7, 42.7, 39.3, 35.2, 9.1. LRMS (CI) calcd. For $C_{22}H_{21}Cl_3N_5O_3$ $[M+H]^+$: 507.06 found: 507.10.

***N*-(2-(tert-butylamino)-2-oxoethyl)-5-(4-chlorophenyl)-1-(2,4-dichlorophenyl)-4-methyl-1H-pyrazole-3-carboxamide (21a):** Yield: 35%; Mp: 198 – 200ºC. ^{1}H NMR (300 MHz, $CDCl_3$) δ 7.49 (br t, *J* = 5.7 Hz, 1H, Ar-H), 7.42 (d, *J* = 2.0 Hz, 1H, Ar-H), 7.33 – 7.25 (m, 4H, Ar-H), 7.09 – 7.03 (m, 2H, Ar-H), 5.90 (br s, 1H, NH), 3.99 (d, *J* = 5.8 Hz, 2H, -NHCH_2-), 2.36 (s, 3H, Ar-CH_3), 1.36 (s, 9H, NHC(CH_3)$_3$). ^{13}C NMR (75 MHz, $CDCl_3$) δ 168.0, 163.0, 144.5, 143.0, 135.9, 134.9, 132.8, 130.8, 130.6, 130.2, 128.9, 127.8, 127.1, 117.6, 51.4, 43.4, 28.7, 9.4. LRMS (CI) calcd. for $C_{23}H_{24}Cl_3N_4O_2$ $[M+H]^+$: 493.10 found: 493.10.

5-(4-chlorophenyl)-*N*-(2-(cyclohexylamino)-2-oxoethyl)-1-(2,4-dichlorophenyl)-4-methyl-1H-pyrazole-3-carboxamide (21b): Yield: 37%; Mp: 244 – 246 ºC. ^{1}H NMR (300 MHz, $CDCl_3$) δ 7.52 (br t, *J* = 5.8 Hz, 1H, NH), 7.41 (d, *J* = 1.9 Hz, 1H, Ar-H), 7.32 – 7.28 (m, 2H, Ar-H), 7.28-7.24 (m, 2H, Ar-H), 7.09 – 6.99 (m, 2H, Ar-H), 6.22 (br s, 0.5H, NH), 6.19 (br s, 0.5H, NH), 4.06 (d, *J* = 5.9 Hz, 2H, -NHCH_2-), 3.89 – 3.67 (m, 1H, NH(CH-C_5H_{10})), 2.35 (s, 3H, Ar-CH_3), 1.98 – 1.83 (m, 3H, NH(CH-C_5H_{10})), 1.76 – 1.53 (m, 3H, NH(CH-C_5H_{10})), 1.45 – 1.25 (m, 2H, NH(CH-C_5H_{10})), 1.23 – 1.08 (m, 3H, NH(CH-C_5H_{10})). ^{13}C NMR (75 MHz, $CDCl_3$) δ 167.9, 163.2, 144.3, 143.1, 136.0, 135.8, 135.0, 132.8, 130.8, 130.5, 130.3, 128.9, 127.9, 127.1, 117.7, 48.4, 43.2, 32.9, 25.5, 24.8, 9.4. HRMS (ESI) calcd. for $C_{25}H_{26}Cl_3N_4O_2$ $[M+H]^+$: 519.1116 found: 519.1115.

***N*-(2-(benzylamino)-2-oxoethyl)-5-(4-chlorophenyl)-1-(2,4-dichlorophenyl)-4-methyl-1H-pyrazole-3-carboxamide (21c):** Yield: 39%; Mp: 260 – 262ºC. ^{1}H NMR (300 MHz, DMSO-d_6) δ 8.39 (br t, *J* = 5.7 Hz, 1H, NH), 8.30 (br t, *J* = 5.9 Hz, 1H, NH), 7.77 (d, *J* = 2.1 Hz, 1H, Ar-H), 7.71 (d, *J* = 8.6 Hz, 1H, Ar-H), 7.56 (dd, *J* = 8.5, 2.1 Hz, 1H, Ar-H), 7.45 (d, *J* = 8.7 Hz, 2H, Ar-H), 7.34 – 7.19 (m, 7H, Ar-H), 4.29 (d, *J* = 5.8 Hz, 2H, CH_2-Ph), 3.89 (d, *J* = 6.2 Hz, 2H, -NHCH_2CO-), 2.24 (s, 3H, Ar-CH_3). ^{13}C NMR (75 MHz, DMSO-d_6) δ 169.2, 162.6, 144.9, 143.0, 139.8, 136.2, 135.5, 134.2, 132.5, 132.3, 131.7, 130.1, 129.2, 128.8, 128.7, 127.7, 127.2, 117.0, 42.5, 42.4, 9.6. LRMS (CI) calcd. for $C_{26}H_{22}Cl_3N_4O_2$ $[M+H]^+$: 527.08 found: 527.09.

Ethyl (5-(4-chlorophenyl)-1-(2,4-dichlorophenyl)-4-methyl-1H-pyrazole-3-carbonyl)-glycylglycinate (21d): Yield: 42%; Mp: 181 – 183ºC. ^{1}H NMR (300 MHz, $CDCl_3$) δ 7.51 (br t, *J* = 6.0 Hz, 1H, NH), 7.46 – 7.39 (m, 1H, Ar-H), 7.35 – 7.25 (m, 4H, Ar-H), 7.06 (d, *J* = 8.5 Hz, 2H, Ar-H), 6.74 (br s, 1H, NH), 4.20 (q, *J* = 7.3 Hz, 2H, COO($CH_2$$CH_3$)), 4.15 (d, *J* = 5.9 Hz, 2H, NHCH_2COO(CH_2CH_3)), 4.05 (d, *J* = 5.2 Hz, 2H, -NHCH_2CONH-), 2.36 (s, 3H, Ar-CH_3), 1.27 (t, *J* = 7.2 Hz, 3H, COO($CH_2$$CH_3$)). ^{13}C NMR (75 MHz, $CDCl_3$) δ 169.7, 169.3, 163.3, 144.1, 143.2, 136.1, 135.8, 135.0, 132.9, 130.8, 130.5, 130.3, 128.9, 127.9, 127.0, 118.0, 61.6, 42.9, 41.3, 14.1, 9.3. LRMS (CI) calcd. for $C_{23}H_{22}Cl_3N_4O_4$ $[M+H]^+$: 523.07 found: 523.07.

Diethyl ((2-(5-(4-chlorophenyl)-1-(2,4-dichlorophenyl)-4-methyl-1H-pyrazole-3 carboxa-mido)acetamido)methyl)phosphonate (21e): Yield: 31%; Mp: 186 – 188ºC. ^{1}H NMR (300 MHz, $CDCl_3$) δ 7.68 (br t, *J* = 6.0 Hz, 1H, NH), 7.44 – 7.36 (m, 2H, Ar-H), 7.35 – 7.24 (m, 3H, Ar-H), 7.06 (d, *J* = 8.1 Hz, 2H, Ar-H), 6.96 (br s, 1H, NH), 4.20 – 4.02 (m, 6H, PO(O$CH_2$$CH_3$)$_2$, -NH$CH_2$CO-), 3.64 (dd, *J* = 12.1, 5.9 Hz, 2H, NHCH_2PO(OCH_2CH_3)$_2$), 2.30 (s, 3H, Ar-CH_3), 1.29 (t, *J* = 7.0 Hz, 6H, PO($OCH_2$$CH_3$)$_2$). ^{13}C NMR (75 MHz, $CDCl_3$) δ 169.1, 163.3, 144.1, 143.3, 136.1, 135.7, 135.0, 132.9, 130.8, 130.7, 130.2, 128.9, 127.9, 127.0, 117.9, 62.8, 62.7, 43.0, 34.6, 16.4, 16.3, 9.3. LRMS (CI) calcd. for $C_{24}H_{27}Cl_3N_4O_5P$ $[M+H]^+$: 587.08 found: 587.07.

***N*-(2-((2-amino-2-oxoethyl)amino)-2-oxoethyl)-5-(4-chlorophenyl)-1-(2,4-dichlorophenyl)-4-methyl-1*H*-pyrazole-3-carboxamide (21f):** Yield: 34%; Mp: 140-142ºC. ^{1}H NMR (300 MHz, $CDCl_3$) δ 7.56 (br t, *J* = 5.1 Hz, 1H, -CON*H*CH_2-), 7.48 – 7.44 (m, 1H, Ar-H), 7.37 – 7.22 (m, 2H, Ar-H), 7.19 (d, *J* = 7.4 Hz, 2H, Ar-H), 7.08 (d, *J* = 8.3 Hz, 2H, Ar-H), 6.61 (br s, *J* = 156.0 Hz, 2H, -CH_2CONH_2), 5.42 (s, 1H, -CON*H*(CH_2)-), 4.12 (d, *J* = 5.7 Hz, 2H, -CONH(CH_2)-), 4.02 (d, *J* = 5.8 Hz, 2H, -CH_2CONH_2), 2.37 (s, 3H, CH_3). ^{13}C NMR (75 MHz, $CDCl_3$) δ 171.7, 168.2, 164.9, 143.5, 145.5, 136.5, 135.4, 135.1, 133.2, 130.7, 130.5, 129.0, 128.2, 127.9, 118.0, 55.7, 42.6, 41.2, 9.8. LRMS (CI) calcd. For $C_{21}H_{19}Cl_3N_5O_3$ $[M+H]^+$: 493.05 found: 493.05

(±) 5-(4-chlorophenyl)-*N*-(1-(cyclohexylamino)-1-oxopropan-2-yl)-1-(2,4-dichlorophenyl)-4-methyl-1H-pyrazole-3-carboxamide (21g): Yield: 44%; Mp: 138 – 139ºC. ^{1}H NMR (300 MHz, $CDCl_3$) δ 7.42 (s, 1H, Ar-H), 7.36 – 7.23 (m, 5H, Ar-H), 7.06 (d, *J* = 8.5 Hz, 2H, Ar-H), 6.16 (br s, 1H, NH), 4.58 (p, *J* = 7.0 Hz, 1H, -NH*CH*(CH_3)CO-), 3.83 – 3.68 (m, 1H, NH(*CH*-C_5H_{10})), 2.36 (s, 3H, Ar-CH_3), 2.00 – 1.80 (m, 2H, NH(CH-C_5H_{10})), 1.76 – 1.54 (m, 4H, NH(CH-C_5H_{10})), 1.46 (d, *J* = 7.0 Hz, 3H, -NHCH(CH_3)CO-), 1.42 – 1.05 (m, 4H, NH(CH-C_5H_{10})). ^{13}C NMR (75 MHz, $CDCl_3$) [rotamers mixture] δ 171.0, 162.7, 144.4, 143.1, 136.0, 135.8, 135.0, 132.9, 130.8, 130.5, 130.3, 128.9, 127.9, 127.1, 117.8, 48.6, 48.2, 32.9, 32.8, 25.5, 24.7, 17.8, 9.4. HRMS (ESI) calcd. for $C_{26}H_{28}Cl_3N_4O_2$ $[M+H]^+$: 533.1278 found: 533.1275.

(±) 5-(4-chlorophenyl)-*N*-(1-(cyclohexylamino)-3-methoxy-1-oxopropan-2-yl)-1-(2,4-di-chlorophenyl)-4-methyl-1*H*-pyrazole-3-carboxamide (21h): Yield: 52%; Mp: 175 – 177 ºC. ^{1}H NMR (300 MHz, DMSO-d_6) δ 9.38 (br s, 1H, NH), 7.53 (s, 1H, Ar-H), 7.39 – 7.20 (m, 4H, Ar-H), 7.13 (d, *J* = 8.4 Hz, 2H, Ar-H), 6.05 (br s, 1H, NH), 4.58 (d, *J* = 7.0 Hz, 1H, -NH*CH*(CH_2OCH_3)CO-), 3.83 – 3.68 (m, 1H, NH(*CH*-C_5H_{10})), 3.34 (d, *J* = 7.0 Hz, 2H, -NHCH(CH_2OCH_3)CO-), 3.23 (s, 3H, -NHCH(CH_2OCH_3)CO-),), 2.12 (s, 3H, Ar-CH_3), 1.98 – 1.73 (m, 2H, NH(CH-C_5H_{10})), 1.66 – 1.52 (m, 4H, NH(CH-C_5H_{10})), 1.39 – 0.99 (m, 4H, NH(CH-C_5H_{10})). HRMS (ESI) calcd. for $C_{27}H_{30}Cl_3N_4O_3$ $[M+H]^+$: 563.1305 found: 563.1309.

(±) 5-(4-chlorophenyl)-*N*-(2-(cyclohexylamino)-1-(4-hydroxyphenyl)-2-oxoethyl)-1-(2,4-dichlorophenyl)-4-methyl-1*H*-pyrazole-3-carboxamide (21i): Yield: 46%; Mp: 148 – 150ºC. ^{1}H NMR (300 MHz, $CDCl_3$) δ 8.12 (br d, *J* = 7.2 Hz, 1H, NH), 7.53-7.42 (m, 2H, Ar-H), 7.41 – 7.22 (m, 7H, Ar-H), 7.04 (d, *J* = 8.1 Hz, 2H, Ar-H), 5.79 (br d, *J* = 8.1 Hz, 1H, NH), 5.60 (d, *J* = 7.2 Hz, 1H, -NH*CH*(Ph)CO-), 3.85-3.65 (m, 1H, NH(*CH*-C_5H_{10})), 2.32 (s, 3H, Ar-CH_3), 1.99 – 1.72 (m, 2H, NH(CH-C_5H_{10})), 1.71 – 1.51 (m, 3H, NH(CH-C_5H_{10})), 1.42 – 1.22 (m, 2H, NH(CH-C_5H_{10})), 1.20 – 0.92 (m, 3H, NH(CH-C_5H_{10})). ^{13}C NMR (75 MHz, $CDCl_3$) δ 168.8, 162.2, 157.3, 144.5, 142.9, 138.3, 135.9, 135.8, 134.8, 132.8, 130.8, 130.6, 130.2, 128.9, 128.8, 128.2, 127.8, 127.5, 117.8, 57.0, 48.7, 32.8, 32.6, 25.4, 24.7, 24.6, 9.4. HRMS (ESI) calcd. for $C_{31}H_{30}Cl_3N_4O_3$ $[M+H]^+$: 611.1306 found: 611.1305.

(±) 5-(4-chlorophenyl)-*N*-(2-(cyclohexylamino)-1-(furan-2-yl)-2-oxoethyl)-1-(2,4-dichloro-phenyl)-4-methyl-1*H*-pyrazole-3-carboxamide (21j): Yield: 37%; Mp: 166 – 168ºC. ^{1}H NMR (300 MHz, $CDCl_3$) δ 7.98 (br d, J=7.5 Hz, 1H, NH), 7.42 – 7.36 (m, 2H, Ar-H), 7.31 – 7.25 (m, 5H, Ar-H), 7.15 (d, *J* = 5.0 Hz, 1H, Ar-H), 7.09 – 7.00 (m, 2H, Ar-H), 6.04 (br d, *J* = 8.1 Hz, 1H, NH), 5.77 (d, *J* = 7.6 Hz, 1H, -NH*CH*(2-furyl)CO-), 3.86 – 3.65 (m, 1H, NH(*CH*-C_5H_{10})), 2.34 – 2.33 (m, 3H, Ar-CH_3), 2.00 – 1.79 (m, 2H, NH(CH-C_5H_{10})), 1.75 – 1.52 (m, 3H, NH(CH-C_5H_{10})), 1.43 – 1.24 (m, 2H, NH(CH-C_5H_{10})), 1.23 – 1.01 (m, 3H, NH(CH-C_5H_{10})). ^{13}C NMR (75 MHz, $CDCl_3$) δ 168.5, 162.2, 144.4, 143.0, 138.5, 135.9, 134.9, 132.8, 130.8, 130.6, 130.2, 128.8, 127.8, 127.2, 126.7, 126.5, 123.5, 117.8, 52.6, 48.7, 32.8, 32.7, 25.4, 24.8, 24.7, 9.4. HRMS (ESI) calcd. for $C_{29}H_{28}Cl_3N_4O_3$ $[M+H]^+$: 585.1149 found: 585.1154.

(±) 5-(4-chlorophenyl)-*N*-(2-(cyclohexylamino)-2-oxo-1-(pyridin-2-yl)ethyl)-1-(2,4-dichlorophenyl)-4-methyl-1H-pyrazole-3-carboxamide (21k): Yield: 35%; Mp: 154 – 156ºC. ^{1}H NMR (300 MHz, $CDCl_3$) δ 8.71 (br s, 1H, NH), 8.44 (d, *J* = 7.2 Hz, 1H, Ar-H), 7.75 (t, *J* = 7.5 Hz, 1H, Ar-H), 7.57 (td, *J* = 7.3, 1.5 Hz, 1H, Ar-H), 7.49 (dd, *J* = 7.4, 1.6 Hz, 1H, Ar-H), 7.40 (d, *J* = 1.4 Hz, 1H, Ar-H), 7.33 – 7.23 (m, 4H, Ar-H), 7.04 (d, *J* = 8.5 Hz, 2H, Ar-H), 6.44 (br d, *J* = 8.0 Hz, 1H, NH), 5.69 (d, *J* = 7.2 Hz, 1H, -NH*CH*(2-pyridyl)CO-), 3.85 – 3.67 (m, 1H, NH(*CH*-C_5H_{10})), 2.31 (s, 3H, Ar-CH_3), 1.99 – 1.72 (m, 2H, NH(CH-C_5H_{10})), 1.71 – 1.44 (m, 3H, NH(CH-C_5H_{10})), 1.40 – 1.19 (m, 2H, NH(CH-C_5H_{10})), 1.19 – 0.95 (m, 3H, NH(CH-C_5H_{10})). ^{13}C NMR (75 MHz, $CDCl_3$) δ 167.9, 162.3, 157.81, 149.3, 149.1, 143.1, 137.3, 136.0, 135.8, 135.0, 134.9, 134.4, 132.8, 130.8, 130.6, 130.2, 128.9, 127.9, 123.8, 122.8, 121.5, 117.8, 54.6, 48.9, 32.8, 32.6, 25.4, 24.8, 24.7, 9.4. LRMS (CI) calcd. for $C_{30}H_{29}Cl_3N_5O_2$ $[M+H]^+$: 596.14 found: 596.13.

(±) 5-(4-chlorophenyl)-*N*-(2-(cyclohexylamino)-2-oxo-1-(pyridin-3-yl)ethyl)-1-(2,4-dichlorophenyl)-4-methyl-1H-pyrazole-3-carboxamide (21l): Yield: 35%; Mp: 148 – 150ºC. ^{1}H NMR (300 MHz, $CDCl_3$) δ 8.55 (s, 2H, Ar-H, NH), 8.20 (d, *J* = 7.4 Hz, 1H, Ar-H), 7.40 (d, *J* = 2.8 Hz, 3H, Ar-H), 7.35 – 7.23 (m, 4H, Ar-H), 7.04 (d, *J* = 8.1 Hz, 2H, Ar-H), 6.39 (br d, *J* = 8.1 Hz, 1H, NH), 5.68 (d, *J* = 7.4 Hz, 1H, -NH*CH*(3-pyridyl)CO-), 3.82 – 3.62 (m, 1H, NH(*CH*-C_5H_{10})), 2.31 (s, 3H, Ar-CH_3), 1.93 – 1.74 (m, 2H, NH(CH-C_5H_{10})), 1.73 – 1.50 (m, 3H, NH(CH-C_5H_{10})), 1.38 – 1.21 (m, 2H, NH(CH-C_5H_{10})), 1.19 – 0.96 (m, 3H, NH(CH-C_5H_{10})). ^{13}C NMR (75 MHz, $CDCl_3$) δ 167.4, 162.4, 150.2, 147.1, 144.0, 143.2, 136.0, 135.8, 135.0, 132.7, 130.8, 130.5, 130.3, 128.9, 127.9, 127.0, 122.2, 117.7, 55.9, 48.9, 32.7, 32.6, 25.3, 24.7, 24.7, 9.4. LRMS (CI) calcd. for $C_{30}H_{29}Cl_3N_5O_2$ $[M+H]^+$: 596.14 found: 596.14.

(±) 5-(4-chlorophenyl)-*N*-(2-(cyclohexylamino)-2-oxo-1-(pyridin-4-yl)ethyl)-1-(2,4-dichlorophenyl)-4-methyl-1H-pyrazole-3-carboxamide (21m): Yield: 42%; Mp: 156 – 157ºC. ^{1}H NMR (300 MHz, $CDCl_3$) δ 8.72 (br s, 0.5H, NH), 8.55 (br s, 0.5H, NH), 8.16 (d, *J* = 7.2 Hz, 1H, Ar-H), 7.82 (d, *J* = 6.8 Hz, 1H, Ar-H), 7.40 (s, 1H, Ar-H), 7.35 – 7.22 (m, 6H, Ar-H), 7.04 (d, *J* = 8.1 Hz, 2H, Ar-H), 6.32 (br s, 1H, NH), 5.67 (d, *J* = 7.2 Hz, 1H, -NH*CH*(4-pyridyl)CO-), 3.95 – 3.64 (m, 1H, NH(*CH*-C_5H_{10})), 2.31 (s, 3H, Ar-CH_3), 1.96-1.74 (m, 2H, NH(CH-C_5H_{10})), 1.73 – 1.50 (m, 3H, NH(CH-C_5H_{10})), 1.46 – 0.89 (m, 5H, NH(CH-C_5H_{10})). ^{13}C NMR (75 MHz, $CDCl_3$) δ 167.9, 162.3, 149.3, 149.1, 144.2, 143.1, 136.0, 135.9, 134.9, 132.8, 130.8, 130.6, 130.2, 128.9, 127.8, 127.1, 123.9, 123.8, 117.8, 54.7, 48.9, 32.8, 32.6, 25.4, 24.8, 24.7, 9.4. LRMS (CI) calcd. for $C_{30}H_{29}Cl_3N_5O_2$ $[M+H]^+$: 596.14 found: 596.13.

***N*-(tert-butyl)-2-(4-(5-(4-chlorophenyl)-1-(2,4-dichlorophenyl)-4-methyl-1H-pyrazole-3-carbonyl)piperazin-1-yl)acetamide (22a):** Yield: 53%; Mp: 167 – 169ºC. ^{1}H NMR (300 MHz, $CDCl_3$) δ 7.44 (d, *J* = 2.2 Hz, 1H, Ar-H), 7.33 – 7.22 (m, 3H, Ar-H), 7.16 (d, *J* = 8.5 Hz, 1H, Ar-H), 7.06 (d, *J* = 8.5 Hz, 2H, Ar-H), 6.96 (br s, 1H, NH), 3.93 – 3.74 (m, 4H, -CO(NC_4H_8N)-), 2.94 (s, 2H, -NCH_2CO-), 2.65 – 2.59 (m, 2H, -CO(NC_4H_8N)-), 2.58 – 2.50 (m, 2H, -CO(NC_4H_8N)-), 2.20 (s, 3H, Ar-CH_3), 1.36 (s, 9H, NHC(CH_3)$_3$).^{13}C NMR (75 MHz, $CDCl_3$) δ 168.6, 163.3, 146.1, 142.0, 135.9, 135.8, 134.9, 133.0, 130.6, 130.5, 130.3, 128.9, 127.9, 127.2, 117.1, 62.1, 53.8, 53.1, 50.6, 47.3, 42.1, 28.8, 9.1. HRMS (ESI) calcd. for $C_{27}H_{31}Cl_3N_5O_2$ $[M+H]^+$: 562.1538 found: 562.1535.

2-(4-(5-(4-chlorophenyl)-1-(2,4-dichlorophenyl)-4-methyl-1H-pyrazole-3-carbonyl)piperazin-1-yl)-*N*-cyclohexylacetamide (22b): Yield: 57%; Mp: 141 – 142ºC. ^{1}H NMR (300 MHz, $CDCl_3$) δ 7.44 (d, *J* = 2.2 Hz, 1H, Ar-H), 7.33 – 7.21 (m, 3H, Ar-H), 7.16 (d, *J* = 8.5 Hz, 1H, Ar-H), 7.06 (d, *J* = 8.5 Hz, 2H, Ar-H), 6.99 (br s, 1H, NH), 3.98 – 3.76 (m, 5H, -CO(NC_4H_8N)-, NH(*CH*-C_5H_{10})), 3.02 (s, 2H, -NCH_2CO-), 2.67 – 2.51 (m, 4H, -CO(NC_4H_8N)-), 2.20 (s, 3H, Ar-CH_3), 1.95 – 1.82 (m, 2H, NH(CH-C_5H_{10})), 1.75 – 1.57 (m, 3H, NH(CH-C_5H_{10})), 1.49 – 1.29 (m, 2H, NH(CH-C_5H_{10})), 1.28 – 1.11 (m, 3H, NH(CH-C_5H_{10})). ^{13}C NMR (75 MHz, $CDCl_3$) [rotamers mixture] δ 168.6, 168.5, 163.4, 163.3, 146.3, 146.1, 142.2, 142.0, 136.0, 135.9, 135.9, 135.8, 135.0, 134.9, 133.1, 133.0, 130.8, 130.6, 130.5, 130.3, 129.1, 128.9, 128.0, 127.9, 127.4, 127.2, 117.2, 117.0, 61.7, 61.5, 54.1, 53.9, 53.4, 53.2, 47.4, 47.3, 42.3, 42.1, 33.2, 33.1, 25.6, 25.5, 24.9, 24.7, 9.20, 9.0. HRMS (ESI) calcd. for $C_{29}H_{33}Cl_3N_5O_2$ $[M+H]^+$: 588.1694 found: 588.1688.

***N*-benzyl-2-(4-(5-(4-chlorophenyl)-1-(2,4-dichlorophenyl)-4-methyl-1H-pyrazole-3-carbonyl)-piperazin-1-yl)acetamide (22c):** Yield: 63%; Mp: 160 – 161ºC. ^{1}H NMR (300 MHz, $CDCl_3$) δ 7.44 (d, *J* = 2.2 Hz, 1H, Ar-H), 7.41 – 7.21 (m, 8H, Ar-H), 7.14 (d, *J* = 8.4 Hz, 1H, Ar-H), 7.10 – 7.01 (m, 2H, Ar-H), 4.49 (d, *J* = 6.0 Hz, 2H, NHCH_2Ph), 3.90 – 3.75 (m, 4H, -CO(NC_4H_8N)-), 3.11 (s, 2H, -NCH_2CO-), 2.69 – 2.53 (m, 4H, -CO(NC_4H_8N)-), 2.18 (s, 3H, Ar-CH_3). ^{13}C NMR (75 MHz, $CDCl_3$) δ 169.6, 163.3, 146.1, 142.0, 138.3, 135.9, 135.8, 134.9, 133.0, 130.7, 130.5, 130.3, 128.9, 128.8, 127.9, 127.6, 127.5, 127.2, 117.1, 61.5, 54.0, 53.3, 47.2, 43.0, 42.0, 9.0. LRMS (CI) calcd. for $C_{30}H_{29}Cl_3N_5O_2$ $[M+H]^+$: 596.14 found: 596.15.

***N*-((3s,5s,7s)-adamantan-1-yl)-2-(4-(5-(4-chlorophenyl)-1-(2,4-dichlorophenyl)-4-methyl-1H-pyrazole-3-carbonyl)piperazin-1-yl)acetamide (22d):** Yield: 57%; Mp: 149 – 150ºC. ^{1}HNMR (300 MHz, $CDCl_3$) δ 7.44 (d, *J* = 2.2 Hz, 1H, Ar-H), 7.34 – 7.22 (m, 3H, Ar-H), 7.16 (d, *J* = 8.4 Hz, 1H, Ar-H), 7.10 – 7.03 (m, 2H, Ar-H), 6.85 (br s, 1H, NH), 3.90 – 3.78 (m, 4H, -CO(NC_4H_8N)-), 2.93 (s, 2H, -NCH_2CO-), 2.66 – 2.59 (m, 2H, -CO(NC_4H_8N)-), 2.58 – 2.50 (m, 2H, -CO(NC_4H_8N)-), 2.20 (s, 3H, Ar-CH_3), 2.12 – 2.04 (m, 3H, NH($C_{10}H_{15}$)), 2.04 – 1.95 (m, 6H, NH($C_{10}H_{15}$)), 1.69 (s, 6H, NH($C_{10}H_{15}$)). ^{13}C NMR (75 MHz, $CDCl_3$) δ 168.4, 163.3, 146.1, 142.0, 135.9, 135.8, 134.9, 133.0, 130.6, 130.5, 130.3, 128.9, 127.9, 127.3, 117.0, 62.1, 53.8, 53.1, 51.2, 47.3, 42.1, 41.7, 36.3, 29.4, 9.0. HRMS (ESI) calcd. for $C_{33}H_{37}Cl_3N_5O_2$ $[M+H]^+$: 640.2007 found: 640.1998.

Ethyl (2-(4-(5-(4-chlorophenyl)-1-(2,4-dichlorophenyl)-4-methyl-1*H*-pyrazole-3-carbonyl)-piperazin-1-yl)acetyl)glycinate (22e): Yield: 61%; Mp: 175 – 176ºC. ^{1}HNMR (300 MHz, $CDCl_3$) δ 7.60 (br s, 1H, NH), 7.44 (d, *J* = 2.2 Hz, 1H, Ar-H), 7.34 – 7.23 (m, 3H, Ar-H), 7.16 (d, *J* = 8.5 Hz, 1H, Ar-H), 7.06 (d, *J* = 8.5 Hz, 2H, Ar-H), 4.12 – 3.98 (m, 4H, NHCH_2COO$CH_2$$CH_3$), 3.96 – 3.84 (m, 4H, -CO(NC_4H_8N)-), 3.11 (s, 2H, -NCH_2CO-), 2.80 – 2.58 (m, 4H, -CO(NC_4H_8N)-), 2.20 (s, 3H, Ar-CH_3), 1.21 (t, *J* = 7.1, 3H, $COOCH_2$$CH_3$). ^{13}C NMR (75 MHz, $CDCl_3$) δ 170.4, 170.1, 163.3, 146.1, 142.0, 135.9, 135.8, 134.9, 133.0, 130.6, 130.5, 130.3, 128.9, 127.8, 127.3, 117.0, 61.2, 61.0, 53.9, 53.2, 47.3, 42.1, 40.6, 14.2, 9.0. LRMS (CI) calcd. for $C_{27}H_{29}Cl_3N_5O_4$ $[M+H]^+$: 592.12 found: 592.12.

Diethyl ((2-(4-(5-(4-chlorophenyl)-1-(2,4-dichlorophenyl)-4-methyl-1H-pyrazole-3-carbonyl)piperazin-1-yl)acetamido)methyl)phosphonate (22f): Yield: 55%; Mp: 169 – 171ºC. ^{1}H NMR (300 MHz, $CDCl_3$) δ 7.43 (d, *J* = 2.2 Hz, 1H, Ar-H), 7.39 (br s, 1H, NH), 7.28 (d, *J* = 8.4 Hz, 2H, Ar-H), 7.23 (dd, *J* = 8.5 Hz, 2.3 Hz, 1H, Ar-H), 7.13 (d, *J* = 8.5 Hz, 1H, Ar-H), 7.05 (d, *J* = 8.5 Hz, 2H, Ar-H), 4.20 – 4.04 (m, 4H, PO(O$\mathit{CH_2}$$CH_3$)$_2$), 3.92 – 3.79 (m, 4H, -CO(N$\mathit{C_4H_8}$N)-), 3.72 (dd, *J* = 12.0, 6.1 Hz, 2H, NH$\mathit{CH_2}$PO(OCH_2CH_3)$_2$), 3.07 (s, 2H, -NCH_2CO-), 2.71 – 2.51 (m, 4H, -CO(N$\mathit{C_4H_8}$N)-), 2.18 (s, 3H, Ar-CH_3), 1.31 (t, *J* = 7.1 Hz, 6H, PO($OCH_2$$\mathit{CH_3}$)$_2$). ^{13}C NMR (75 MHz, $CDCl_3$) δ 169.7, 169.6, 163.3, 146.1, 142.0, 135.8, 135.8, 134.9, 132.9, 130.7, 130.5, 130.3, 129.0, 127.9, 127.2, 117.0, 62.6, 62.5, 61.2, 53.9, 53.2, 47.2, 42.0, 34.2, 16.4, 9.0. LRMS (CI) calcd. for $C_{28}H_{34}Cl_3N_5O_5P$ $[M+H]^+$: 656.14 found: 656.14.

***N*-(2-amino-2-oxoethyl)-2-(4-(5-(4-chlorophenyl)-1-(2,4-dichlorophenyl)-4-methyl-1*H*-pyrazole-3-carbonyl)piperazin-1-yl)acetamide (22g):** Yield: 38%; Mp: 105-106ºC. ^{1}H NMR (300 MHz, $CDCl_3$) δ 7.81 (t, *J* = 5.5 Hz, 1H, -CH_2CON*H*-), 7.40 (s, 1H, Ar-H), 7.33 – 7.11 (m, 4H, Ar-H), 7.14 – 6.98 (m, 2H, Ar-H), 6.45 (br s, 2H, -CH_2CON$\mathit{H_2}$), 3.95 (d, *J* = 5.4 Hz, 2H, -C$\mathit{H_2}$$CONH_2$), 3.85 (q, *J* = 4.5 Hz, 4H, -CO($NC_4$$\mathit{H_8}$N)-), 3.06 (s, 2H, -C$\mathit{H_2}$CONH-), 2.59 (dt, *J* = 14.0, 4.6 Hz, 4H, -CO($NC_4$$\mathit{H_8}$N)-), 2.15 (s, 3H, CH_3). ^{13}C NMR (75 MHz, $CDCl_3$) δ 171.3, 170.6, 163.3, 146.2, 142.1, 135.9, 135.7, 134.9, 132.9, 130.7, 130.6, 130.2, 128.9, 127.9, 127.2, 116.8, 54.0, 53.2, 47.3, 42.1, 9.0. LRMS (CI) calcd. For $C_{25}H_{26}Cl_3N_6O_3$ $[M+H]^+$: 563.10 found: 563.10.

2-(4-(5-(4-chlorophenyl)-1-(2,4-dichlorophenyl)-4-methyl-1H-pyrazole-3-carbonyl)pipe-razin 1-yl)-*N*-(2-morpholino-2-oxoethyl)acetamide (22h): Yield: 46%; Mp: 149 – 150ºC. ^{1}H NMR (300 MHz, $CDCl_3$) δ 8.05 (br s, 1H, NH), 7.45 (d, *J* = 2.2 Hz, 1H, Ar-H), 7.34 – 7.28 (m, 2H, Ar-H), 7.27 – 7.23 (m, 1H, Ar-H), 7.17 (d, *J* = 8.5 Hz, 1H, Ar-H), 7.11 – 7.05 (m, 2H, Ar-H), 4.11 (d, *J* = 4.5 Hz, 2H, NH$\mathit{CH_2}$CO(NC_4H_8O)), 4.02 – 3.83 (m, 4H, -CO(N$\mathit{C_4H_8}$N)-), 3.77 – 3.59 (m, 6H, $NHCH_2$CO(N$\mathit{C_4H_8}$O)), 3.50 – 3.39 (m, 2H, $NHCH_2$CO(N$\mathit{C_4H_8}$O)), 3.15 (s, 2H, -NCH_2CO-), 2.79 – 2.62 (m, 4H, -CO(N$\mathit{C_4H_8}$N)-), 2.20 (s, 3H, Ar-CH_3). ^{13}C NMR (75 MHz, $CDCl_3$) δ 169.8, 166.7, 163.3, 146.2, 135.9, 135.9, 135.8, 134.9, 133.0, 130.7, 130.5, 130.3, 128.9, 127.8, 127.3, 117.0, 66.7, 66.3, 61.2, 53.9, 53.3, 47.2, 44.9, 42.3, 42.1, 40.6, 9.0. LRMS (CI) calcd. for $C_{29}H_{32}Cl_3N_6O_4$ $[M+H]^+$: 633.15 found: 633.14.

***N*-(2-(4-acetylpiperazin-1-yl)-2-oxoethyl)-2-(4-(5-(4-chlorophenyl)-1-(2,4-dichlorophenyl) -4-methyl-1H-pyrazole-3-carbonyl)piperazin-1-yl)acetamide (22i):** Yield: 53%; Mp: 161 – 162ºC. ^{1}H NMR (300 MHz, $CDCl_3$) δ 7.99 (br s, 1H, NH), 7.44 (d, *J* = 2.2 Hz, 1H, Ar-H), 7.35 – 7.28 (m, 2H, Ar-H), 7.25 (dd, *J* = 9.0, 2.7 Hz, 1H, Ar-H), 7.17 (d, *J* = 8.5 Hz, 1H, Ar-H), 7.11 – 7.01 (m, 2H, Ar-H), 4.14 (d, *J* = 4.6 Hz, 2H, NH$\mathit{CH_2}$CO(NC_4H_8N)$COCH_3$), 3.98 – 3.84 (m, 4H, -CO(N$\mathit{C_4H_8}$N)CH_2-), 3.79 – 3.61 (m, 4H, CO(N$\mathit{C_4H_8}$N)$COCH_3$), 3.58 – 3.39 (m, 4H, CO(N$\mathit{C_4H_8}$N)$COCH_3$), 3.12 (s, 2H, -NCH_2CO-), 2.75 – 2.58 (m, 4H, -CO(N$\mathit{C_4H_8}$N)CH_2-), 2.20 (s, 3H, Ar-CH_3), 2.14 (s, 3H, CO(NC_4H_8N)CO$\mathit{CH_3}$). ^{13}C NMR (75 MHz, $CDCl_3$) δ 170.1, 163.3, 146.3, 142.0, 135.9, 135.8, 134.9, 133.0, 130.7, 130.5, 130.3, 128.9, 127.8, 127.3, 116.9, 61.3, 54.0, 53.3, 47.3, 45.8, 44.2, 42.2, 41.9, 41.1, 40.7, 21.3, 9.0. LRMS (CI) calcd. for $C_{31}H_{35}Cl_3N_7O_4$ $[M+H]^+$: 674.18 found: 674.17.

Ethyl 2-(1-((2-(4-(5-(4-chlorophenyl)-1-(2,4-dichlorophenyl)-4-methyl-1H-pyrazole-3-carbonyl)piperazin-1-yl)acetyl)glycyl)piperidin-4-yl)acetate (22j): Yield: 50%; Mp: 138 – 140ºC. 8.03 (br s, 1H, NH), 7.44 (d, *J* = 2.2 Hz, 1H, Ar-H), 7.35 – 7.29 (m, 2H, Ar-H), 7.28 – 7.21 (m, 1H, Ar-H), 7.17 (d, *J* = 8.5 Hz, 1H, Ar-H), 7.12 – 7.01 (m, 2H, Ar-H), 4.62 – 4.48 (m, 1H, -CO(NC_5H_9)CH_2CO-), 4.19 – 4.05 (m, 4H, -NHCH_2CO-, CO(NC_5H_9)CH_2COOCH_2CH_3), 4.00 – 3.84 (m, 4H, -CO(NC_4H_8N)-), 3.82 – 3.64 (m, 1H, -CO(NC_5H_9)CH_2CO-), 3.12 (s, 2H, -NCH_2CO-), 3.11 – 2.99 (m, 1H, -CO(NC_5H_9)CH_2CO-) 2.77 – 2.58 (m, 5H, -CO(NC_5H_9)CH_2CO-, CO(NC_4H_8N)-), 2.39 – 2.24 (m, 2H, -CO(NC_5H_9)CH_2CO-), 2.20 (s, 3H, Ar-CH_3), 2.14 – 1.95 (m, 1H, -CO(NC_5H_9)CH_2CO-) 1.90 – 1.73 (m, 2H, -CO(NC_5H_9)CH_2CO-), 1.49 – 1.09 (m, 5H, -CO(NC_5H_9)CH_2CO-, CO(NC_5H_9)CH_2COOCH_2CH_3). ^{13}C NMR (75 MHz, $CDCl_3$) [rotamers mixture] δ 172.0, 169.9, 166.1, 163.3, 146.3, 142.0, 136.0, 135.7, 134.8, 133.0, 132.5, 130.7, 130.6, 130.3, 128.9, 127.8, 127.4, 116.9, 68.2, 61.3, 60.5, 54.0, 53.3, 47.3, 44.5, 42.2, 40.7, 40.4, 32.9, 32.1, 31.4, 30.4, 28.9, 23.8, 23.0, 14.2, 9.0. LRMS (CI) calcd. for $C_{34}H_{40}Cl_3N_6O_5$ $[M+H]^+$: 717.21 found: 717.22.

2-(4-(5-(4-chlorophenyl)-1-(2,4-dichlorophenyl)-4-methyl-1H-pyrazole-3-carbonyl)piperazin-1-yl)-*N*-(2-morpholinoethyl)acetamide (22k): Yield: 48%; Mp: 152 – 153ºC. ^{1}H NMR (300 MHz, $CDCl_3$) δ 7.48 (br s, 1H, NH), 7.44 (d, *J* = 2.2 Hz, 1H, Ar-H), 7.33 – 7.20 (m, 3H, Ar-H), 7.14 (d, *J* = 8.5 Hz, 1H, Ar-H), 7.06 (d, *J* = 8.6 Hz, 2H, Ar-H), 3.95 – 3.81 (m, 4H, -CO(NC_4H_8N)-), 3.73 – 3.60 (m, 4H, CH_2(NC_4H_8O)), 3.46-3.33 (m, 2H, NHCH_2CH_2(NC_4H_8O)), 3.06 (s, 2H, -NCH_2CO-), 2.71 – 2.57 (m, 4H, -CO(NC_4H_8N)-), 2.54 – 2.42 (m, 6H, NHCH_2CH_2(NC_4H_8O)), 2.20 (s, 3H, Ar-CH_3). ^{13}C NMR (75 MHz, $CDCl_3$) δ 169.6, 163.2, 146.1, 142.1, 135.8, 135.8, 134.9, 132.9, 130.6, 130.5, 130.3, 128.9, 127.9, 127.2, 117.1, 67.0, 61.4, 57.1, 53.0, 53.3, 53.2, 47.4, 42.2, 35.1, 9.1. LRMS (CI) calcd. for $C_{29}H_{34}Cl_3N_6O_3$ $[M+H]^+$: 619.17 found: 619.18.

***N*-(tert-butyl)-2-(4-(5-(4-chlorophenyl)-1-(2,4-dichlorophenyl)-4-methyl-1H-pyrazole-3-carbonyl)piperazin-1-yl)-2-(pyridin-4-yl)acetamide (22l):** Yield: 46%; Mp: 163 – 164ºC. ^{1}H NMR (300 MHz, $CDCl_3$) δ 8.55 (br s, 1H, NH), 8.15 (d, *J* = 7.2 Hz, 1H, Ar-H), 7.81 (d, *J* = 6.8 Hz, 1H, Ar-H), 7.47 (d, *J* = 2.2 Hz, 1H, Ar-H), 7.43 – 7.38 (m, 2H, Ar-H), 7.35 – 7.22 (m, 3H, Ar-H), 7.19 – 7.12 (m, 1H, Ar-H), 7.11 – 7.05 (m, 2H, Ar-H), 4.25 (s, 1H, -N*CH*(4-pyridyl)CO-), 3.97 – 3.85 (m, 4H, -CO(NC_4H_8N)-), 3.53 – 3.32 (m, 2H, -CO(NC_4H_8N)-), 3.30 – 3.12 (m, 2H, -CO(NC_4H_8N)-), 2.30 (s, 3H, Ar-CH_3), 1.27 (s, 9H, NHC(CH_3)$_3$). ^{13}C NMR (75 MHz, $CDCl_3$) δ 169.1, 162.1, 150.0, 149.9, 142.7, 141.4, 137.9, 135.9, 134.6, 134.5, 131.3, 130.2, 129.9, 129.9, 129.6, 129.2, 127.8, 126.2, 124.9, 124.9, 111.1, 69.2, 52.2, 48.8, 48.8, 45.5, 45.5, 28.5, 9.3. LRMS (CI) calcd. for $C_{32}H_{34}Cl_3N_6O_2$ [M+H]$^+$: 639.18 found: 639.19.

2-(4-(5-(4-chlorophenyl)-1-(2,4-dichlorophenyl)-4-methyl-1H-pyrazole-3-carbonyl)pipe-razin-1-yl)-*N*-cyclohexyl-2-(pyridin-4-yl)acetamide (22m): Yield: 37%; Mp: 145 – 146ºC. ^{1}H NMR (300 MHz, $CDCl_3$) δ 8.54 (br s, 1H, NH), 8.16 (d, *J* = 7.2 Hz, 1H, Ar-H), 7.80 (d, *J* = 6.8 Hz, 1H, Ar-H),7.46 (d, *J* = 2.2 Hz, 1H, Ar-H), 7.41 – 7.36 (m, 2H, Ar-H), 7.35 – 7.22 (m, 3H, Ar-H), 7.18 – 7.12 (m, 1H, Ar-H), 7.11 – 7.05 (m, 2H, Ar-H), 4.24 (s, 1H, -N*CH*(4-pyridyl)CO-), 3.97 – 3.70 (m, 5H, -CO(NC_4H_8N)-, NH(*CH*-C_5H_{10})), 3.52 – 3.32 (m, 2H, -CO(NC_4H_8N)-), 3.29 – 3.12 (m, 2H, -CO(NC_4H_8N)-), 2.31 (s, 3H, Ar-CH_3), 1.99 – 1.72 (m, 2H, NH(CH-C_5H_{10})), 1.71 – 1.44 (m, 3H, NH(CH-C_5H_{10})), 1.40 – 1.19 (m, 2H, NH(CH-C_5H_{10})), 1.19 – 0.95 (m, 3H, NH(CH-C_5H_{10})). ^{13}C NMR (75 MHz, $CDCl_3$) δ 169.1, 162.1, 150.0, 149.9, 142.7, 141.4, 137.9, 135.9, 134.6, 134.5, 131.3, 130.2, 129.9, 129.9, 129.6, 129.2, 127.8, 126.2, 124.9, 124.9, 111.1, 69.2, 48.9, 48.8, 48.8, 45.5, 45.5, 32.8, 32.6, 25.4, 24.8, 26.7, 9.3. LRMS (CI) calcd. for $C_{34}H_{36}Cl_3N_6O_2$ [M+H]$^+$: 665.20 found: 665.19.

***N*-benzyl-2-(4-(5-(4-chlorophenyl)-1-(2,4-dichlorophenyl)-4-methyl-1H-pyrazole-3-carbonyl)piperazin-1-yl)-2-(pyridin-4-yl)acetamide (22n):** Yield: 41%; Mp: 159 – 160 ºC. ^{1}H NMR (300 MHz, $CDCl_3$) δ 8.51 (br s, 1H, NH), 8.15 (d, *J* = 7.2 Hz, 1H, Ar-H), 7.81 (d, *J* = 6.8 Hz, 1H, Ar-H), 7.46 (d, *J* = 2.2 Hz, 1H, Ar-H), 7.42 – 7.20 (m, 10H, Ar-H), 7.18 – 7.12 (m, 1H, Ar-H), 7.11 – 7.05 (m, 2H, Ar-H), 4.49 (d, *J* = 6.0 Hz, 2H, NHCH_2Ph), 4.25 (s, 1H, -NH*CH*(4-pyridyl)CO-), 3.99 – 3.88 (m, 4H, -CO(NC_4H_8N)-), 3.53 – 3.32 (m, 2H, -CO(NC_4H_8N)-), 3.29 – 3.10 (m, 2H, -CO(NC_4H_8N)-), 2.32 (s, 3H, Ar-CH_3), ^{13}C NMR (75 MHz, $CDCl_3$) δ 169.1, 162.1, 150.0, 149.9, 142.7, 141.4, 138.9, 137.9, 135.9, 134.6, 134.5, 131.3, 130.2, 129.9, 129.9, 129.6, 129.2, 128.5, 128.5, 127.8, 127.5, 127.4, 127.4, 126.2, 124.9, 124.9, 111.1, 69.2, 48.8, 48.8, 47.2, 45.5, 45.5, 9.3. LRMS (CI) calcd. for $C_{35}H_{32}Cl_3N_6O_2$ $[M+H]^+$: 673.16 found: 673.16.

***N*-(tert-butyl)-2-(4-(5-(4-chlorophenyl)-1-(2,4-dichlorophenyl)-4-methyl-1H-pyrazole-3-carbonyl)-1,4-diazepan-1-yl)acetamide (23a):** Yield: 49%; Mp: 145 – 146ºC. ^{1}H NMR (300 MHz, $CDCl_3$) [rotamers mixture] δ 7.45 (s, 1H, Ar-H), 7.34 – 7.20 (m, 3H, Ar-H), 7.16 – 7.01 (m, 3H, Ar-H), 3.96 – 3.65 (m, 4H, -CO(NC_5H_{10}N)-), 3.06 (s, 1H, -NCH_2CO-), 3.03 (s, 1H, -NCH_2CO-), 2.96 – 2.82 (m, 2H, -CO(NC_5H_{10}N)-), 2.80 – 2.67 (m, 2H, -CO(NC_5H_{10}N)-), 2.20 (s, 3H, Ar-CH_3), 2.03 – 1.88 (m, 2H, -CO(NC_5H_{10}N)-), 1.40 – 1.25 (m, 9H, NHC(CH_3)$_3$). ^{13}C NMR (75 MHz, $CDCl_3$) [rotamers mixture] δ 169.5, 164.7, 146.9, 146.8, 141.8, 141.8, 136.0, 135.9, 135.7, 135.7, 134.8, 133.0, 130.6, 130.5, 130.3, 130.3, 128.9, 127.9, 127.8, 127.4, 127.3, 116.8, 116.6, 62.2, 62.0, 57.7, 55.6, 55.2, 54.9, 50.6, 50.5, 49.4, 48.6, 46.4, 45.4, 29.3, 28.8, 27.1, 9.1. LRMS (CI) calcd. for $C_{28}H_{33}Cl_3N_5O_2$ $[M+H]^+$: 576.17 found: 576.16.

2-(4-(5-(4-chlorophenyl)-1-(2,4-dichlorophenyl)-4-methyl-1H-pyrazole-3-carbonyl)-1,4-diazepan-1-yl)-*N*-cyclohexylacetamide (23b): Yield: 53%; Mp: 136 – 137ºC. ^{1}H NMR (300 MHz, $CDCl_3$) [rotamers mixture] δ 7.47 – 7.42 (m, 1H, Ar-H), 7.34 – 7.17 (m, 3H, Ar-H), 7.16 – 7.00 (m, 4H, Ar-H), 3.92 – 3.66 (m, 5H, -CO(NC_5H_{10}N)-, NH(*CH*-C_5H_{10})), 3.13 (s, 1H, -NCH_2CO-), 3.10 (s, 1H, -NCH_2CO-), 2.93 – 2.79 (m, 2H, -CO(NC_5H_{10}N)-), 2.79 – 2.66 (m, 2H, -CO(NC_5H_{10}N)-), 2.19 (s, 3H, Ar-CH_3), 2.04 – 1.78 (m, 4H, -CO(NC_5H_{10}N)-, NH(CH-C_5H_{10})), 1.72 – 1.53 (m, 2H, NH(CH-C_5H_{10})), 1.44 – 1.01 (m, 6H, NH(CH-C_5H_{10})). ^{13}C NMR (75 MHz, $CDCl_3$) [rotamers mixture] δ 169.3, 164.8, 164.7, 146.9, 146.8, 141.8, 141.7, 136.0, 135.9, 135.7, 135.7, 134.8, 133.1, 133.0, 130.6, 130.4, 130.3, 130.3, 128.9, 127.8, 127.3, 127.3, 116.8, 116.5, 61.5, 61.3, 57.8, 55.6, 55.1, 55.0, 49.3, 48.7, 47.5, 47.4, 46.3, 45.5, 33.1, 29.4, 27.0, 25.4, 24.8, 24.7, 9.2, 9.1. LRMS (CI) calcd. for $C_{30}H_{35}Cl_3N_5O_2$ $[M+H]^+$: 602.18 found: 602.19.

***N*-benzyl-2-(4-(5-(4-chlorophenyl)-1-(2,4-dichlorophenyl)-4-methyl-1H-pyrazole-3-carbonyl)-1,4-diazepan-1-yl)acetamide (23c):** Yield: 49%; Mp: 143 – 144 ºC. ^{1}H NMR (300 MHz, $CDCl_3$) [rotamers mixture] δ 7.58 (br s, 0.5H, NH), 7.50 (br s, 0.5H, NH), 7.44 (d, J = 2.2 Hz, 0.5H, Ar-H), 7.41 (d, J = 2.3 Hz, 0.5H, Ar-H), 7.35 – 7.17 (m, 8H, Ar-H), 7.09 (d, J = 8.9 Hz, 1H, Ar-H), 7.04 (d, J = 8.5 Hz, 2H, Ar-H), 4.75 – 4.37 (m, 2H, NHCH_2Ph), 3.89 – 3.64 (m, 4H, -CO(NC_5H_{10}N)-), 3.24 (s, 1H, -NCH_2CO-), 3.19 (s, 1H, -NCH_2CO-), 2.99 – 2.80 (m, 2H, -CO(NC_5H_{10}N)-), 2.81 – 2.66 (m, 2H, -CO(NC_5H_{10}N)-), 2.19 – 2.15 (m, 3H, Ar-CH_3), 2.02 – 1.88 (m, 2H, -CO(NC_5H_{10}N)-,). ^{13}C NMR (75 MHz, $CDCl_3$) [rotamers mixture] δ 170.5, 170.4, 164.9, 164.7, 146.9, 146.8, 141.8, 141.8, 138.4, 138.4, 135.9, 135.9, 135.7, 135.7, 134.8, 133.0, 130.6, 130.4, 130.3, 130.3, 128.9, 128.7, 128.6, 127.9, 127.8, 127.5, 127.3, 127.3, 116.8, 116.5, 61.2, 60.9, 58.0, 55.6, 55.3, 55.0, 49.0, 48.6, 46.1, 45.4, 42.9, 28.9, 26.7, 9.1, 9.0. LRMS (CI) calcd. for $C_{31}H_{31}Cl_3N_5O_2$ $[M+H]^+$: 610.15 found: 610.16.

***N*-((3s,5s,7s)-adamantan-1-yl)-2-(4-(5-(4-chlorophenyl)-1-(2,4-dichlorophenyl)-4-methyl-1H-pyrazole-3-carbonyl)-1,4-diazepan-1-yl)acetamide (23d):** Yield: 44%; Mp: 166 – 167ºC. ^{1}H NMR (300 MHz, $CDCl_3$) [rotamers mixture] δ 7.44 (d, *J* = 2.3 Hz, 1H, Ar-H), 7.28 (d, *J* = 8.5 Hz, 2H, Ar-H), 7.24 – 7.19 (m, 1H, Ar-H), 7.11 (d, *J* = 7.4 Hz, 1H, Ar-H), 7.05 (d, *J* = 7.5 Hz, 2H, Ar-H), 6.94 (br s, 1H, NH), 3.89 – 3.74 (m, 4H, , -CO(NC_5H_{10}N)-), 3.04 (s, 1H, -NCH_2CO-), 3.00 (s, 1H, -NCH_2CO-), 2.92 – 2.78 (m, 2H, -CO(NC_5H_{10}N)-), 2.79 – 2.67 (m, 2H, -CO(NC_5H_{10}N)-), 2.19 (s, 3H, Ar-CH_3), 2.07 – 1.99 (m, 5H, -CO(NC_5H_{10}N)-, NH($C_{10}H_{15}$)), 1.99 – 1.88 (m, 6H, NH($C_{10}H_{15}$)), 1.64 (s, 6H, NH($C_{10}H_{15}$)). ^{13}C NMR (75 MHz, $CDCl_3$) [rotamers mixture] δ 169.3, 164.7, 164.7, 146.9, 146.8, 141.8, 141.7, 136.0, 135.9, 135.7, 135.6, 134.8, 134.8, 133.0, 130.6, 130.4, 130.4, 130.3, 128.9, 127.9, 127.8, 127.4, 127.3, 116.8, 116.6, 62.3, 62.3, 57.7, 55.6, 55.2, 54.9, 51.2, 51.1, 49.6, 48.6, 46.5, 45.4, 41.6, 36.3, 29.4, 29.4, 29.3, 27.2, 9.2, 9.1. LRMS (CI) calcd. for $C_{34}H_{39}Cl_3N_5O_2$ $[M+H]^+$: 654.22 found: 654.23.

Ethyl (2-(4-(5-(4-chlorophenyl)-1-(2,4-dichlorophenyl)-4-methyl-1*H*-pyrazole-3-carbonyl)-1,4-diazepan-1-yl)acetyl)glycinate (23e): Yield: 61%; Mp: 175 – 176ºC. ^{1}H NMR (300 MHz, $CDCl_3$) δ 7.80 (br s, 1H, NH), 7.44 (d, *J* = 2.2 Hz, 1H, Ar-H), 7.35 – 7.22 (m, 3H, Ar-H), 7.16 (d, *J* = 8.4 Hz, 1H, Ar-H), 7.12 (d, *J* = 8.5 Hz, 2H, Ar-H), 4.12 – 3.98 (m, 4H, NHCH_2COO$CH_2$$CH_3$), 3.96 – 3.84 (m, 6H, -CO(NC_5H_{10}N)-), 3.28 (s, 2H, -NCH_2CO-), 2.80 – 2.58 (m, 2H, -CO(NC_5H_{10}N)-), 2.20 (s, 3H, Ar-CH_3), 1.52 – 1.68 (m, 2H, -CO(NC_5H_{10}N)-), 1.22 (t, *J* = 7.1, 3H, COO$CH_2$$CH_3$). LRMS (CI) calcd. for $C_{28}H_{31}Cl_3N_5O_4$ $[M+H]^+$: 606.14 found: 606.12.

Diethyl ((2-(4-(5-(4-chlorophenyl)-1-(2,4-dichlorophenyl)-4-methyl-1H-pyrazole-3-carbonyl)-1,4-diazepan-1-yl)acetamido)methyl)phosphonate (23f): Yield: 32%; Mp: 175 – 176ºC. ^{1}H NMR (300 MHz, $CDCl_3$) [rotamers mixture] δ 7.48 (br s, 1H, NH), 7.44 (d, *J* = 2.2 Hz, 1H, Ar-H), 7.29 (d, *J* = 8.4 Hz, 2H, Ar-H), 7.26 – 7.20 (m, 1H, Ar-H), 7.16 – 7.09 (m, 1H, Ar-H), 7.05 (d, *J* = 7.9 Hz, 2H, Ar-H), 4.19 – 4.05 (m, 4H, PO(O$\mathit{CH_2}$$CH_3$)$_2$), 3.94 – 3.78 (m, 4H, -CO(N$\mathit{C_5H_{10}}$N)-), 3.75 – 3.61 (m, 2H, NH$\mathit{CH_2}$PO(OCH_2CH_3)$_2$), 3.21 (s, 1H, -NCH_2CO-), 3.17 (s, 1H, -NCH_2CO-), 2.95 – 2.82 (m, 2H, -CO(N$\mathit{C_5H_{10}}$N)-), 2.82 – 2.65 (m, 2H, -CO(N$\mathit{C_5H_{10}}$N)-), 2.20 (s, 3H, Ar-CH_3), 2.09 – 1.91 (m, 2H, -CO(N$\mathit{C_5H_{10}}$N)-), 1.31 (t, *J* = 7.0 Hz, 6H, PO($OCH_2$$\mathit{CH_3}$)$_2$). ^{13}C NMR (75 MHz, $CDCl_3$) [rotamers mixture] δ 170.4, 164.8, 164.7, 146.8, 146.7, 141.8, 141.8, 135.9, 135.9, 135.9, 135.7, 134.8, 133.0, 132.9, 130.6, 130.5, 130.5, 130.3, 130.2, 128.9, 127.9, 127.3, 116.8, 116.6, 62.6, 62.5, 61.2, 61.0, 58.3, 55.7, 55.5, 55.0, 49.2, 48.3, 46.3, 45.5, 34.2, 28.8, 26.6, 16.4, 16.4, 9.1, 9.0. LRMS (CI) calcd. for $C_{29}H_{36}Cl_3N_5O_5P$ $[M+H]^+$: 670.15 found: 670.15.

***N*-(2-amino-2-oxoethyl)-2-(4-(5-(4-chlorophenyl)-1-(2,4-dichlorophenyl)-4-methyl-1*H*-pyrazole-3-carbonyl)-1,4-diazepan-1-yl)acetamide (23g):** Yield: 44%; Mp: 108-110ºC. ^{1}H NMR (300 MHz, $CDCl_3$) δ 7.86 (t, *J* = 5.6 Hz, 1H, -CH_2CON*H*-), 7.46 (s, 1H, Ar-H), 7.39 – 6.96 (m, 6H, Ar-H), 6.03 (s, 2H, -CH_2CON$\mathit{H_2}$), 3.95 (d, *J* = 5.7 Hz, 2H, -C$\mathit{H_2}$$CONH_2$), 3.93 – 3.78 (m, 4H, -CO($NC_5H_{10}N$)-), 3.24 (s, 2H, -C$\mathit{H_2}$CONH-), 2.92 (dt, *J* = 15.2, 5.6 Hz, 2H, -CO($NC_5H_{10}N$)-), 2.81 (q, *J* = 5.9 Hz, 2H, -CO($NC_5H_{10}N$)-), 2.21 (d, *J* = 1.6 Hz, 3H, CH_3), 2.00 (dp, *J* = 29.6, 6.1 Hz, 2H, -CO($NC_5H_{10}N$)-). ^{13}C NMR (75 MHz, $CDCl_3$) δ 172.0, 170.5, 163.4, 146.2, 142.0, 135.9, 135.7, 134.9, 133.0, 130.8, 130.4, 130.2, 128.9, 128.0, 127.1, 116.1, 55.6, 54.2, 46.3, 42.2, 26.6, 9.1. LRMS (CI) calcd. for $C_{26}H_{28}Cl_3N_6O_3$ $[M+H]^+$: 577.12 found 577.10.

2-(4-(5-(4-chlorophenyl)-1-(2,4-dichlorophenyl)-4-methyl-1H-pyrazole-3-carbonyl)-1,4-diazepan-1-yl)-*N*-(2-morpholino-2-oxoethyl)acetamide (23h): Yield: 33%; Mp: 166 – 167ºC. ^{1}H NMR (300 MHz, $CDCl_3$) δ 8.17 (br s, 1H, NH), 7.45 (d, *J* = 2.4 Hz, 1H, Ar-H), 7.34 – 7.21 (m, 3H, Ar-H), 7.14 (d, *J* = 9.0 Hz, 1H, Ar-H), 7.12 – 6.99 (m, 2H, Ar-H), 4.13 – 4.03 (m, 2H, NH$\mathit{CH_2}$CO(NC_4H_8O)), 4.01 – 3.83 (m, 4H, -CO(N$\mathit{C_5H_{10}}$N)-), 3.80 – 3.59 (m, 6H, NHCH_2CO(N$\mathit{C_4H_8}$O)), 3.58 – 3.37 (m, 2H, NHCH_2CO(N$\mathit{C_4H_8}$O)), 3.34 – 3.20 (m, 2H, -NCH_2CO-), 3.11 – 2.73 (m, 4H, -CO(N$\mathit{C_5H_{10}}$N)-), 2.21 (s, 3H, Ar-CH_3), 2.18 – 2.00 (m, 2H, -CO(N$\mathit{C_5H_{10}}$N)-). ^{13}C NMR (75 MHz, $CDCl_3$) [rotamers mixture] δ 171.2, 170.1, 161.3, 144.5, 136.9, 135.9, 134.6, 134.4, 131.7, 130.6, 130.1, 129.2, 128.0, 126.1, 117.0, 66.4, 66.4, 57.1, 54.5, 54.0, 52.3, 52.0, 46.7, 46.7, 46.6, 45.0, 44.7, 44.6, 42.8, 42.5, 26.3, 26.1, 25.9, 9.2, 9.1. LRMS (CI) calcd. for $C_{30}H_{34}Cl_3N_6O_4$ $[M+H]^+$: 647.17 found: 647.17.

***N*-(2-(4-acetylpiperazin-1-yl)-2-oxoethyl)-2-(4-(5-(4-chlorophenyl)-1-(2,4-dichlorophenyl) -4-methyl-1H-pyrazole-3-carbonyl)-1,4-diazepan-1-yl)acetamide (23i):** Yield: 41%; Mp: 191 – 192ºC. ^{1}H NMR (300 MHz, $CDCl_3$) δ 8.08 (br s, 1H, NH), 7.45 (t, *J* = 2.0 Hz, 1H, Ar-H), 7.34 – 7.28 (m, 2H, Ar-H), 7.27 – 7.20 (m, 1H, Ar-H), 7.16 (d, *J* = 8.3 Hz, 1H, Ar-H), 7.10 – 7.04 (m, 2H, Ar-H), 4.21 – 4.07 (m, 2H, NH$\mathit{CH_2}$CO(NC_4H_8N)$COCH_3$), 3.95 – 3.80 (m, 4H, -CO(N$\mathit{C_5H_{10}}$N)CH_2-), 3.74 – 3.59 (m, 4H, CO(N$\mathit{C_4H_8}$N)$COCH_3$), 3.56 – 3.32 (m, 4H, CO(N$\mathit{C_4H_8}$N)$COCH_3$), 3.25 (s, 1H, -N$\mathit{CH_2}$CO-), 3.21 (s, 1H, -N$\mathit{CH_2}$CO-), 3.05 – 2.64 (m, 4H, -CO(N$\mathit{C_5H_{10}}$N) CH_2-), 2.21 (s, 3H, Ar-CH_3), 2.14 (s, 3H, CO(NC_4H_8N)CO$\mathit{CH_3}$), 2.11 – 2.04 (m, 2H, -CO(N$\mathit{C_5H_{10}}$N)CH_2-). ^{13}C NMR (75 MHz, $CDCl_3$) [rotamers mixture] δ 171.0, 164.8, 164.8, 147.0, 141.8, 136.0, 135.7, 134.8, 133.0, 130.7, 130.5, 130.3, 130.3, 128.9, 127.8, 127.4, 116.6, 116.5, 61.4, 61.2, 58.6, 55.7, 55.2, 49.6, 48.4, 46.6, 45.8, 45.6, 44.5, 44.2, 41.9, 41.1, 40.7, 28.8, 26.7, 21.3, 9.1, 9.0. LRMS (CI) calcd. for $C_{32}H_{37}Cl_3N_7O_4$ $[M+H]^+$: 688.20 found: 688.19.

Ethyl 2-(1-((2-(4-(5-(4-chlorophenyl)-1-(2,4-dichlorophenyl)-4-methyl-1H-pyrazole-3-carbonyl)-1,4-diazepan-1-yl)acetyl)glycyl)piperidin-4-yl)acetate (23j): Yield: 38%; Mp: 188 – 190ºC. ^{1}H NMR (300 MHz, $CDCl_3$) δ 8.13 (brs, 1H, NH), 7.44 (t, *J* = 2.1 Hz, 1H, Ar-H), 7.34 – 7.28 (m, 2H, Ar-H), 7.29 – 7.19 (m, 1H, Ar-H), 7.16 (d, *J* = 7.4 Hz, 1H, Ar-H), 7.10 – 7.05 (m, 2H, Ar-H), 4.62 – 4.47 (m, 1H, -CO(N$\mathit{C_5H_9}$)CH_2CO-), 4.20 – 4.00 (m, 4H, -NH$\mathit{CH_2}$CO-, CO(NC_5H_9)$CH_2COO\mathit{CH_2}CH_3$), 3.96 – 3.81 (m, 4H, -CO(N$\mathit{C_5H_{10}}$N)-), 3.80 – 3.60 (m, 1H, CO(N$\mathit{C_5H_9}$)CH_2CO-), 3.25 (s, 1H, -N$\mathit{CH_2}$CO-), 3.21 (s, 1H, -N$\mathit{CH_2}$CO-), 3.14 – 2.97 (m, 1H, -CO(N$\mathit{C_5H_9}$)CH_2CO-), 2.97 – 2.74 (m, 5H, -CO(N$\mathit{C_5H_{10}}$N)-, -CO(N$\mathit{C_5H_9}$)CH_2CO-) 2.33 – 2.21 (m, 2H, -CO(NC_5H_9)$\mathit{CH_2}$CO-), 2.21 (s, 3H, Ar-CH_3), 2.15 – 1.95 (m, 3H, -CO(N$\mathit{C_5H_9}$)CH_2CO-, -CO(N$\mathit{C_5H_{10}}$N)-), 1.90 – 1.71 (m, 2H, CO(N$\mathit{C_5H_9}$)CH_2CO-), 1.35 – 1.14 (m, 5H, -CO(N$\mathit{C_5H_9}$)CH_2CO-, CO(N$\mathit{C_5H_9}$)$CH_2COOCH_2\mathit{CH_3}$). ^{13}C NMR (75 MHz, $CDCl_3$) [rotamers mixture] δ 172.0, 170.7, 166.1, 164.8, 146.9, 141.8, 135.6, 134.8, 133.0, 130.7, 130.5, 130.3, 128.9, 127.8, 116.8, 61.4, 61.2, 60.5, 58.6, 56.2, 55.6, 55.0, 51.6, 49.5, 48.4, 46.5, 45.5, 44.5, 42.2, 40.7, 40.4, 32.9, 32.0, 31.4, 28.6, 26.6, 14.2, 9.1. LRMS (CI) calcd. for $C_{35}H_{42}Cl_3N_6O_5$ $[M+H]^+$: 731.23 found: 731.23.

2-(4-(5-(4-chlorophenyl)-1-(2,4-dichlorophenyl)-4-methyl-1H-pyrazole-3-carbonyl)-1,4-diazepan-1-yl)-*N*-(2-morpholinoethyl)acetamide (23k): Yield: 31%; Mp: 143 – 144 ºC. ^{1}H NMR (300 MHz, $CDCl_3$) [rotamers mixture] δ 7.55 (brs, 1H, NH), 7.45 (s, 1H, Ar-H), 7.34 – 7.19 (m, 3H, Ar-H), 7.18 – 7.02 (m, 3H, Ar-H), 3.93 – 3.79 (m, 4H, -CO(NC_5H_{10}N)-), 3.75 – 3.59 (m, 4H, CH_2(NC_4H_8O)), 3.37 (t, *J* = 6.7 Hz, 2H, NH$CH_2$$CH_2$($NC_4H_8O$)), 3.19 (s, 1H, -N$CH_2$CO-), 3.14 (s, 1H, -N$CH_2$CO-), 2.97 – 2.82 (m, 2H, -CO(NC_5H_{10}N)-), 2.82 – 2.68 (m, 2H, -CO(NC_5H_{10}N)-), 2.57 – 2.36 (m, 6H, NH$CH_2$$CH_2$(N$C_4H_8$O)), 2.20 (s, 3H), 2.08 – 1.95 (m, 2H, -CO(NC_5H_{10}N)-). ^{13}C NMR (75 MHz, $CDCl_3$) [rotamers mixture] δ 170.5, 164.9, 164.7, 146.9, 146.8, 141.8, 141.8, 135.9, 135.7, 134.8, 133.0, 132.9, 130.6, 130.5, 130.4, 130.3, 128.9, 127.9, 127.3, 116.8, 116.5, 70.5, 66.9, 61.8, 61.5, 58.2, 57.3, 57.2, 55.6, 55.4, 53.3, 49.5, 48.4, 46.7, 45.6, 35.2, 35.1, 29.6, 29.4, 26.9, 9.1, 9.1. LRMS (CI) calcd. for $C_{30}H_{36}Cl_3N_6O_3$ $[M+H]^+$: 633.19 found: 633.20.

***N*-(tert-butyl)-2-(4-(5-(4-chlorophenyl)-1-(2,4-dichlorophenyl)-4-methyl-1H-pyrazole-3-carbonyl)-1,4-diazepan-1 (23l):** Yield: 30%; Mp: 178 – 179ºC. ^{1}H NMR (300 MHz, $CDCl_3$) δ 8.53 (brs, 1H, NH), 8.16 (d, *J* = 7.2 Hz, 1H, Ar-H), 7.82 (d, *J* = 6.8 Hz, 1H, Ar-H), 7.46 (d, *J* = 2.2 Hz, 1H, Ar-H), 7.41 – 7.36 (m, 2H, Ar-H), 7.35 – 7.22 (m, 3H, Ar-H), 7.19 – 7.12 (m, 1H, Ar-H), 7.11 – 7.05 (m, 2H, Ar-H), 4.27 (s, 1H, -N*CH*(4-pyridyl)CO-), 3.96 – 3.65 (m, 4H, -CO(NC_5H_{10}N)-), 2.96 – 2.82 (m, 2H, -CO(NC_5H_{10}N)-), 2.80 – 2.67 (m, 2H, -CO(NC_5H_{10}N)-), 2.31 (s, 3H, Ar-CH_3), 2.03 – 1.88 (m, 2H, -CO(NC_5H_{10}N)-), 1.40 – 1.25 (m, 9H, NHC(CH_3)$_3$). ^{13}C NMR (75 MHz, $CDCl_3$) δ 169.1, 164.1, 150.0, 149.9, 142.7, 141.4, 137.9, 135.9, 134.6, 134.5, 131.3, 130.2, 129.9, 129.9, 129.6, 129.2, 127.8, 126.2, 124.9, 124.9, 116.1, 69.3, 54.4, 50.6, 50.4, 47.0, 44.7, 28.5, 26.3, 9.1. LRMS (CI) calcd. for $C_{33}H_{36}Cl_3N_6O_2$ $[M+H]^+$: 653.20 found: 653.21.

2-(4-(5-(4-chlorophenyl)-1-(2,4-dichlorophenyl)-4-methyl-1H-pyrazole-3-carbonyl)-1,4-diazepan-1-yl)-*N*-cyclohexyl-2-(pyridin-4-yl)acetamide (23m): Yield: 35%; Mp: 189 – 190ºC. ^{1}H NMR (300 MHz, $CDCl_3$) δ 8.54 (brs, 1H, NH), 8.15 (d, *J* = 7.2 Hz, 1H, Ar-H), 7.80 (d, *J* = 6.8 Hz, 1H, Ar-H), 7.46 (d, *J* = 2.2 Hz, 1H, Ar-H), 7.41 – 7.36 (m, 2H, Ar-H), 7.35 – 7.22 (m, 3H, Ar-H), 7.19 – 7.12 (m, 1H, Ar-H), 7.11 – 7.05 (m, 2H, Ar-H), 4.26 (s, 1H, -N*CH*(4-pyridyl)CO-), 3.96 – 3.65 (m, 5H, -CO(NC_5H_{10}N)-, NH(*CH*-C_5H_{10})), 2.96 – 2.82 (m, 2H, -CO(NC_5H_{10}N)-), 2.80 – 2.67 (m, 2H, -CO(NC_5H_{10}N)-), 2.31 (s, 3H, Ar-CH_3), 2.03– 1.44 (m, 4H, -CO(NC_5H_{10}N)-, NH(CH-C_5H_{10})), 1.71 – 1.44 (m, 3H, NH(CH-C_5H_{10})), 1.40 – 1.19 (m, 2H, NH(CH-C_5H_{10})), 1.19 – 0.95 (m, 3H, NH(CH-C_5H_{10})). ^{13}C NMR (75 MHz, $CDCl_3$) δ 169.1, 164.1, 150.0, 149.8, 142.6, 141.3, 137.9, 135.8, 134.6, 134.5, 131.4, 130.3, 129.9, 129.8, 129.6, 129.2, 128.0, 126.1, 125.0, 124.8, 116.3, 69.3, 50.7, 50.5, 49.4, 47.2, 44.5, 32.7, 32.6, 26.2, 25.3, 24.8, 24.7, 9.1. LRMS (CI) calcd. for $C_{35}H_{38}Cl_3N_6O_2$ $[M+H]^+$: 679.21 found: 679.21.

***N*-benzyl-2-(4-(5-(4-chlorophenyl)-1-(2,4-dichlorophenyl)-4-methyl-1H-pyrazole-3-carbonyl)-1,4-diazepan-1-yl)-2-(pyridin-4-yl)acetamide (23n):** Yield: 38%; Mp: 196 – 197ºC. ^{1}H NMR (300 MHz, $CDCl_3$) δ 8.53 (brs, 1H, NH), 8.14 (d, *J* = 7.2 Hz, 1H, Ar-H), 7.79 (d, *J* = 6.9 Hz, 1H, Ar-H), 7.46 (d, *J* = 2.2 Hz, 1H, Ar-H), 7.42 – 7.20 (m, 10H, Ar-H), 7.19 – 7.12 (m, 1H, Ar-H), 7.11 – 7.05 (m, 2H, Ar-H), 4.49 (d, *J* = 6.0 Hz, 2H, NHCH_2Ph), 4.27 (s, 1H, -N*CH*(4-pyridyl)CO-), 3.96 – 3.65 (m, 4H, -CO(NC_5H_{10}N)-), 2.96 – 2.82 (m, 2H, -CO(NC_5H_{10}N)-), 2.80 – 2.67 (m, 2H, -CO(NC_5H_{10}N)-), 2.31 (s, 3H, Ar-CH_3), 2.03 – 1.88 (m, 2H, -CO(NC_5H_{10}N)-). ^{13}C NMR (75 MHz, $CDCl_3$) δ 169.2, 164.2, 149.9, 149.8, 142.8, 141.5, 138.7, 137.7, 136.0, 134.4, 134.2, 131.5, 130.0, 129.8, 129.8, 129.6, 129.1, 128.4, 128.3, 127.6, 127.5, 127.4, 127.4, 126.3, 125.0, 124.7, 116.0, 69.13, 50.6, 50.4, 47.2, 44.6, 43.9, 26.1, 9.12. LRMS (CI) calcd. for $C_{36}H_{34}Cl_3N_6O_2$ $[M+H]^+$: 687.18 found: 687.19.

1-(2-(tert-butylamino)-2-oxoethyl)-4-(5-(4-chlorophenyl)-1-(2,4-dichlorophenyl)-4-methyl-1H-pyrazole-3-carbonyl)-1-methylpiperazin-1-ium iodide (24a): Yield: 65%; Mp: 207 – 209ºC. ^{1}H NMR (300 MHz, $CDCl_3$) δ 8.05 (brs, 1H, NH), 7.45 (d, *J* = 2.2 Hz, 1H, Ar-H), 7.36 – 7.23 (m, 3H, Ar-H), 7.18 (d, *J* = 8.4 Hz, 1H, Ar-H), 7.06 (d, *J* = 8.1 Hz, 2H, Ar-H), 4.99 – 4.65 (m, 2H, -CO(NC_4H_8N)-), 4.56 – 4.42 (m, 2H, -CO(NC_4H_8N)-), 4.28 – 4.05 (m, 4H, -CO(NC_4H_8N)-), 3.78 – 3.62 (m, 2H, -N(CH_3)CH_2CO-), 3.60 (s, 3H, NCH_3), 2.23 (s, 3H, Ar-CH_3), 1.40 (s, 9H, NHC(CH_3)$_3$). ^{13}C NMR (75 MHz, $CDCl_3$) δ 162.9, 161.6, 144.2, 142.8, 136.2, 135.5, 135.2, 132.9, 130.7, 130.4, 129.0, 128.0, 126.7, 118.8, 61.6, 53.2, 49.92 49.8, 28.4, 9.3. HRMS (ESI) calcd. for $C_{28}H_{33}Cl_3N_5O_2^+$ $[M]^+$: 576.1694 found: 576.1689.

4-(5-(4-chlorophenyl)-1-(2,4-dichlorophenyl)-4-methyl-1H-pyrazole-3-carbonyl)-1-(2-(cyclohexylamino)-2-oxoethyl)-1-methylpiperazin-1-ium iodide (24b): Yield: 77%; Mp:162 – 164ºC. ^{1}H NMR (300 MHz, $CDCl_3$) [rotamers mixture] δ 8.38 (br d, *J* = 7.9 Hz, 1H, NH), 7.49 (s, 1H, Ar-H), 7.37 – 7.26 (m, 3H, Ar-H), 7.19 (d, *J* = 8.4 Hz, 1H, Ar-H), 7.09 (d, *J* = 8.0 Hz, 2H, Ar-H), 5.03 – 4.88 (m, 1H, -CO(NC_4H_8N)-), 4.84 – 4.70 (m, 1H, -CO(NC_4H_8N)-), 4.55 – 4.44 (m, 2H, -CO(NC_4H_8N)-), 4.34 – 4.03 (m, 4H, -CO(NC_4H_8N)-), 3.93 – 3.63 (m, 3H, -N(CH_3)CH_2CO-, NH(*CH*-C_5H_{10})), 3.60 (s, 3H, NCH_3), 2.26 (s, 3H, Ar-CH_3), 1.96 – 1.85 (m, 2H, NH(CH-C_5H_{10})), 1.84 – 1.71 (m, 2H, NH(CH-C_5H_{10})), 1.55 – 1.15 (m, 6H, NH(CH-C_5H_{10})). ^{13}C NMR (75 MHz, $CDCl_3$) δ 162.8, 161.2, 144.2, 142.8, 136.2, 135.5, 135.2, 132.8, 130.7, 130.5, 130.4, 129.0, 128.0, 126.8, 118.7, 61.7, 61.4, 50.2, 49.5, 32.2, 25.2, 24.7, 9.3. LRMS (CI) calcd. for $C_{30}H_{35}Cl_3N_5O_2^+$ $[M]^+$: 602.18 found: 602.18.

1-(2-(benzylamino)-2-oxoethyl)-4-(5-(4-chlorophenyl)-1-(2,4-dichlorophenyl)-4-methyl-1H -pyrazole-3-carbonyl)-1-methylpiperazin-1-ium iodide (24c): Yield: 76%; Mp: 191 – 193ºC. ^{1}H NMR (300 MHz, $CDCl_3$) δ 8.85 (br t, *J* = 6.2 Hz, 1H, NH), 7.44 (d, *J* = 1.3 Hz, 1H, Ar-H), 7.39 – 7.12 (m, 9H, Ar-H), 7.05 (d, *J* = 8.5 Hz, 2H, Ar-H), 5.06 – 4.88 (m, 1H, -CO(NC_4H_8N)-), 4.87 – 4.73 (m, 1H, -CO(NC_4H_8N)-), 4.54 – 4.35 (m, 4H, -CO(NC_4H_8N)-, NHCH_2Ph), 4.28 – 4.12 (m, 2H, -CO(NC_4H_8N)-), 4.10 – 3.92 (m, 2H, -CO(NC_4H_8N)-), 3.71 – 3.59 (m, 2H, -N(CH_3)CH_2CO-), 3.52 (s, 3H, NCH_3), 2.21 (s, 3H, Ar-CH_3). ^{13}C NMR (75 MHz, $CDCl_3$) δ 162.9, 162.3, 144.2, 142.7, 137.1, 136.2, 135.5, 135.2, 132.8, 130.8, 130.5, 130.4, 129.0, 128.7, 128.0, 127.6, 126.7, 118.7, 61.6, 61.3, 50.2, 43.5, 41.2, 36.3, 9.4. LRMS (CI) calcd. for $C_{31}H_{31}Cl_3N_5O_2^+$ $[M]^+$: 610.15 found: 610.15.

1-(2-(tert-butylamino)-2-oxoethyl)-4-(5-(4-chlorophenyl)-1-(2,4-dichlorophenyl)-4-methyl-1H-pyrazole-3-carbonyl)-1-methyl-1,4-diazepan-1-ium iodide (25a): Yield: 55%; Mp: 159 – 160ºC. ^{1}H NMR (300 MHz, $CDCl_3$) [rotamers mixture] δ 8.22 (br s, 0.5H, NH), 8.15 (br s, 0.5H, NH), 7.50 (d, *J* = 2.3 Hz, 1H, Ar-H), 7.38 – 7.24 (m, 3H, Ar-H), 7.20 – 7.11 (m, 1H, Ar-H), 7.07 (d, *J* = 8.4 Hz, 2H, Ar-H), 5.08 – 4.84 (m, 1H, -CO(NC_5H_{10}N)-), 4.56 – 3.83 (m, 6H, -CO(NC_5H_{10}N)-), 3.78 – 3.60 (m, 3H, -CO(NC_5H_{10}N)-, -N(CH_3)CH_2CO-), 3.55 – 3.40 (m, 3H, NCH_3), 2.73 – 2.27 (m, 2H, -CO(NC_5H_{10}N)-), 2.24 (s, 3H, Ar-CH_3), 1.44 (s, 6H, NHC(CH_3)$_3$), 1.27 (s, 3H, NHC(CH_3)$_3$). ^{13}C NMR (75 MHz, $CDCl_3$) [rotamers mixture] δ 164.7, 161.8, 145.4, 142.6, 142.4, 136.1, 135.6, 135.2, 132.8, 132.7, 130.7, 130.5, 130.3, 129.0, 128.2, 128.0, 126.8, 117.7, 70.3, 69.0, 65.0, 63.9, 63.7, 61.8, 53.1, 43.4, 41.0, 29.7, 28.4, 23.8, 21.8, 9.2. LRMS (CI) calcd. for $C_{29}H_{35}Cl_3N_5O_2^+$ $[M]^+$: 590.18 found: 590.19.

4-(5-(4-chlorophenyl)-1-(2,4-dichlorophenyl)-4-methyl-1H-pyrazole-3-carbonyl)-1-(2-(cyclohexylamino)-2-oxoethyl)-1-methyl-1,4-diazepan-1-ium iodide (25b): Yield: 67%; Mp: 154 – 156ºC. ^{1}H NMR (300 MHz, $CDCl_3$) [rotamers mixture] δ 8.37 (br d, *J* = 7.8 Hz, 0.5H, NH), 8.26 (br d, *J* = 7.7 Hz, 0.5H, NH), 7.46 (d, *J* = 2.3 Hz, 1H, Ar-H), 7.34 – 7.21 (m, 3H, Ar-H), 7.21 – 7.07 (m, 1H, Ar-H), 7.05 (d, *J* = 8.4 Hz, 2H, Ar-H), 5.02 – 4.88 (m, 1H, -CO(NC_5H_{10}N)-), 4.63 – 4.46 (m, 1H, -CO(NC_5H_{10}N)-), 4.42 – 3.63 (m, 9H, -CO(NC_5H_{10}N)-, NH(*CH*-C_5H_{10}), -N(CH_3)CH_2CO-), 3.55 (s, 3H, NCH_3), 2.76 – 2.25 (m, 2H, -CO(NC_5H_{10}N)-), 2.21 (s, 3H, Ar-CH_3), 1.93 – 1.66 (m, 4H, NH(CH-C_5H_{10})), 1.63 – 1.14 (m, 6H, NH(CH-C_5H_{10})). ^{13}C NMR (75 MHz, $CDCl_3$) [rotamers mixture] δ 164.7, 161.5, 161.4, 145.4, 142.6, 142.4, 136.0, 135.6, 135.5, 135.2, 132.8, 132.7, 130.7, 130.5, 130.3, 129.0, 128.2, 128.0, 126.8, 126.8, 118.3, 117.7, 69.1, 65.1, 63.9, 61.4, 52.6, 49.5, 46.4, 43.5, 43.3, 41.0, 32.3, 32.1, 29.7, 25.2, 24.7, 21.7, 23.8, 9.24. LRMS (CI) calcd. for $C_{31}H_{37}Cl_3N_5O_2^+$ $[M]^+$: 616.20 found: 616.20.

1-(2-(benzylamino)-2-oxoethyl)-4-(5-(4-chlorophenyl)-1-(2,4-dichlorophenyl)-4-methyl-1H -pyrazole-3-carbonyl)-1-methyl-1,4-diazepan-1-ium iodide (25c): Yield: 68%; Mp: 159 – 160ºC. ^{1}H NMR (300 MHz, $CDCl_3$) [rotamers mixture] δ 8.92 (br t, *J* = 6.1 Hz, 0.5H, NH), 8.81 (br t, *J* = 6.1 Hz, 0.5H, NH), 7.45 (s, 1H, Ar-H), 7.39 – 7.21 (m, 8H, Ar-H), 7.20 – 7.06 (m, 1H, Ar-H), 7.04 (d, *J* = 8.1 Hz, 2H, Ar-H), 5.09 – 4.85 (m, 1H, -CO(NC_5H_{10}N)-), 4.73 – 4.50 (m, 1H, -CO(NC_5H_{10}N)-), 4.51 – 3.54 (m, 10H, -CO(NC_5H_{10}N)-, -N(CH_3)CH_2CO-, NHCH_2Ph), 3.47 (s, 3H, NCH_3), 2.61 – 2.24 (m, 2H, -CO(NC_5H_{10}N)-), 2.19 (s, 3H, Ar-CH_3). ^{13}C NMR (75 MHz, $CDCl_3$) [rotamers mixture] δ 164.7, 164.6, 162.6, 162.5, 145.4, 142.4, 137.2, 136.0, 135.7, 135.5, 135.1, 132.8, 132.6, 130.7, 130.5, 129.0, 128.7, 128.2, 128.0, 127.6, 126.9, 118.2, 117.7, 69.8, 68.9, 64.9, 63.9, 61.6, 61.4, 52.4, 46.4, 43.5, 40.9, 29.7, 23.8, 21.7, 9.2. LRMS (CI) calcd. for $C_{32}H_{33}Cl_3N_5O_2^+$ $[M]^+$: 624.17 found: 624.18.

HPLC purity analysis of representative compounds

SAMPLE INFORMATION

Sample Name:	20b	Acquired By:	System
Sample Type:	Unknown	Sample Set Name:	Rimonabant
Vial:	3	Acq. Method Set:	Gradiente_75_25_15min
Injection #:	1	Processing Method:	js40_254
Injection Volume:	10.00 ul	Channel Name:	254.0nm
Run Time:	15.0 Minutes	Proc. Chnl. Descr.:	2998 PDA 254.0 nm (2998

Date Acquired: 11/28/2023 2:09:38 PM CET
Date Processed: 11/28/2023 5:22:12 PM CET

Auto-Scaled Chromatogram

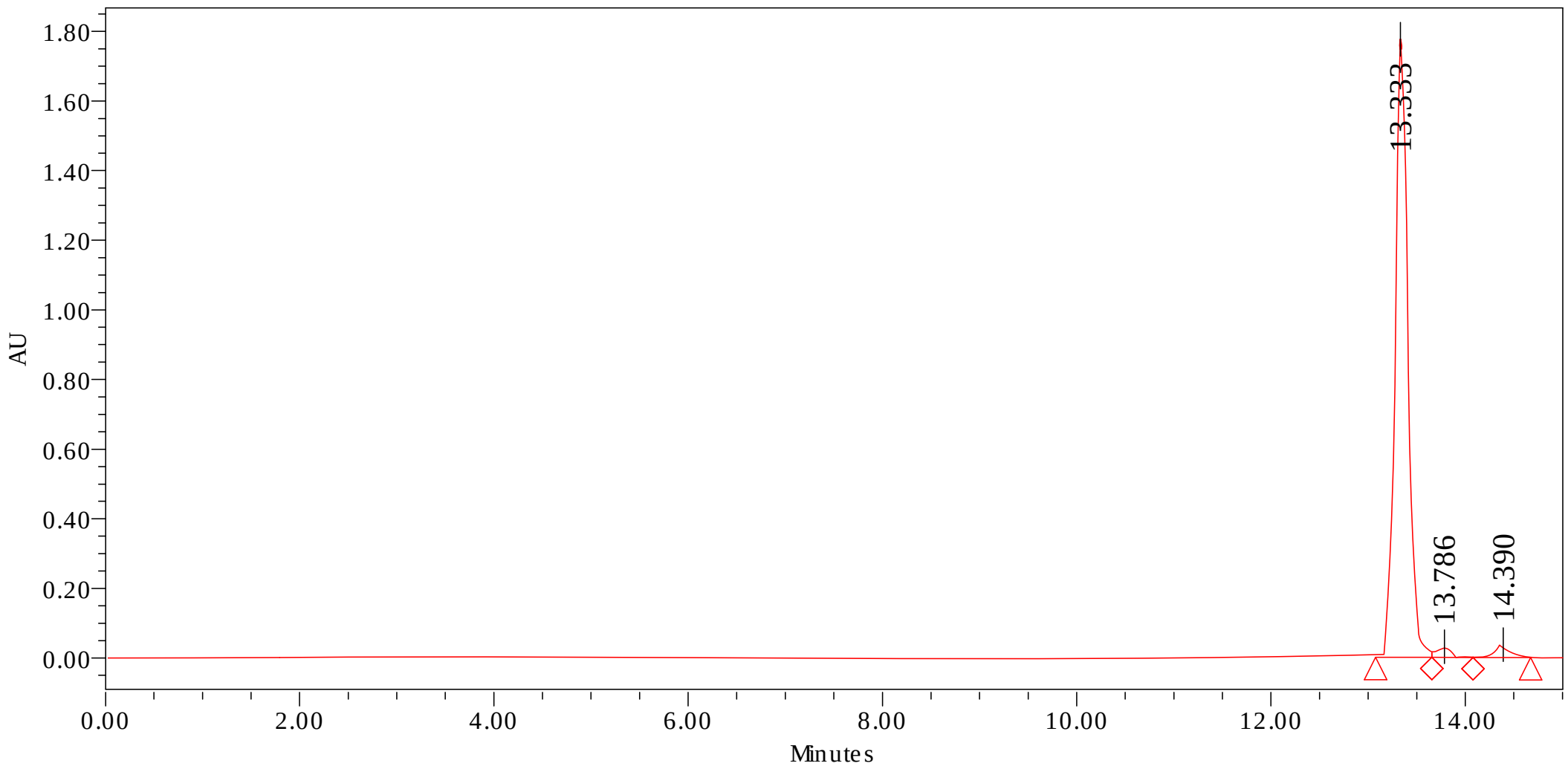

Peak Results

	RT	Area	% Area	Height
1	13.333	17275449	96.44	1775800
2	13.786	279201	1.56	30295
3	14.390	359197	2.01	36689

SAMPLE INFORMATION

Sample Name:	21b	Acquired By:	System
Sample Type:	Unknown	Sample Set Name:	Ruben
Vial:	4	Acq. Method Set:	Gradiente_75_25_15min
Injection #:	1	Processing Method:	js45_254
Injection Volume:	30.00 ul	Channel Name:	254.0nm
Run Time:	15.0 Minutes	Proc. Chnl. Descr.:	2998 PDA 254.0 nm (2998
Date Acquired:	11/28/2023 3:51:30 PMCET		
Date Processed:	11/28/2023 5:25:07 PMCET		

Auto-Scaled Chromatogram

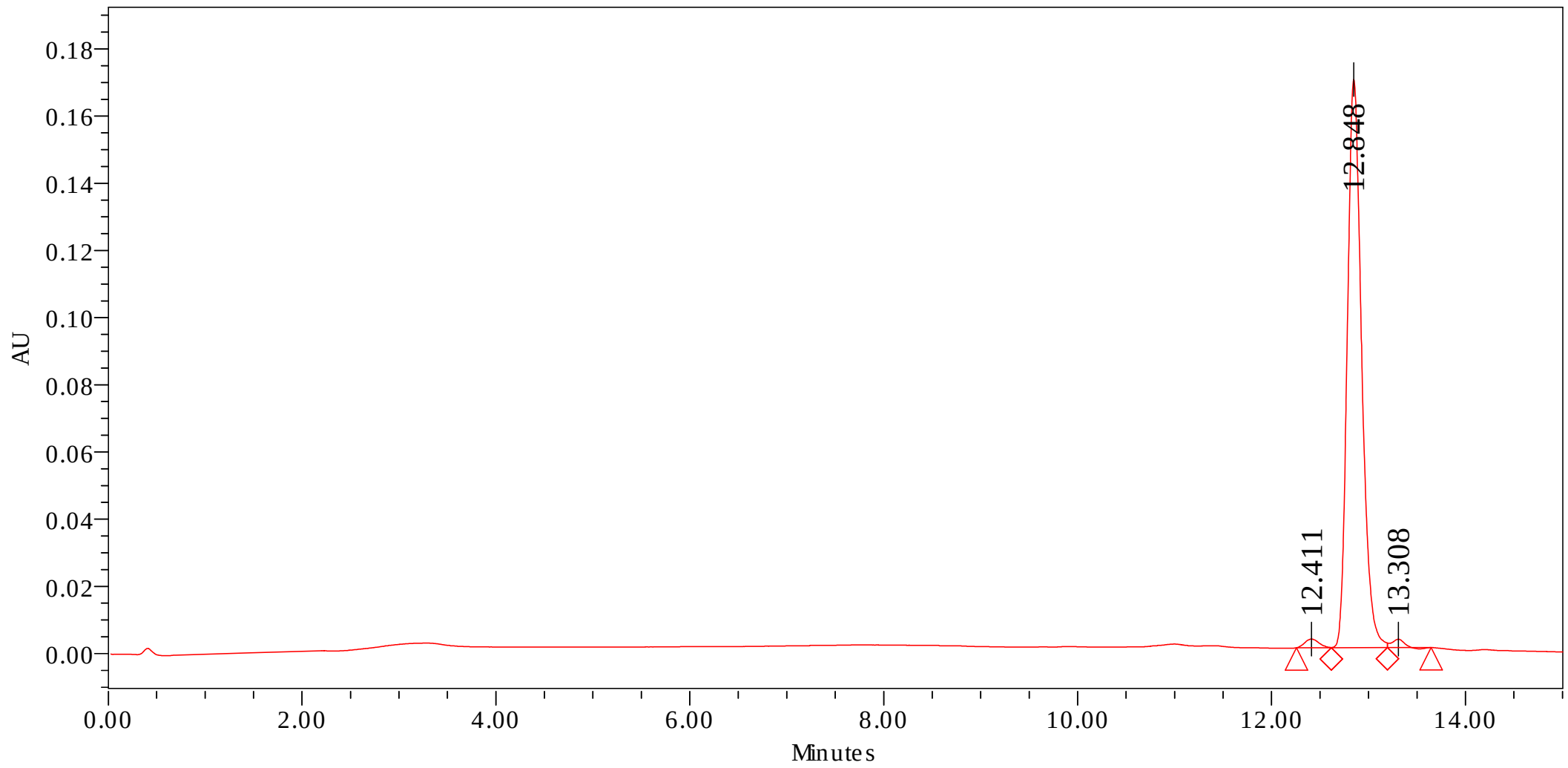

Peak Results

	RT	Area	% Area	Height
1	12.411	26024	1.46	2603
2	12.848	1736990	97.16	169173
3	13.308	24717	1.38	2542

660

SAMPLE INFORMATION

Sample Name:	21l	Acquired By:	System
Sample Type:	Unknown	Sample Set Name:	Rimonabant
Vial:	10	Acq. Method Set:	Gradiente_75_25_15min
Injection #:	1	Processing Method:	npf40_254
Injection Volume:	10.00 ul	Channel Name:	254.0nm
Run Time:	15.0 Minutes	Proc. Chnl. Descr.:	2998 PDA 254.0 nm (2998
Date Acquired:	11/28/2023 1:37:52 PM CET		
Date Processed:	11/28/2023 5:20:46 PM CET		

Auto-Scaled Chromatogram

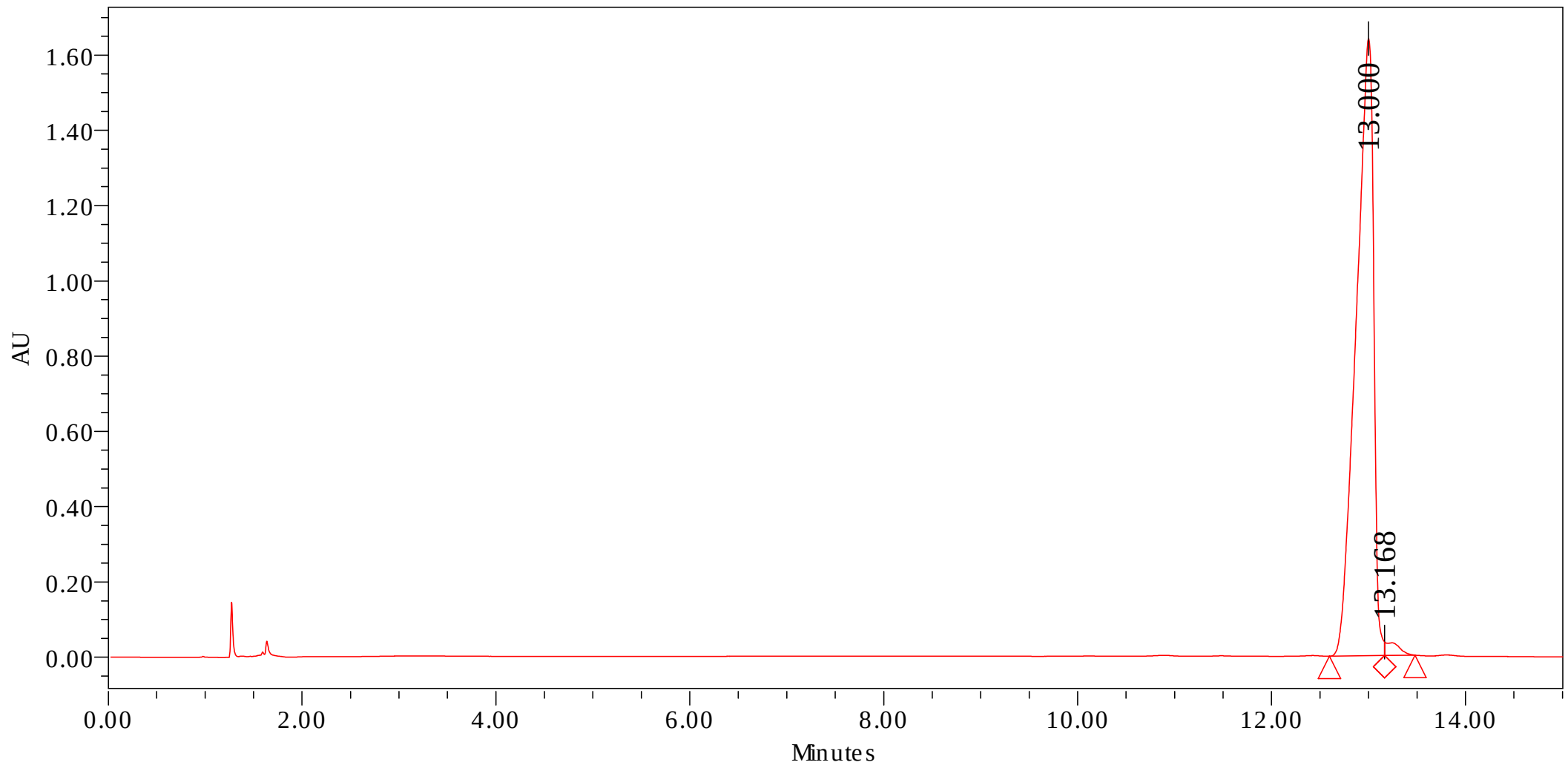

Peak Results

	RT	Area	% Area	Height
1	13.000	20482079	98.35	1640219
2	13.168	344287	1.65	35691

661

S A M P L E   I N F O R M A T I O N

Sample Name:	22a	Acquired By:	System
Sample Type:	Unknown	Sample Set Name:	Rimonabant
Vial:	6	Acq. Method Set:	Gradiente_75_25_15min
Injection #:	1	Processing Method:	mm47_254
Injection Volume:	10.00 ul	Channel Name:	254.0nm
Run Time:	15.0 Minutes	Proc. Chnl. Descr.:	2998 PDA 254.0 nm (2998
Date Acquired:	11/28/2023 12:06:12 PMCET		
Date Processed:	11/28/2023 5:18:06 PMCET		

Auto-Scaled Chromatogram

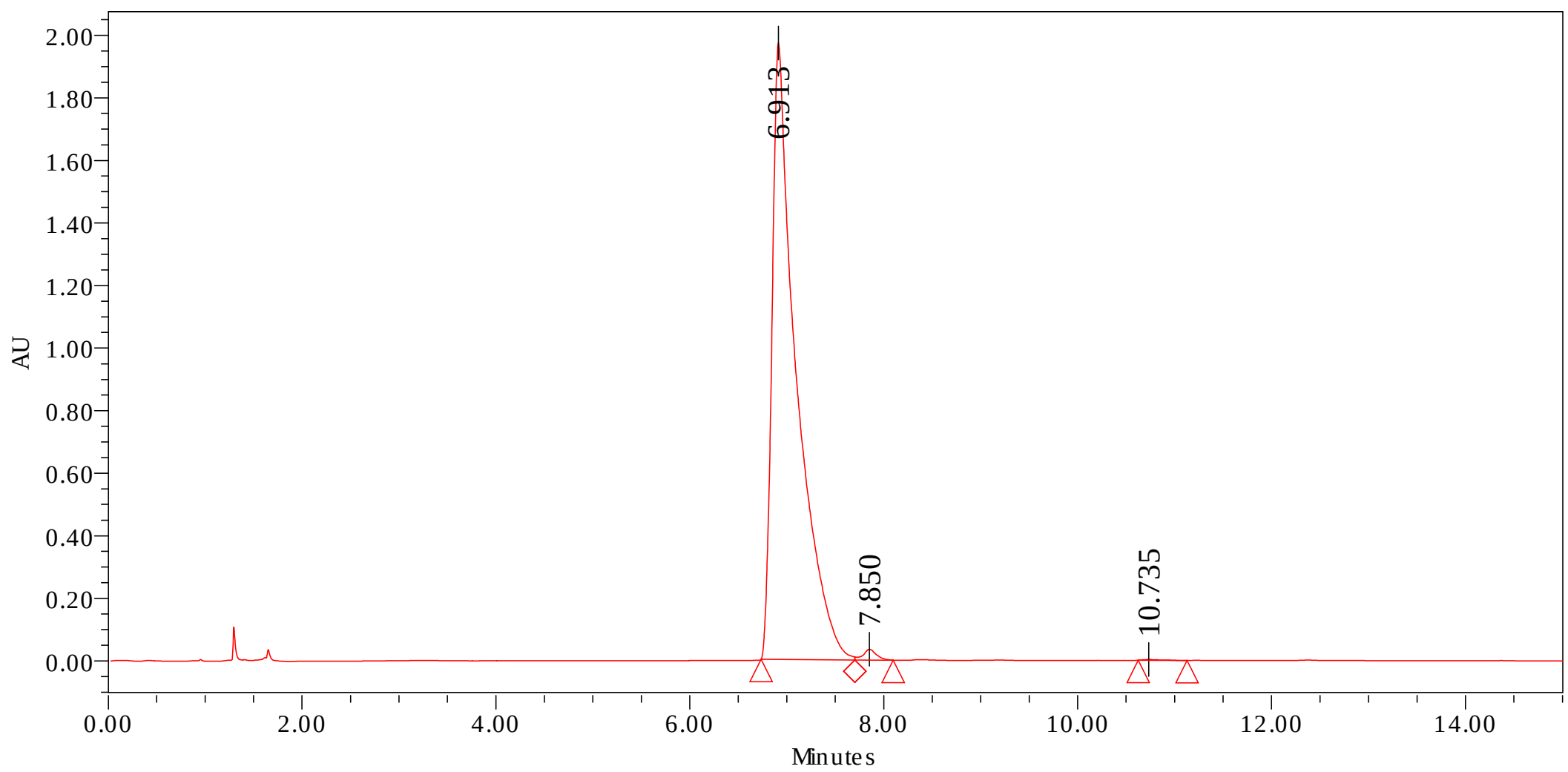

Peak Results

	RT	Area	% Area	Height
1	6.913	34206679	98.99	1971186
2	7.850	319600	0.92	34069
3	10.735	28640	0.08	2017

662

SAMPLE INFORMATION

Sample Name:	22b	Acquired By:	System
Sample Type:	Unknown	Sample Set Name:	Rimonabant
Vial:	8	Acq. Method Set:	Gradiente_75_25_15min
Injection #:	1	Processing Method:	mm95_254
Injection Volume:	10.00 ul	Channel Name:	254.0nm
Run Time:	15.0 Minutes	Proc. Chnl. Descr.:	2998 PDA 254.0 nm (2998
Date Acquired:	11/28/2023 1:06:03 PMCET		
Date Processed:	11/28/2023 5:20:04 PMCET		

Auto-Scaled Chromatogram

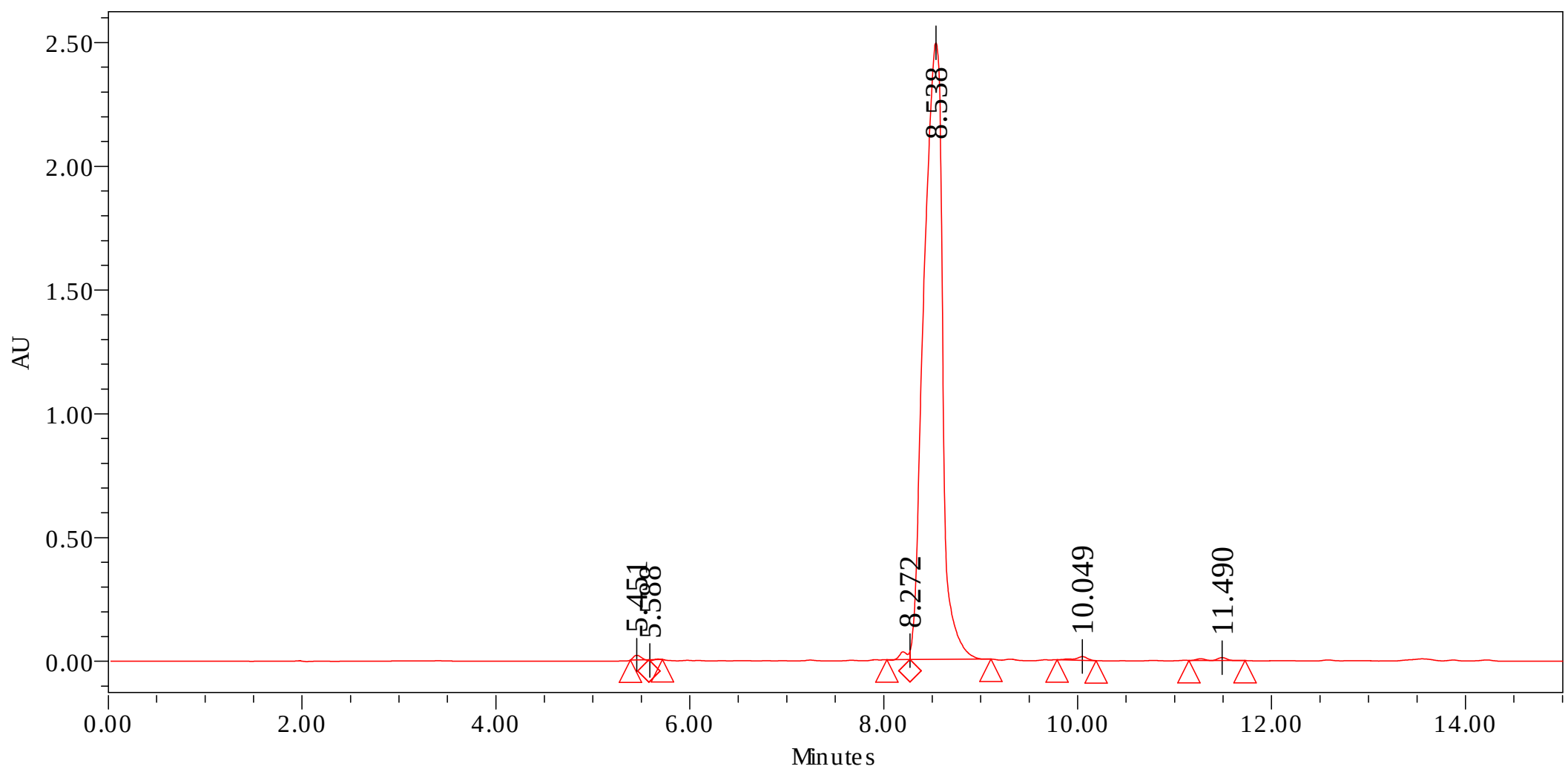

Peak Results

	RT	Area	% Area	Height
1	5.451	97822	0.29	18594
2	5.588	13517	0.04	-3232
3	8.272	196199	0.58	35282
4	8.538	33310960	98.41	2490933
5	10.049	123342	0.36	14519
6	11.490	108344	0.32	11343

663

SAMPLE INFORMATION

Sample Name:	22c	Acquired By:	System
Sample Type:	Unknown	Sample Set Name:	Rimonabant
Vial:	11	Acq. Method Set:	Gradiente_75_25_15min
Injection #:	1	Processing Method:	mm96_254
Injection Volume:	10.00 ul	Channel Name:	254.0nm
Run Time:	15.0 Minutes	Proc. Chnl. Descr.:	2998 PDA 254.0 nm (2998
Date Acquired:	11/28/2023 1:53:44 PMCET		
Date Processed:	11/28/2023 5:21:40 PMCET		

Auto-Scaled Chromatogram

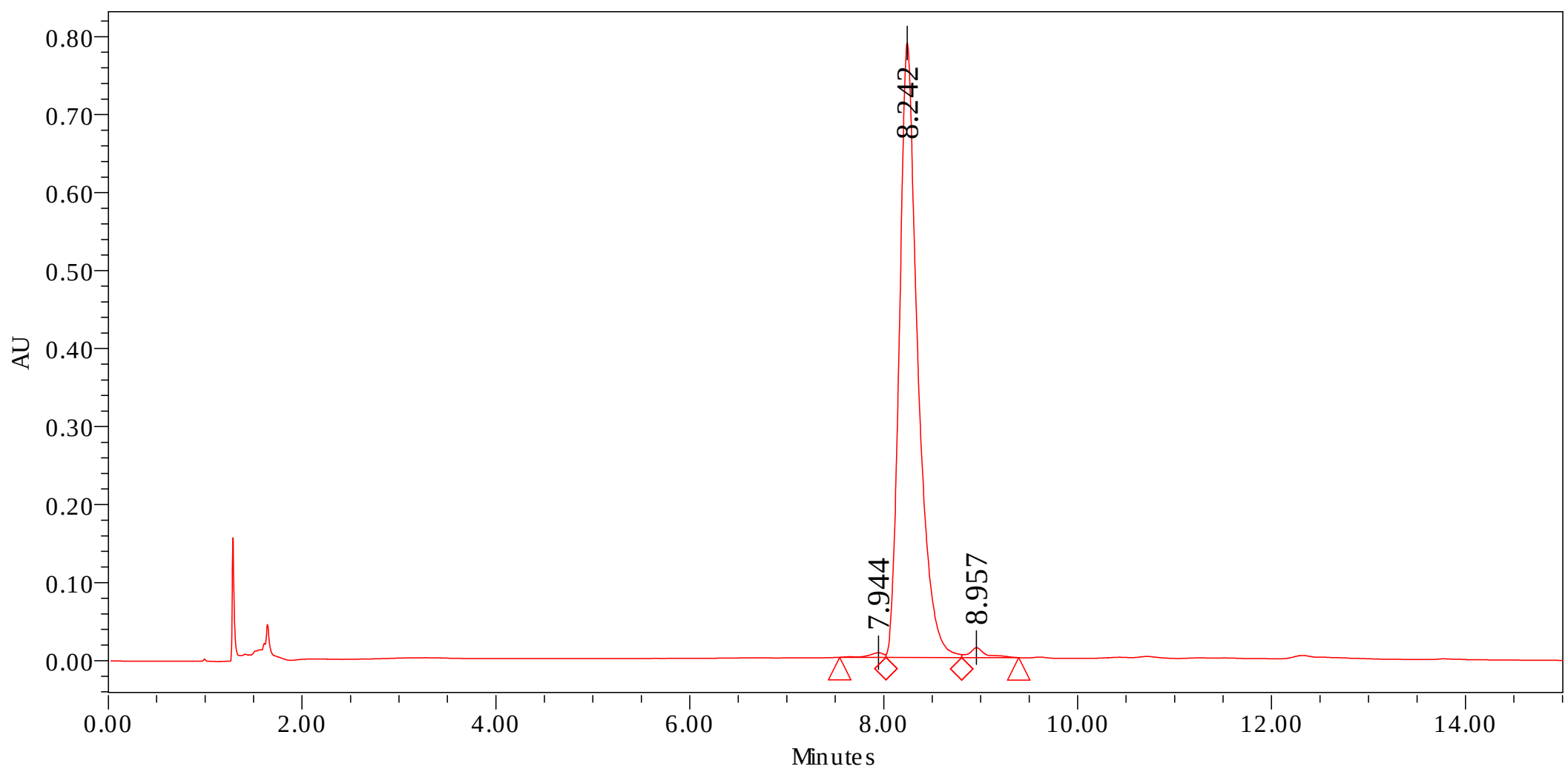

Peak Results

	RT	Area	% Area	Height
1	7.944	65151	0.62	6033
2	8.242	10373593	97.98	788073
3	8.957	148657	1.40	12753

664

SAMPLE INFORMATION

Sample Name:	22d	Acquired By:	System
Sample Type:	Unknown	Sample Set Name:	Rimonabant
Vial:	5	Acq. Method Set:	Gradiente_75_25_15min
Injection #:	1	Processing Method:	mm104_254
Injection Volume:	10.00 ul	Channel Name:	254.0nm
Run Time:	15.0 Minutes	Proc. Chnl. Descr.:	2998 PDA 254.0 nm (2998
Date Acquired:	11/28/2023 2:41:43 PM CET		
Date Processed:	11/28/2023 5:23:50 PM CET		

Auto-Scaled Chromatogram

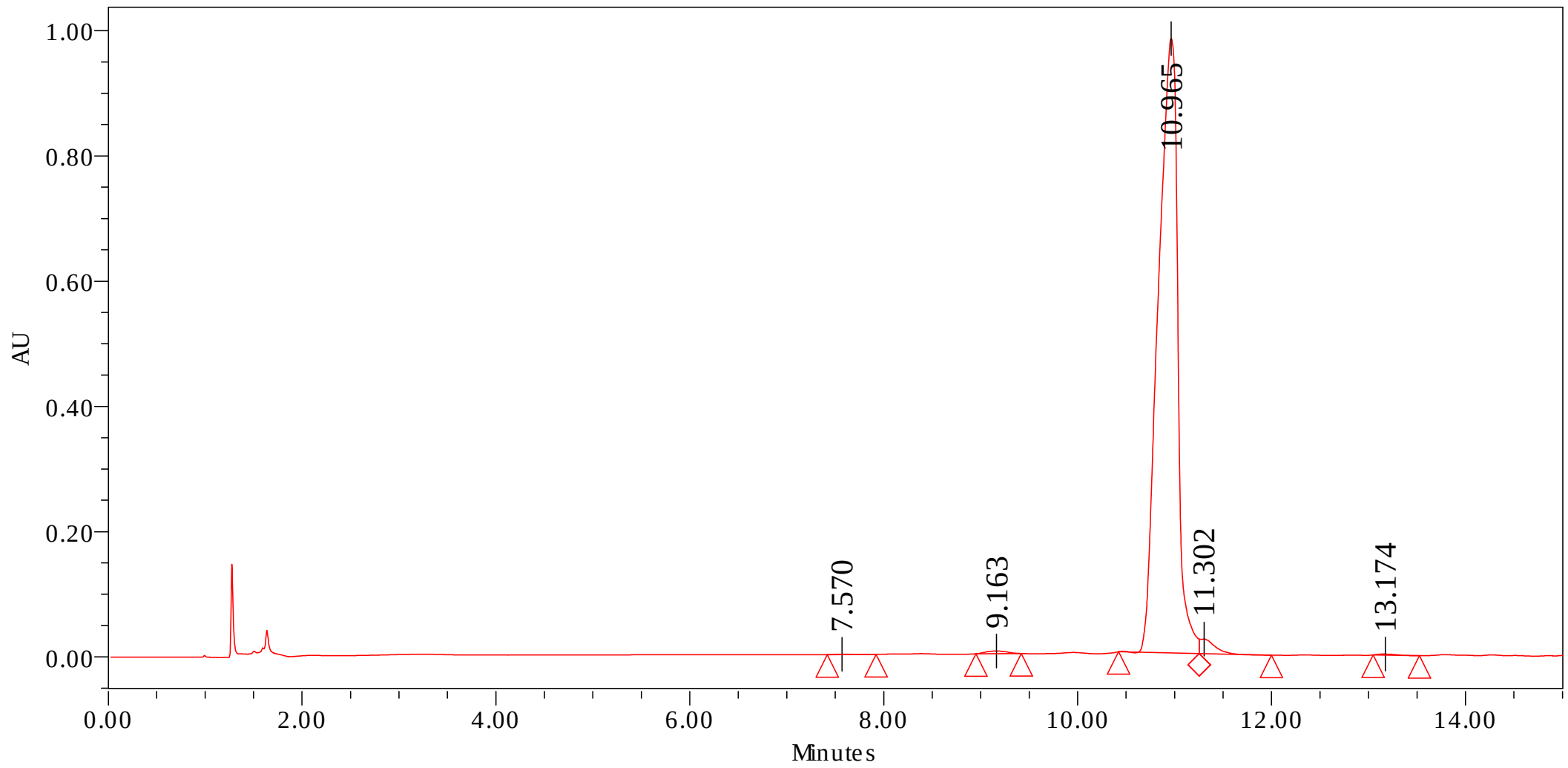

Peak Results

	RT	Area	% Area	Height
1	7.570	7380	0.05	635
2	9.163	66164	0.47	4626
3	10.965	13743361	97.55	981437
4	11.302	252630	1.79	23369
5	13.174	19133	0.14	1498

665

SAMPLE INFORMATION

Sample Name:	23a	Acquired By:	System
Sample Type:	Unknown	Sample Set Name:	Rimonabant
Vial:	1	Acq. Method Set:	Gradiente_75_25_10min
Injection #:	1	Processing Method:	dt18_254
Injection Volume:	10.00 ul	Channel Name:	254.0nm
Run Time:	10.0 Minutes	Proc. Chnl. Descr.:	2998 PDA 254.0 nm (2998
Date Acquired:	11/28/2023 10:02:53 AMCET		
Date Processed:	11/28/2023 5:14:50 PMCET		

Auto-Scaled Chromatogram

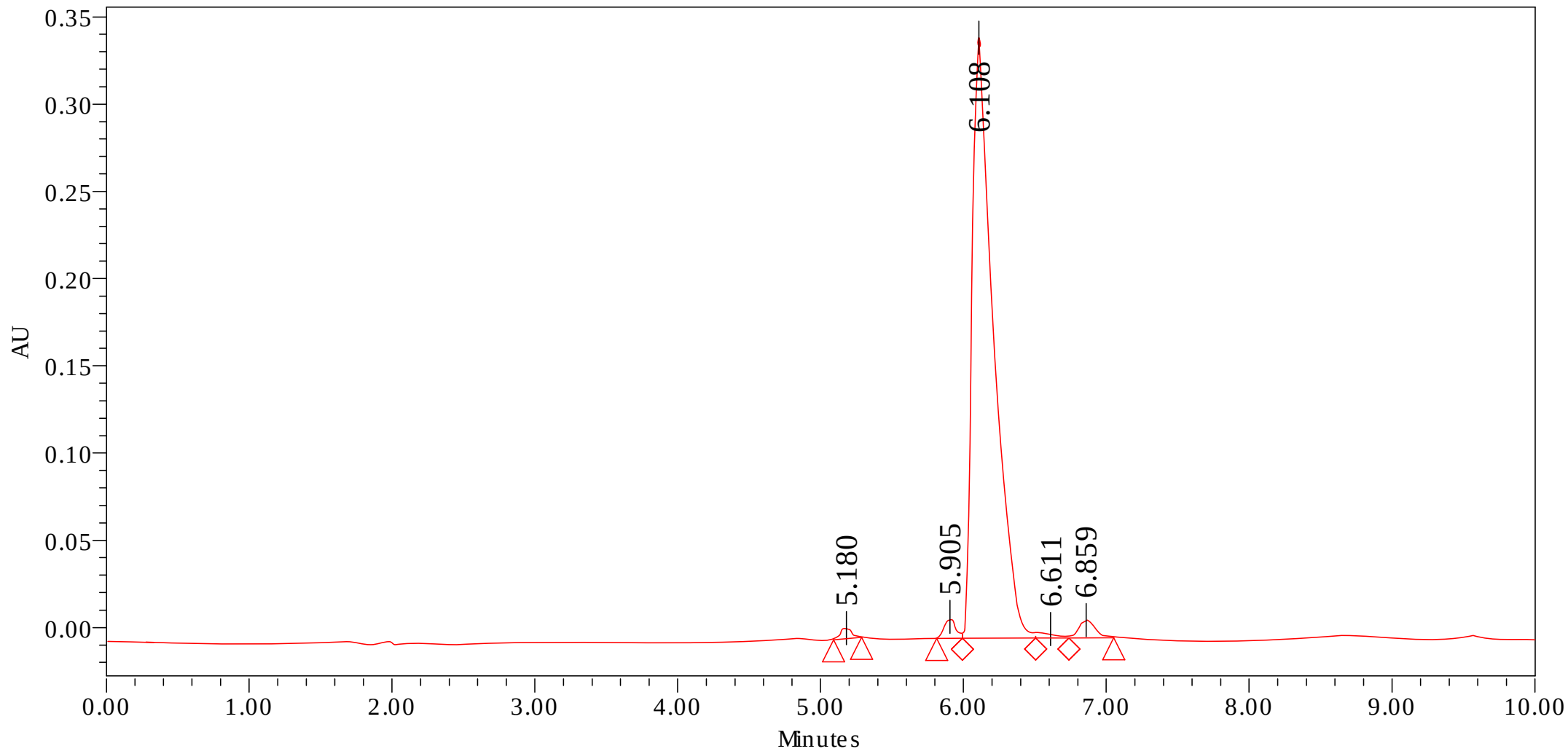

Peak Results

	RT	Area	% Area	Height
1	5.180	27737	0.72	6142
2	5.905	59818	1.55	12268
3	6.108	3679989	95.06	344158
4	6.611	31359	0.81	5025
5	6.859	72470	1.87	10142

666

SAMPLE INFORMATION

Sample Name:	23b	Acquired By:	System
Sample Type:	Unknown	Sample Set Name:	Rimonabant
Vial:	2	Acq. Method Set:	Gradiente_75_25_10min
Injection #:	1	Processing Method:	dt19_254
Injection Volume:	10.00 ul	Channel Name:	254.0nm
Run Time:	10.0 Minutes	Proc. Chnl. Descr.:	2998 PDA 254.0 nm (2998
Date Acquired:	11/28/2023 11:19:37 AM CET		
Date Processed:	11/28/2023 5:16:14 PM CET		

Auto-Scaled Chromatogram

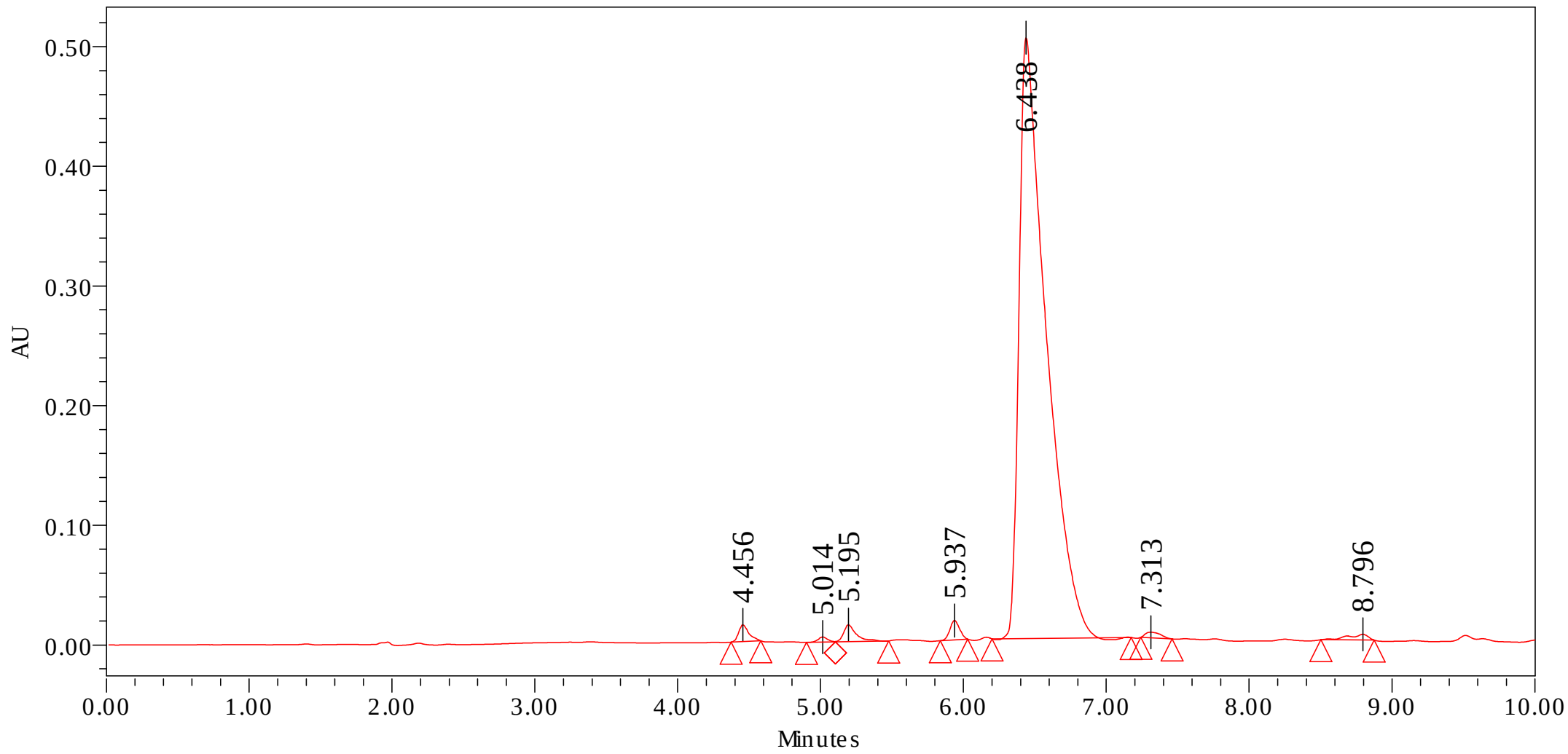

Peak Results

	RT	Area	% Area	Height
1	4.456	70438	1.01	13981
2	5.014	19302	0.28	4300
3	5.195	87856	1.26	14138
4	5.937	76713	1.10	16243
5	6.438	6649396	95.18	502080
6	7.313	34666	0.50	4630
7	8.796	47916	0.69	4787

667

SAMPLE INFORMATION

Sample Name:	23c	Acquired By:	System
Sample Type:	Unknown	Sample Set Name:	Ruben
Vial:	7	Acq. Method Set:	Gradiente_75_25_15min
Injection #:	1	Processing Method:	dt20_254
Injection Volume:	30.00 ul	Channel Name:	254.0nm
Run Time:	15.0 Minutes	Proc. Chnl. Descr.:	2998 PDA 254.0 nm (2998
Date Acquired:	11/28/2023 3:35:28 PM CET		
Date Processed:	11/28/2023 5:24:32 PM CET		

Auto-Scaled Chromatogram

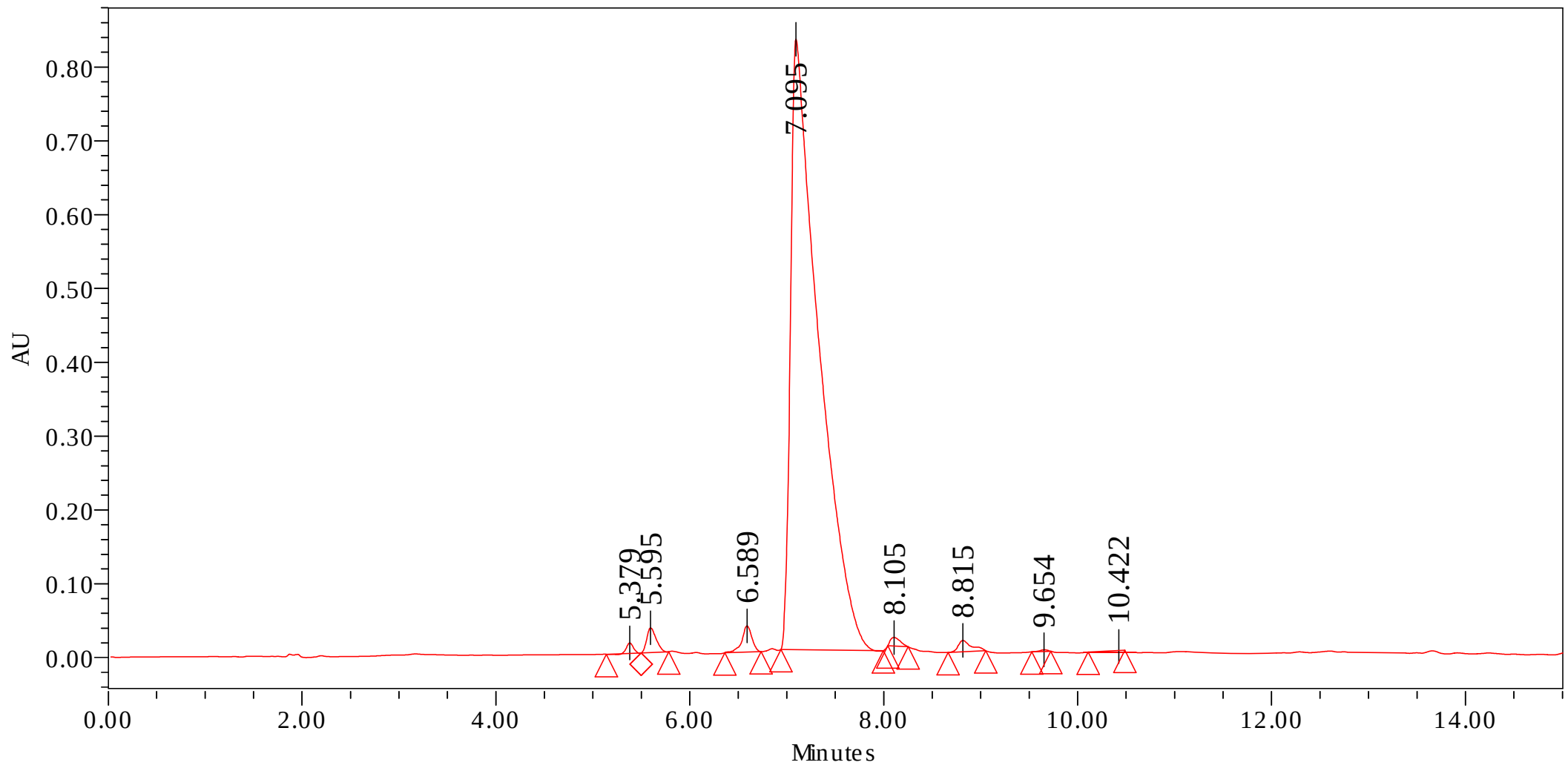

Peak Results

	RT	Area	% Area	Height
1	5.379	75545	0.43	14144
2	5.595	226370	1.30	33604
3	6.589	256312	1.47	35505
4	7.095	16593366	95.05	827008
5	8.105	81840	0.47	11477
6	8.815	146993	0.84	15358
7	9.654	13337	0.08	2353
8	10.422	63463	0.36	5377

NMR spectra of representative compounds

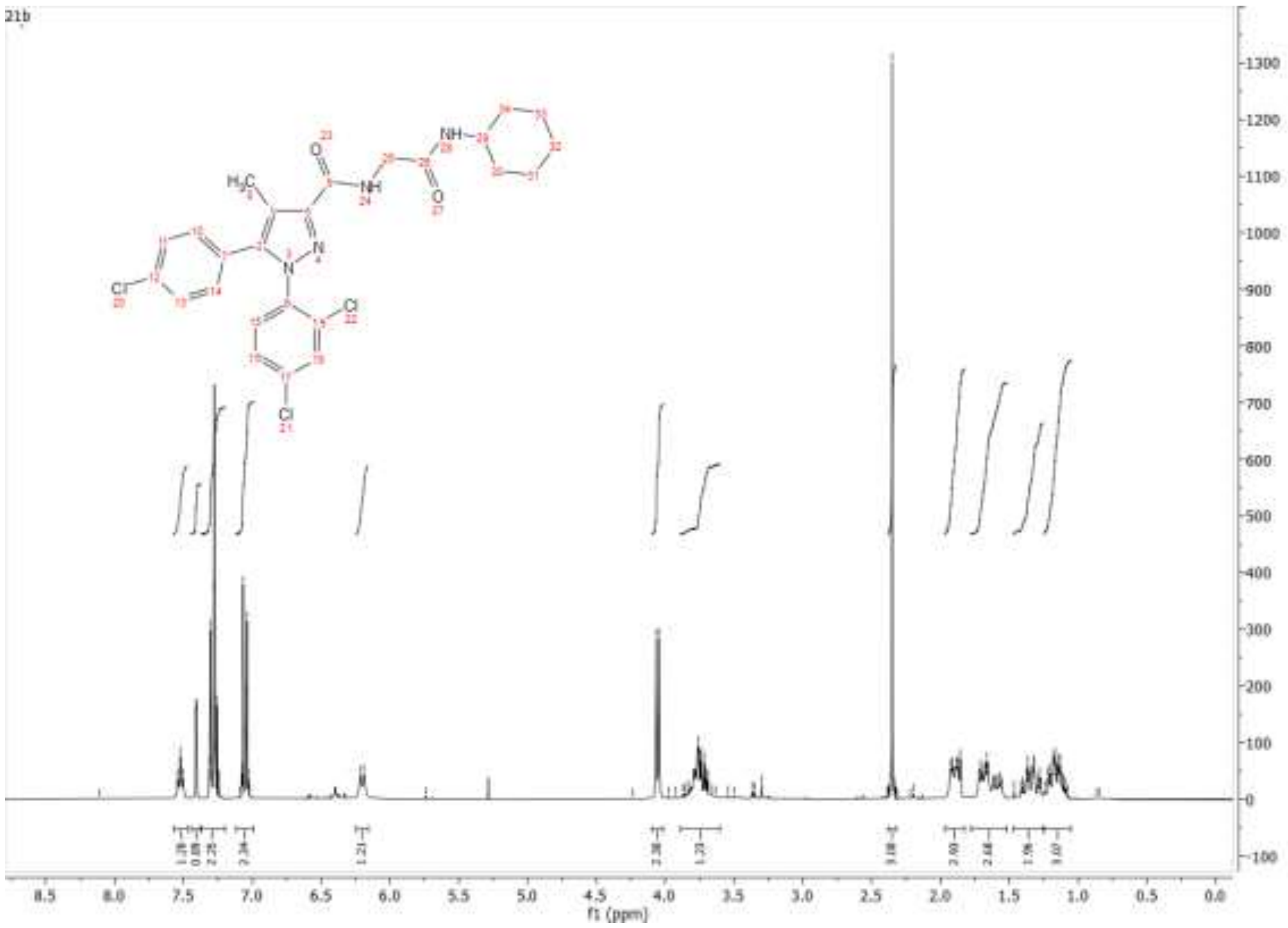

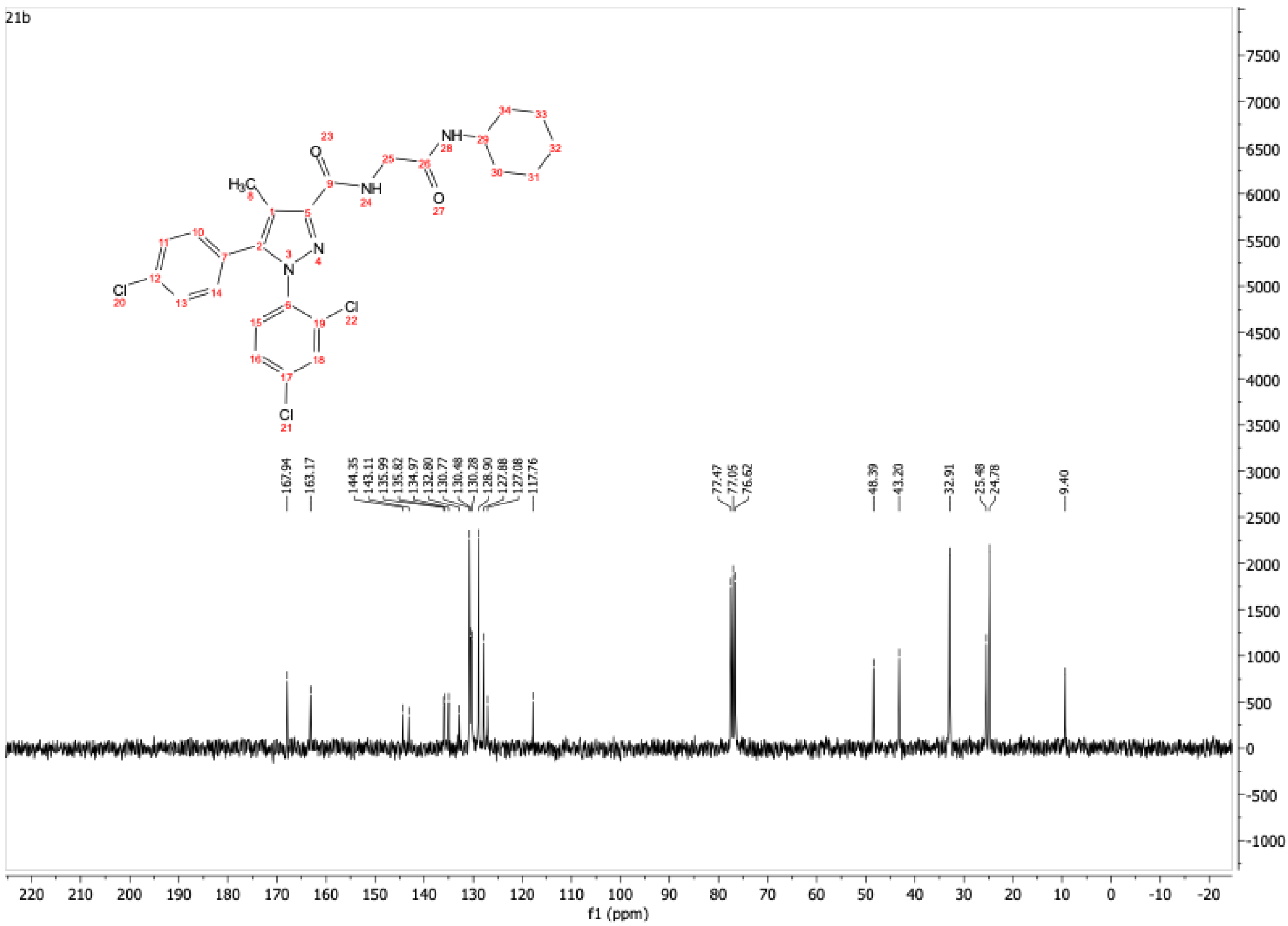

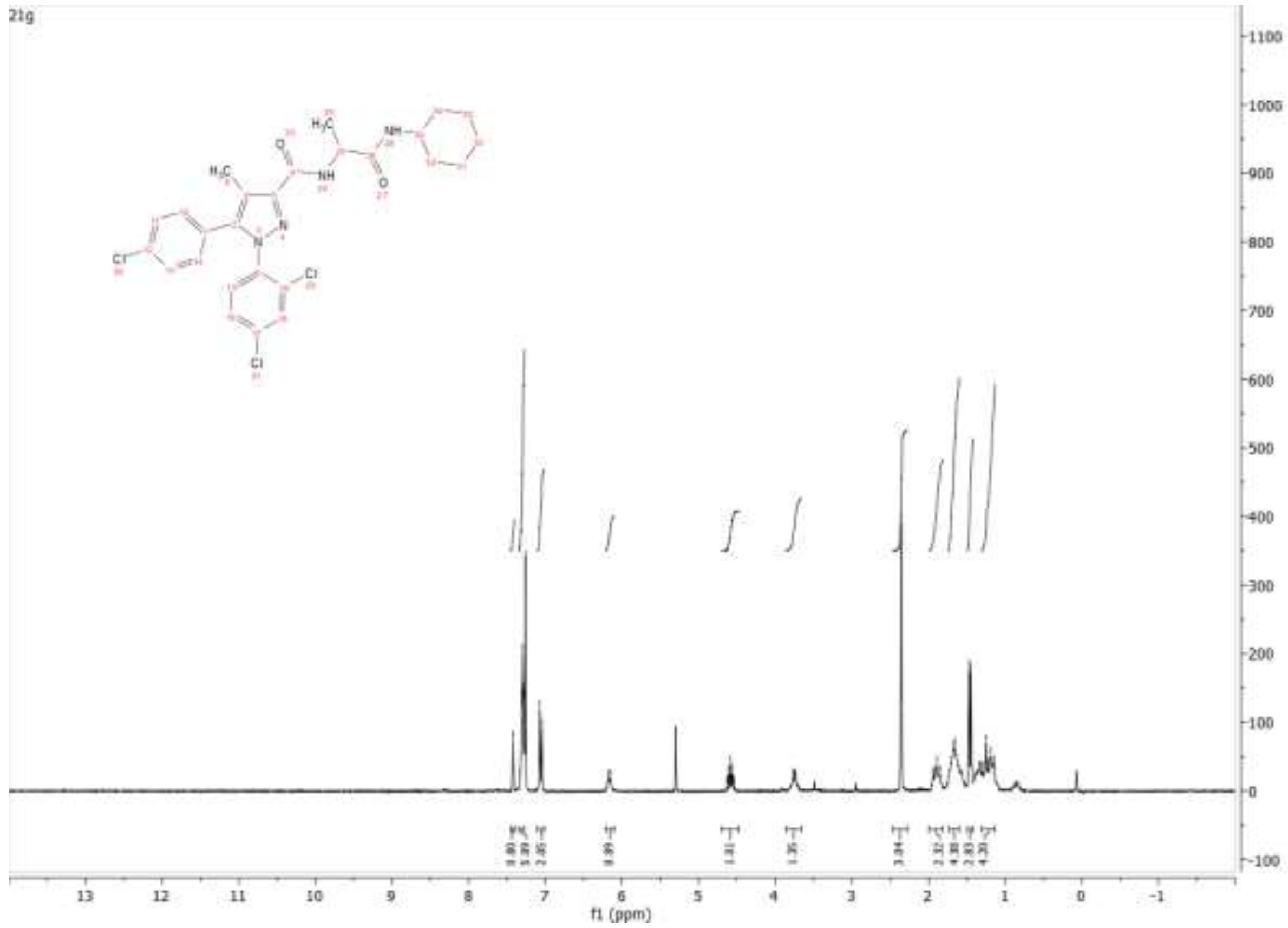
21g
f1 (ppm)

673

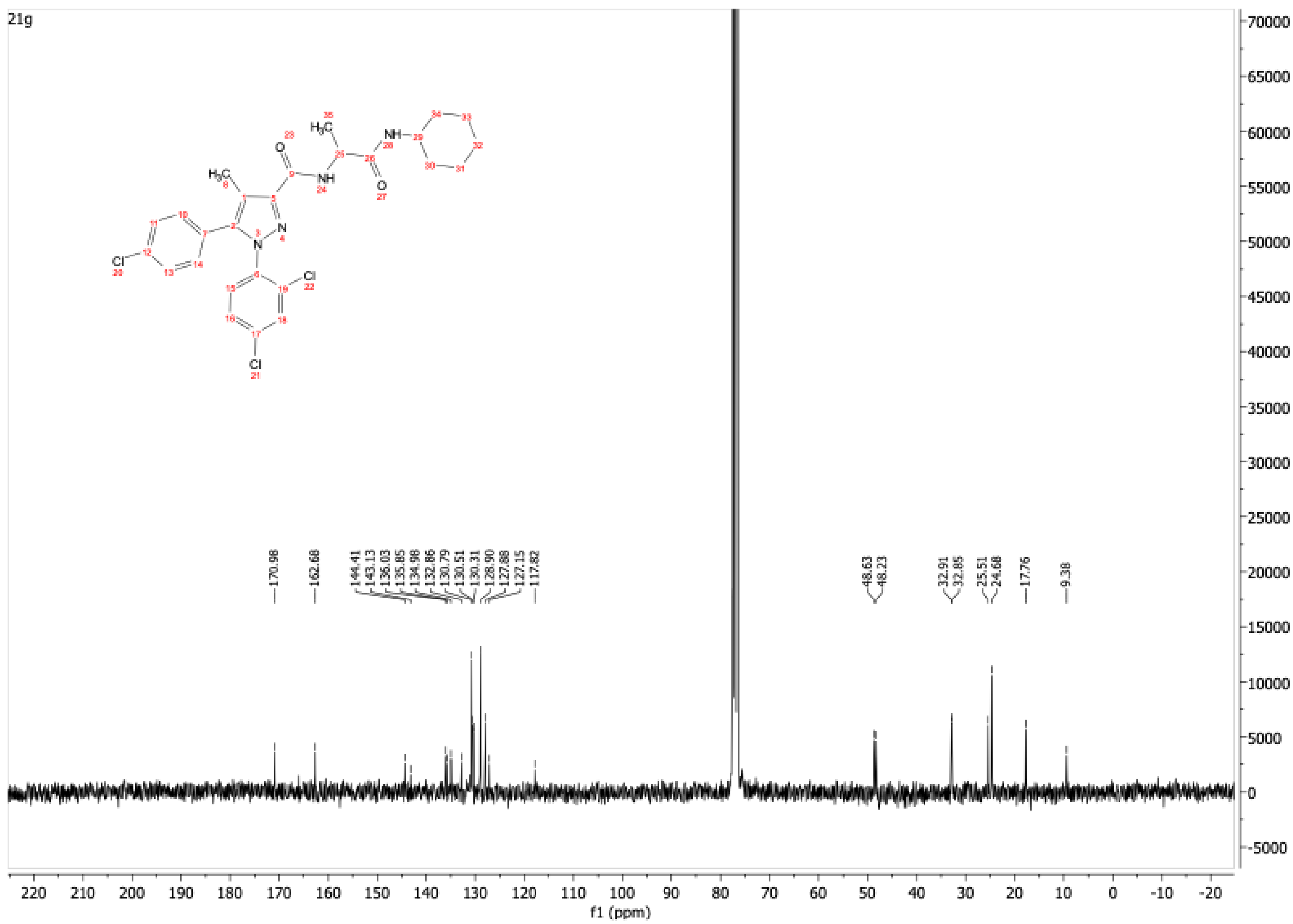
21g
170.98
162.68
144.41
143.13
136.03
135.85
134.98
132.86
130.79
130.51
130.31
128.90
127.88
127.15
117.82
48.63
48.23
32.91
32.85
25.51
24.68
17.76
9.38
f1 (ppm)

674

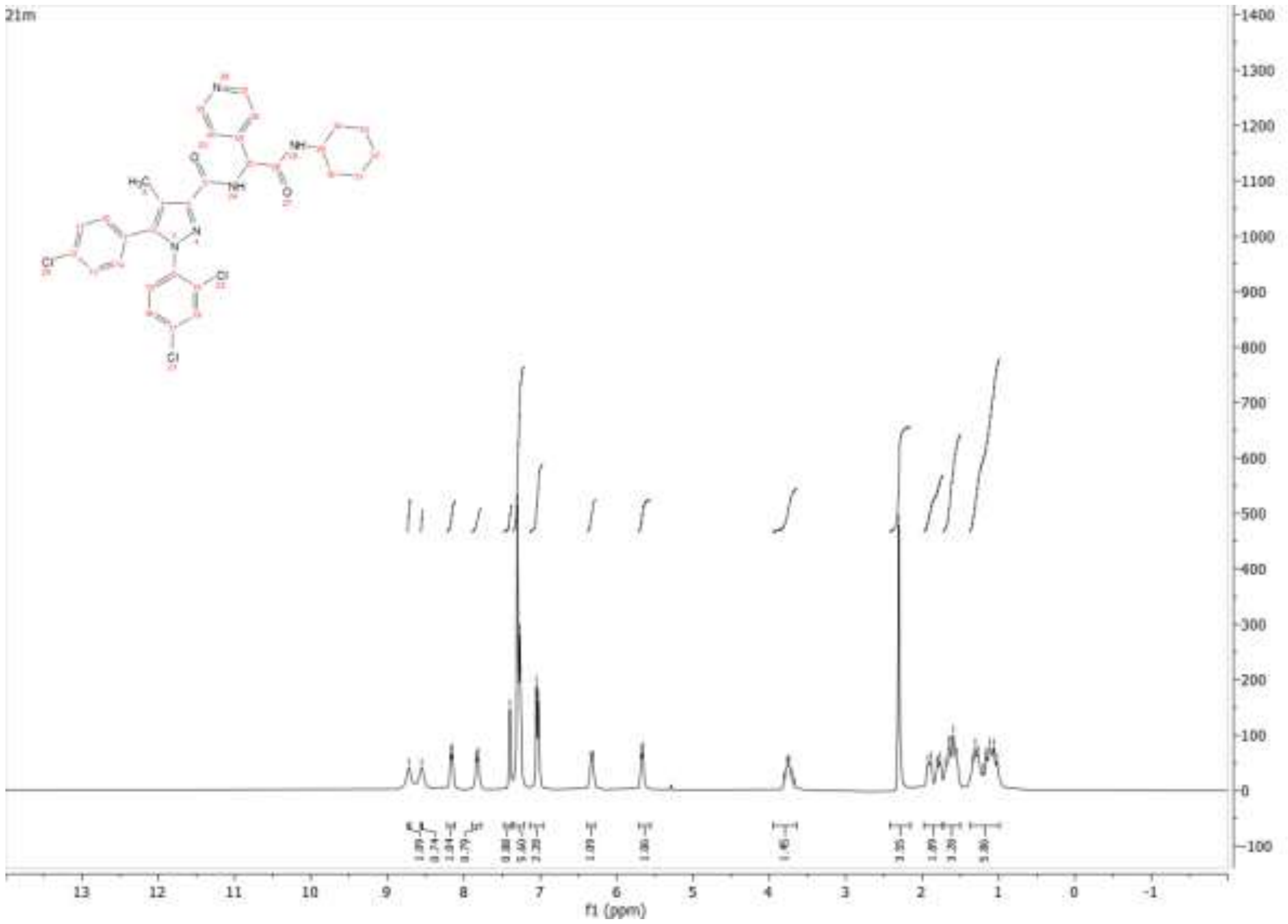
21m
f1 (ppm)

675

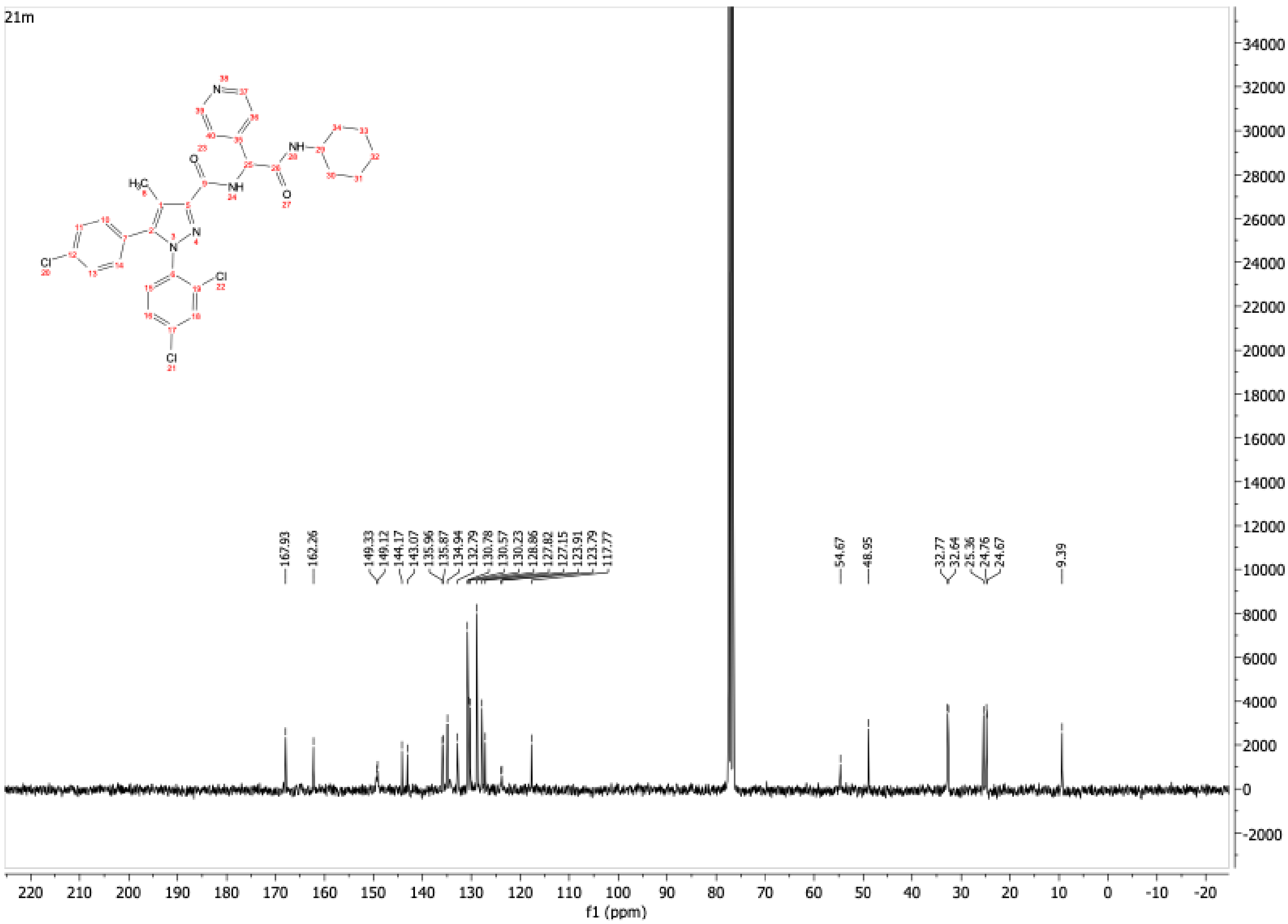
21m
167.93
162.26
149.33
149.12
144.17
143.07
135.96
135.87
134.94
132.79
130.78
130.57
130.23
128.86
127.82
127.15
123.91
123.79
117.77
54.67
48.95
32.77
32.64
25.36
24.76
24.67
9.39
f1 (ppm)

676

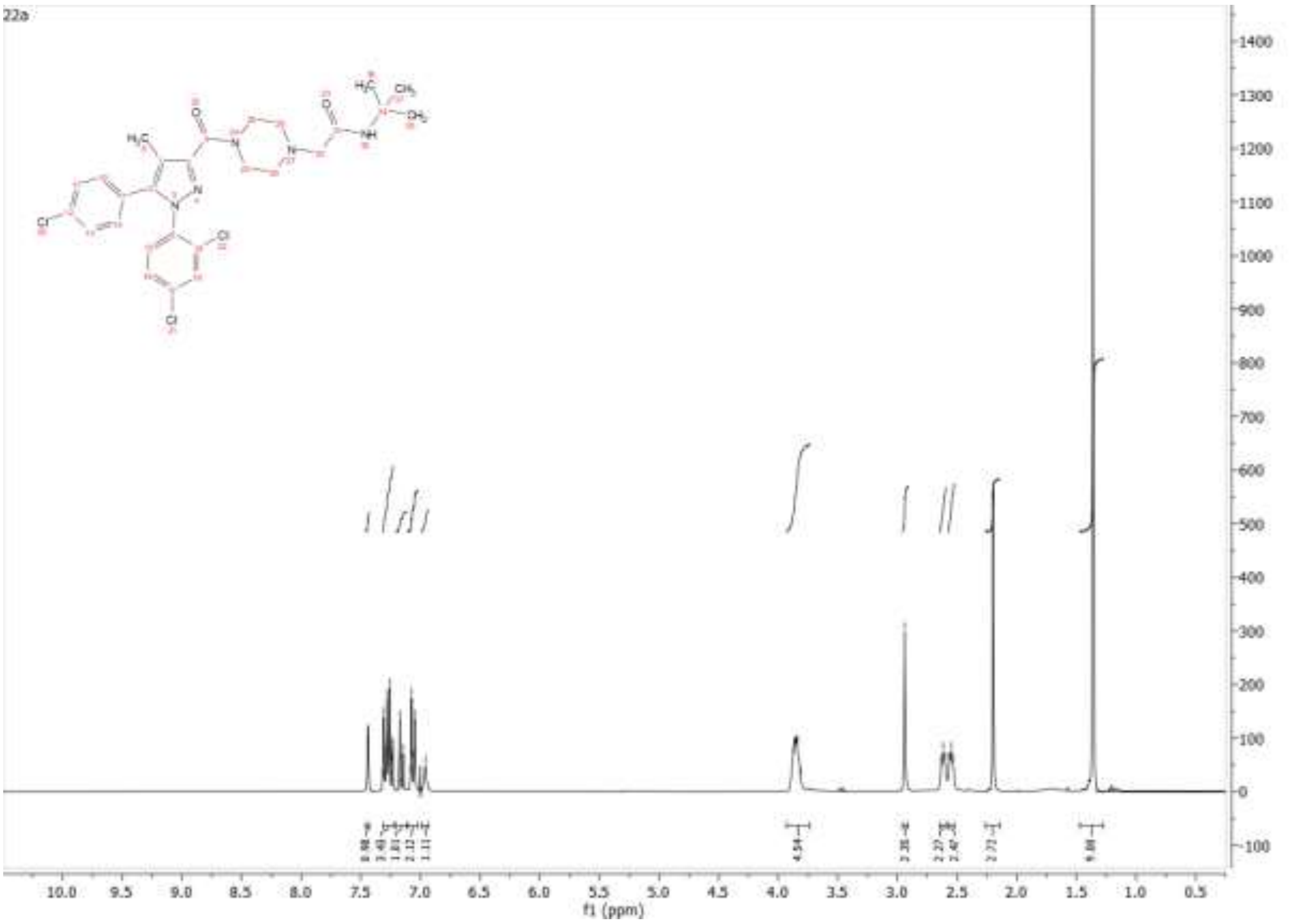
22a
f1 (ppm)

677

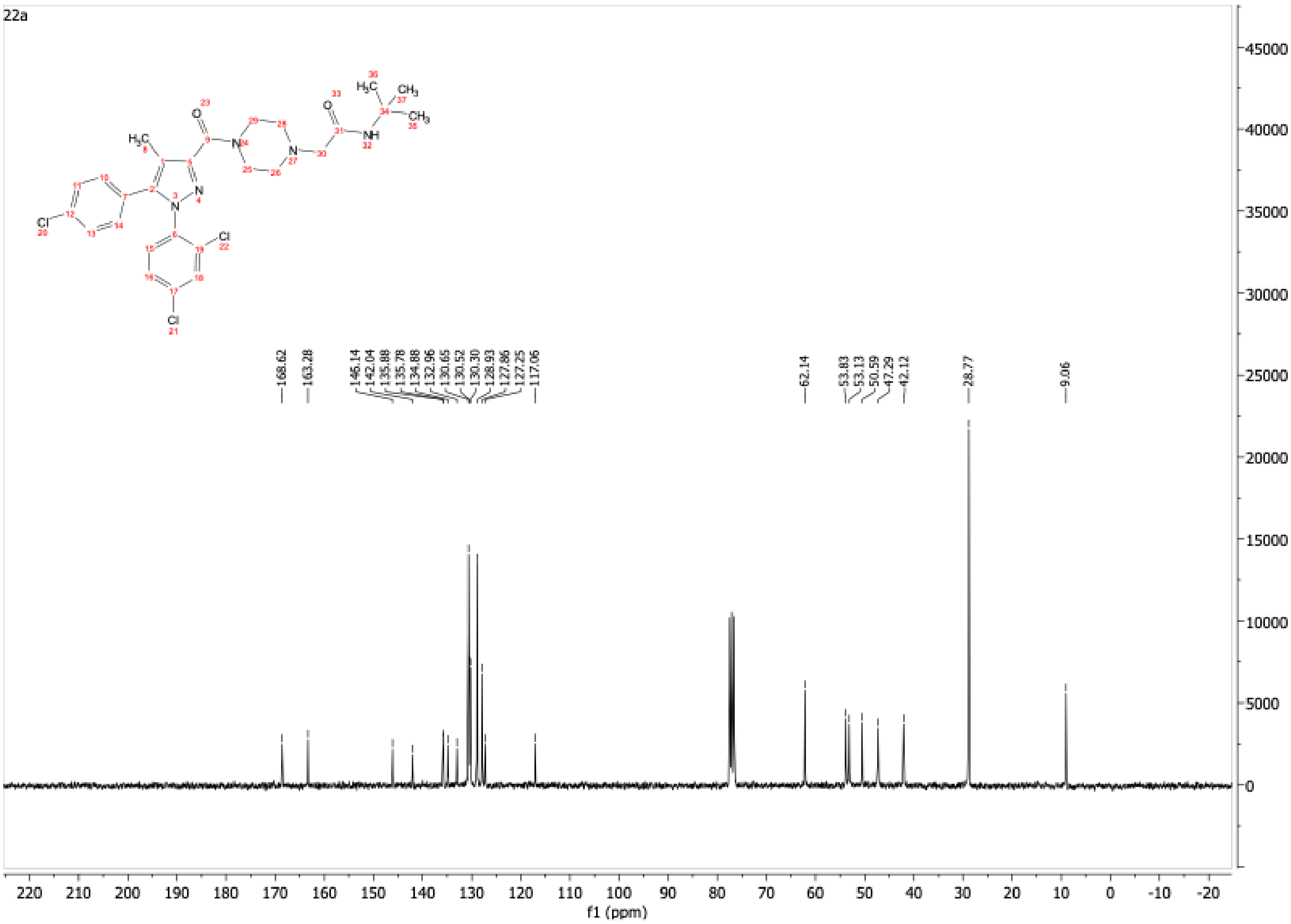
22a
168.62
163.28
146.14
142.04
135.88
135.78
134.88
132.96
130.65
130.52
130.30
128.93
127.86
127.25
117.06
62.14
53.83
53.13
50.59
47.29
42.12
28.77
9.06
f1 (ppm)

678

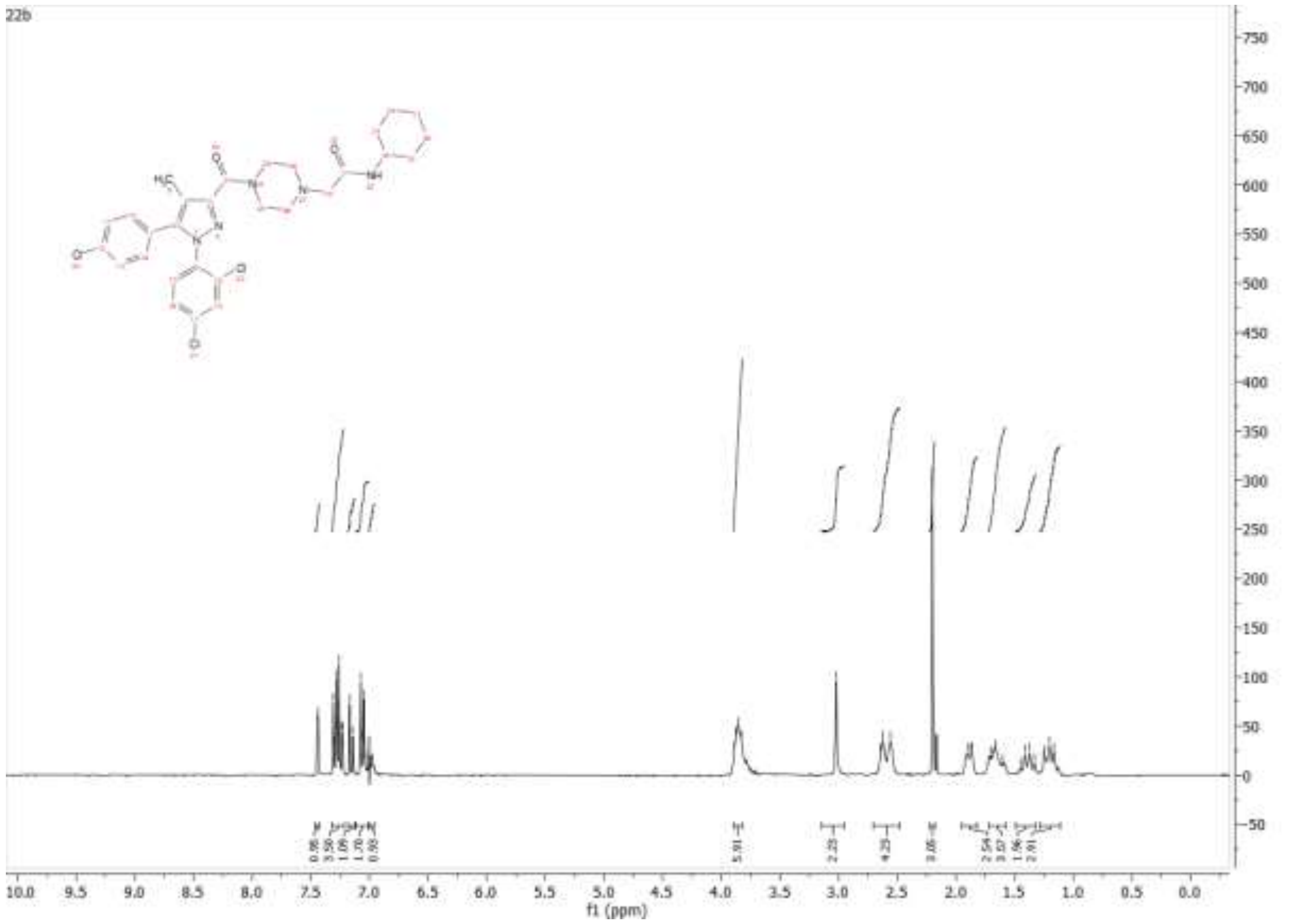
22b
H3C
NH
Cl
f1 (ppm)

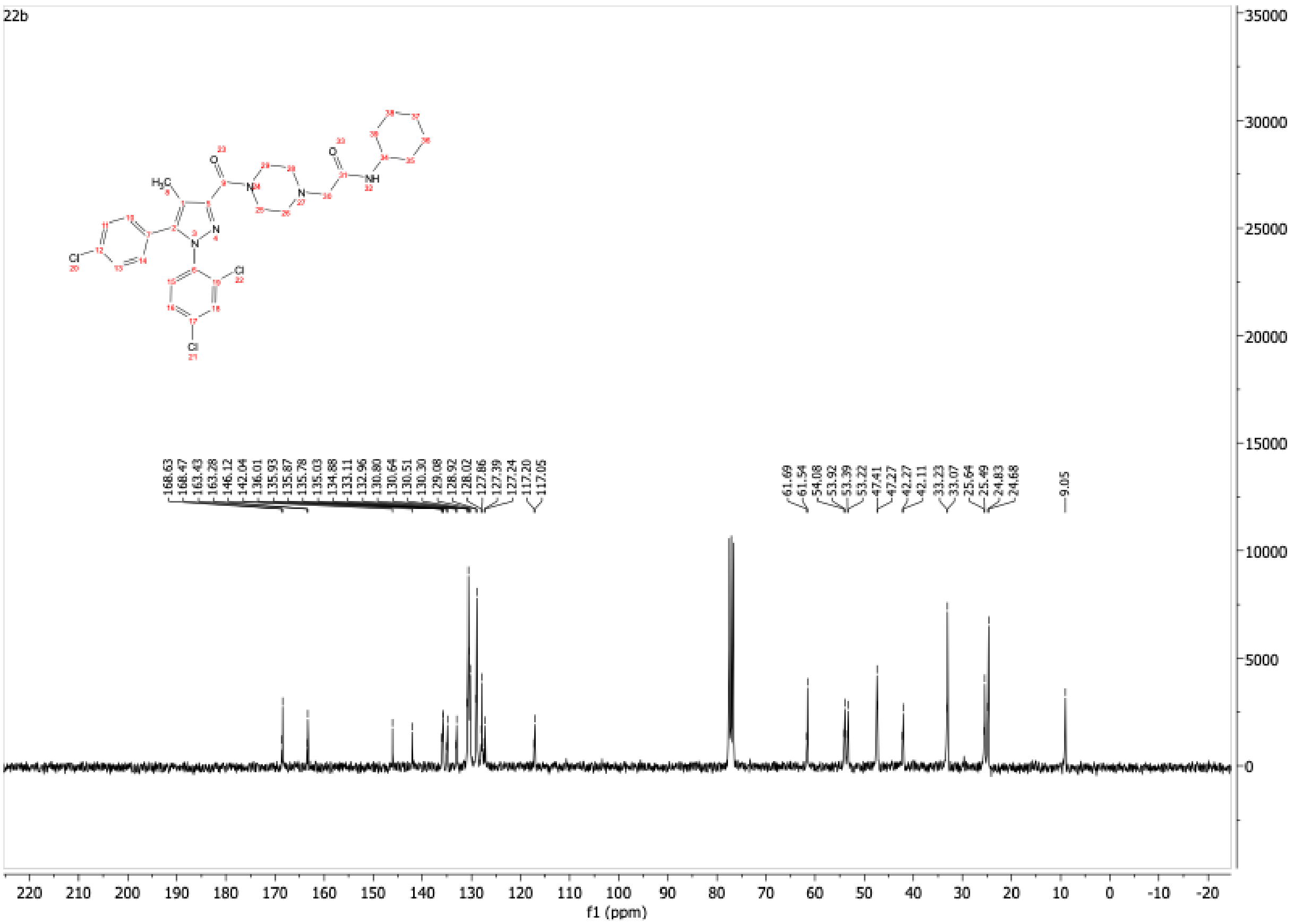
22b
H3C
NH
Cl
168.63
168.47
163.43
163.28
146.12
142.04
136.01
135.93
135.87
135.78
135.03
134.88
133.11
132.96
130.80
130.64
130.51
130.30
129.08
128.92
128.02
127.86
127.39
127.24
117.20
117.05
61.69
61.54
54.08
53.92
53.39
53.22
47.41
47.27
42.27
42.11
33.23
33.07
25.64
25.49
24.83
24.68
9.05
f1 (ppm)

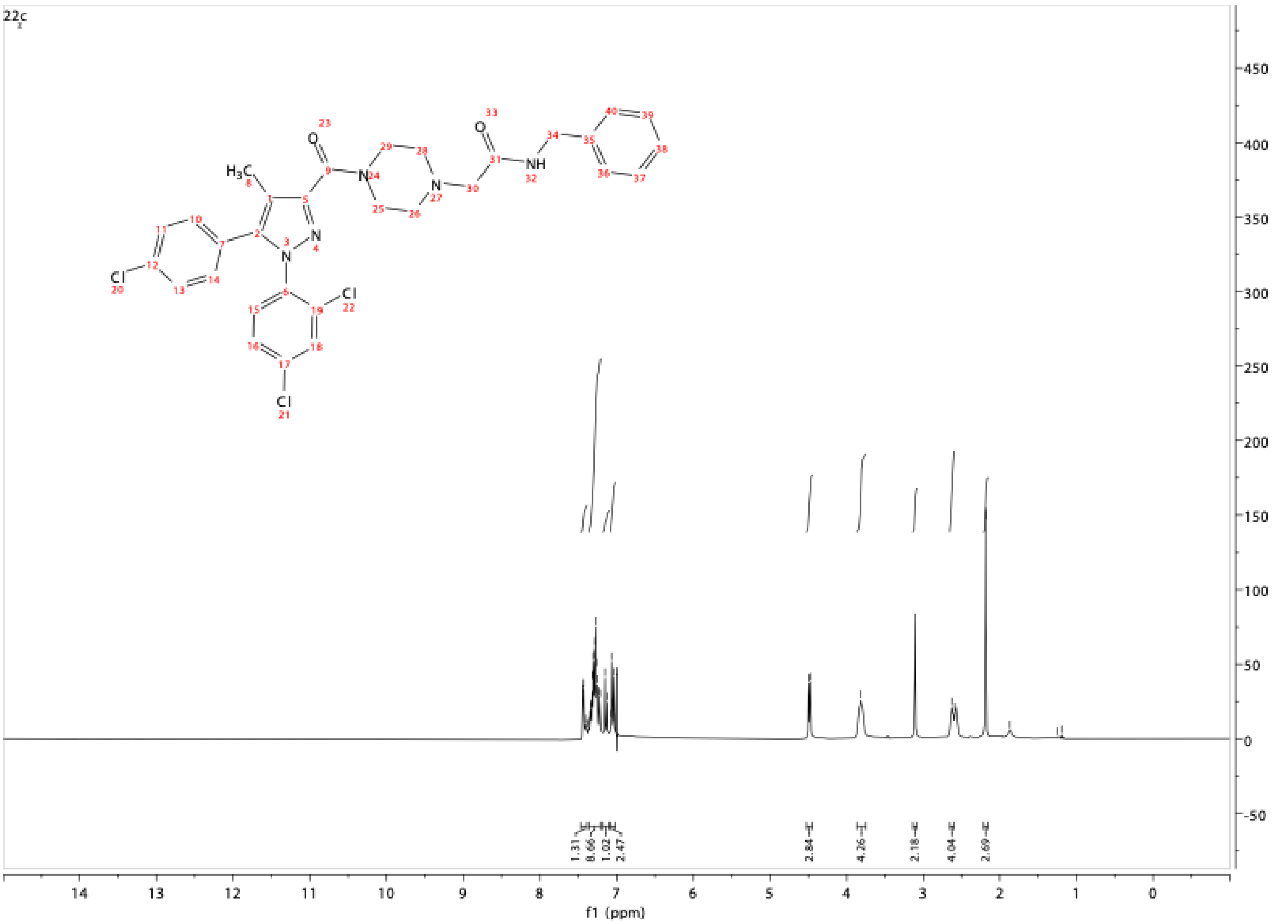
22c
1.31
8.66
1.02
2.47
2.84
4.26
2.18
4.04
2.69
f1 (ppm)

681

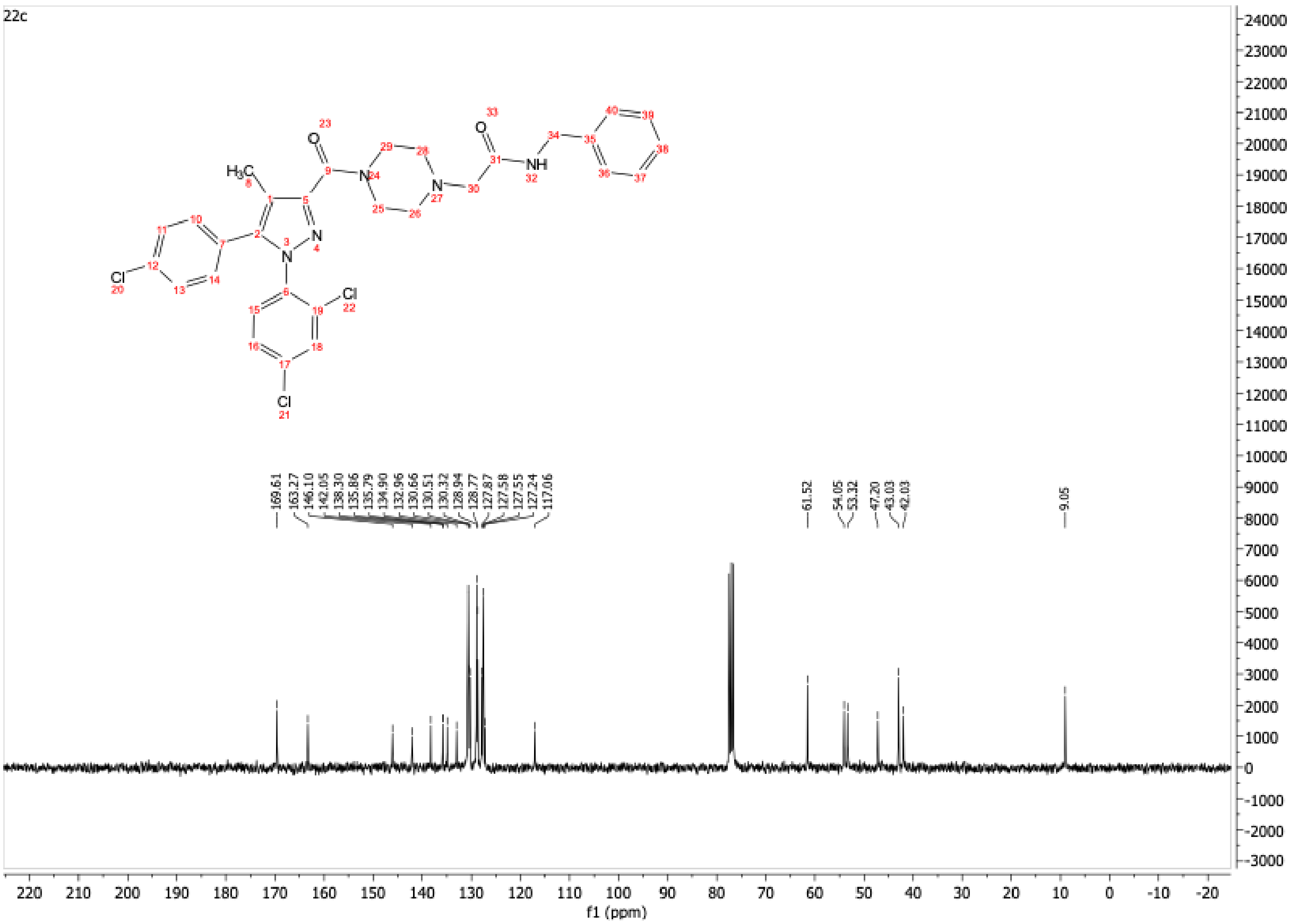
22c
169.61
163.27
146.10
142.05
138.30
135.86
135.79
134.90
132.96
130.66
130.51
130.32
128.94
128.77
127.87
127.58
127.55
127.24
117.06
61.52
54.05
53.32
47.20
43.03
42.03
9.05
f1 (ppm)

682

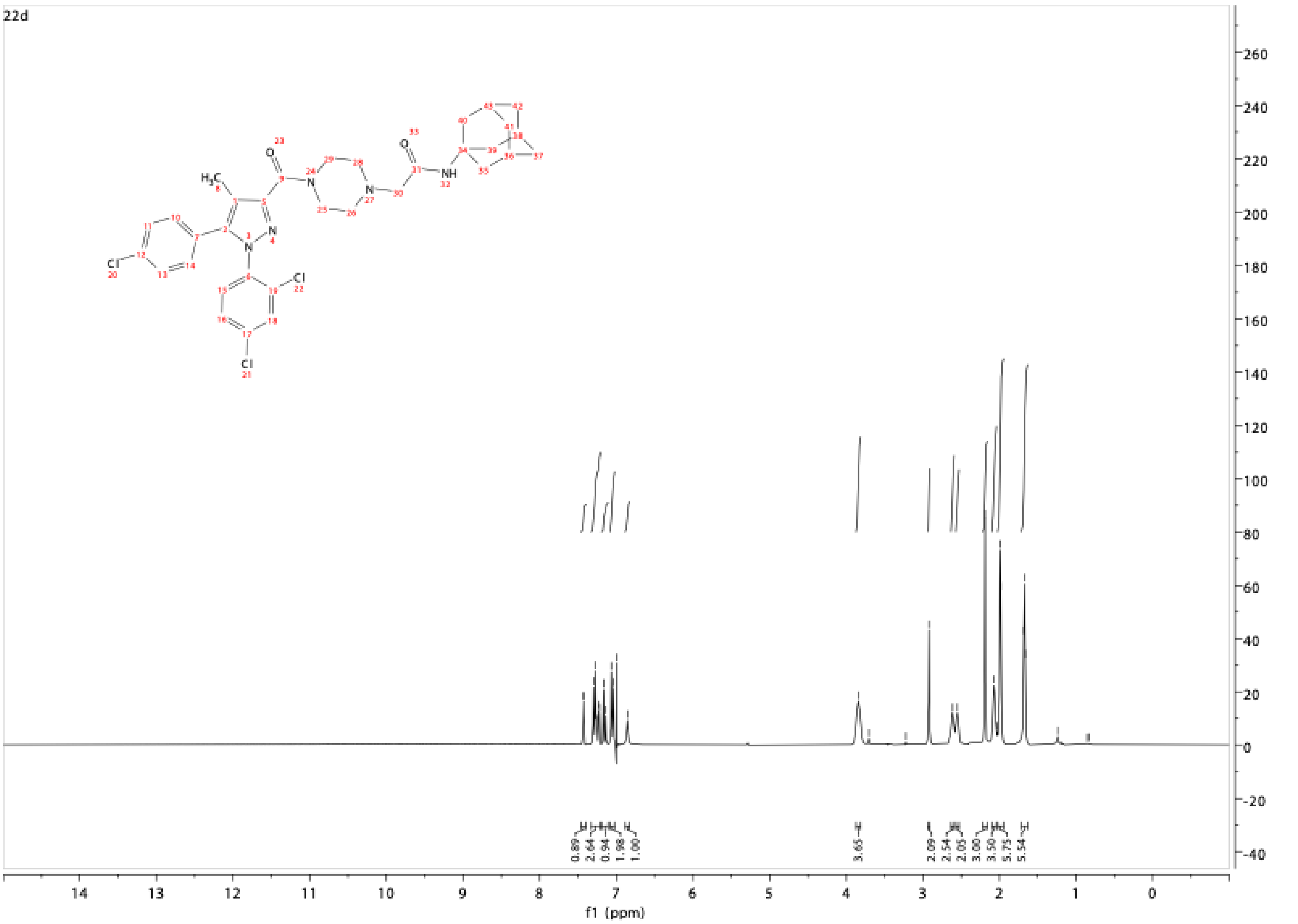

683

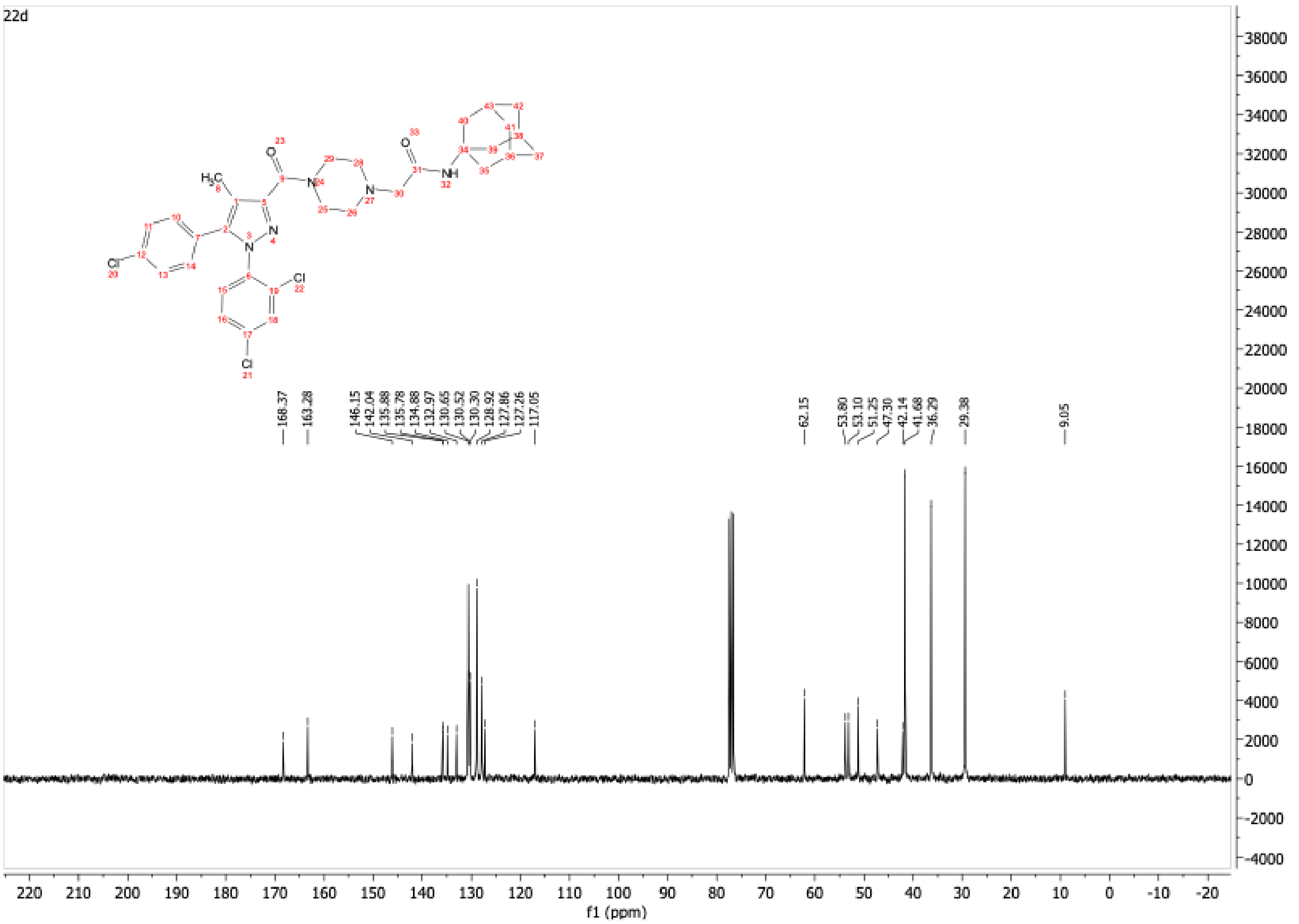

684

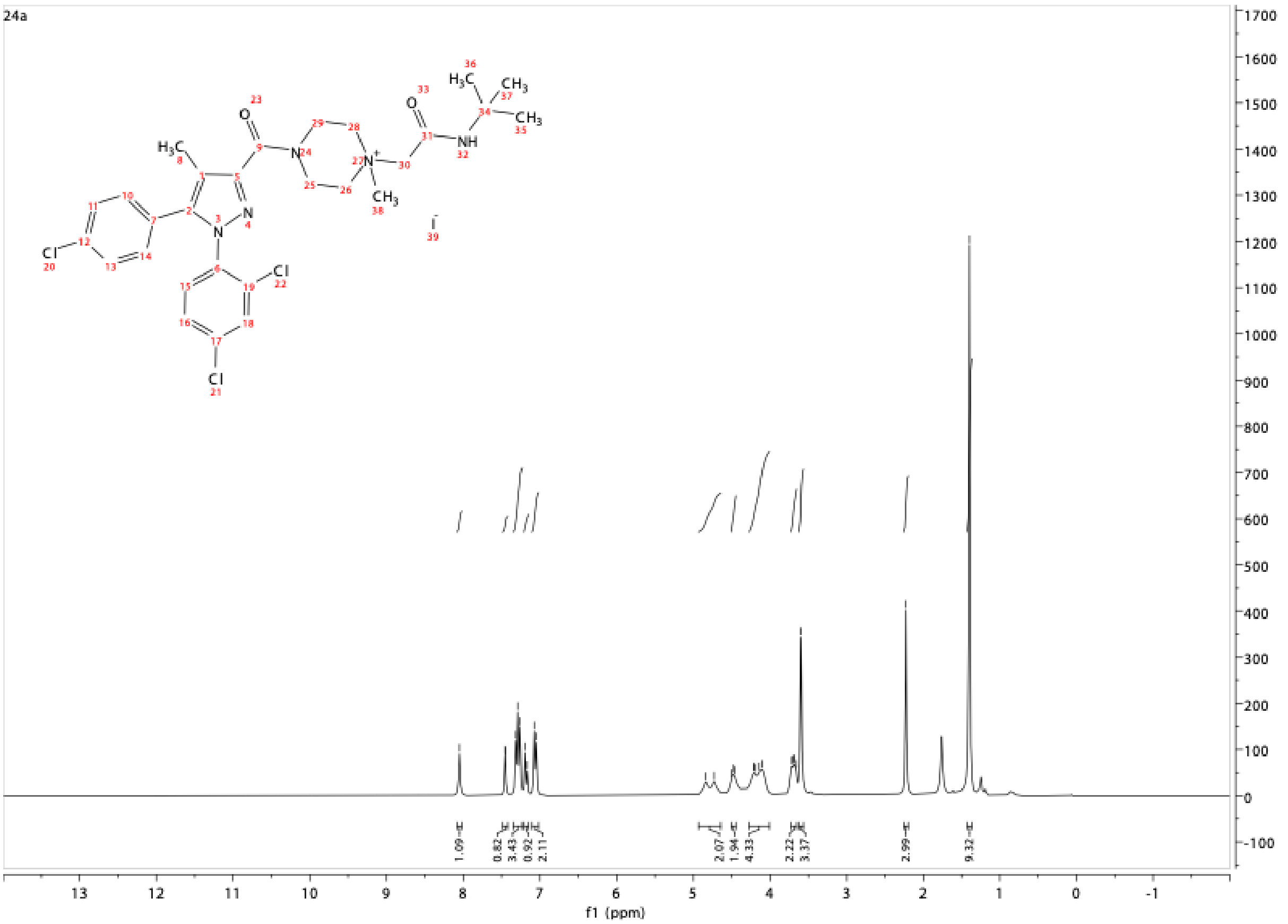
24a
f1 (ppm)

685

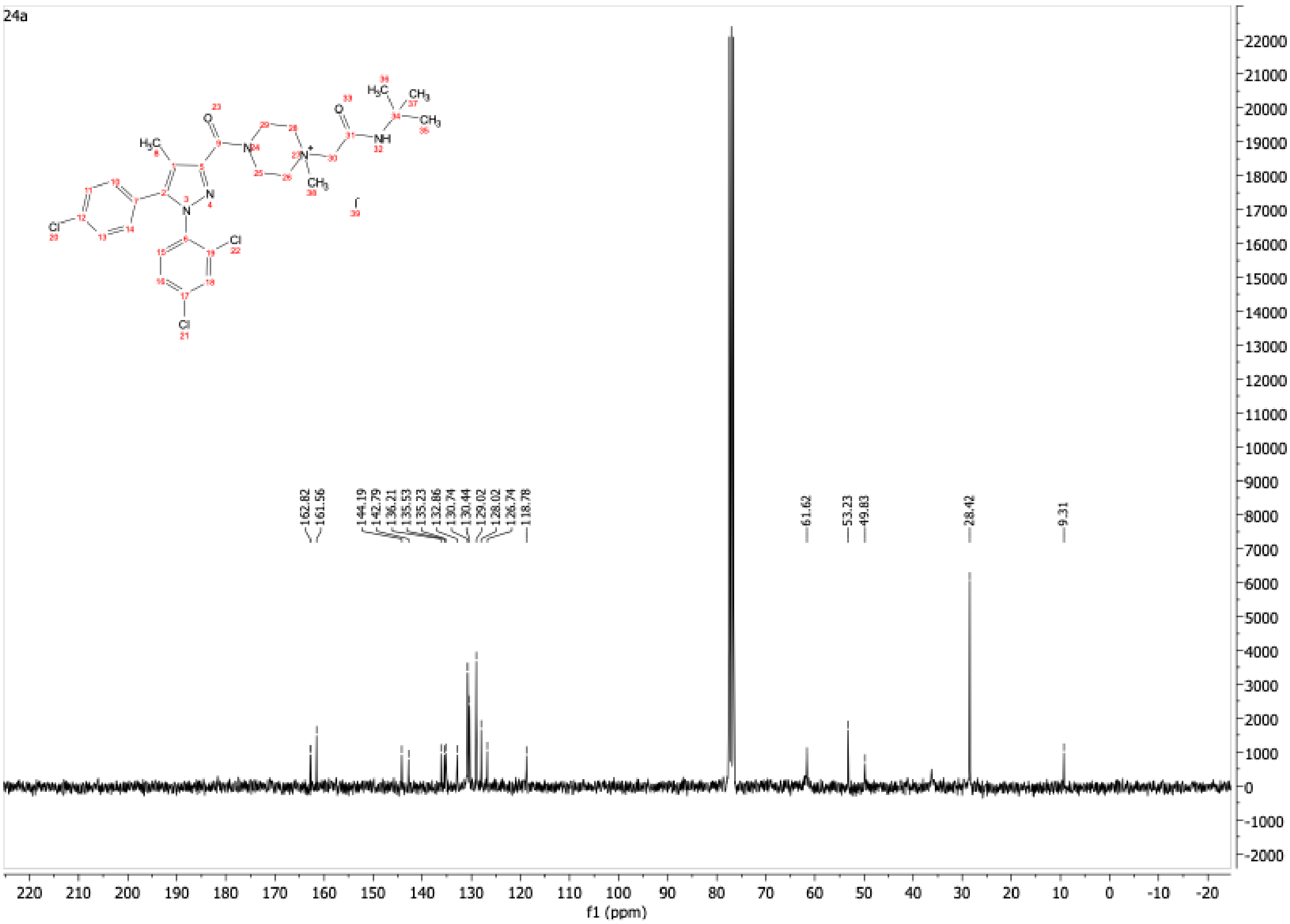
24a
162.82
161.56
144.19
142.79
136.21
135.53
135.23
132.86
130.74
130.44
129.02
128.02
126.74
118.78
61.62
53.23
49.83
28.42
9.31
f1 (ppm)

686

Supplementary Figures

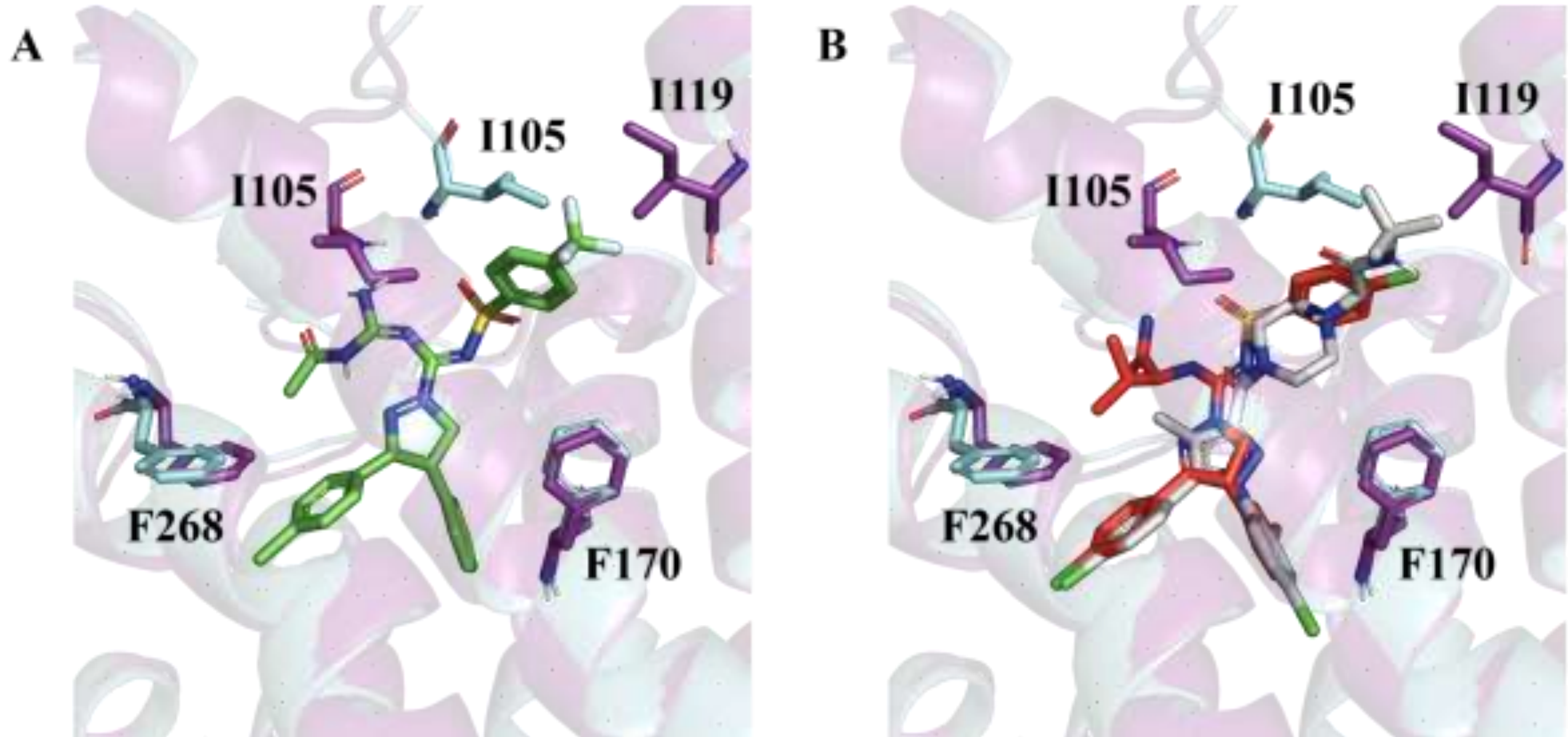

Figure S1. Comparison of MRI-1891 and the predicted binding pose of JD5037 within the CB1R structure 9BA0. (A) Experimentally determined pose of the co-resolved ligand MRI-1891, shown in green. (B) Top-ranked Glide SP pose of JD5037, shown in red. JD5037 occupies the same antagonist-binding cavity and adopts an overall orientation consistent with that of MRI-1891, including a similar trajectory of the bulky N-linked polar substituent toward the extracellular region. Selected binding-site residues are shown as sticks. The receptor is rendered as a cyan cartoon; ligands and selected residues are shown as sticks; only polar hydrogen atoms are displayed. The docking protocol was validated by redocking the co-resolved ligand MRI-1891 into the 9BA0 binding site using Glide SP. The experimental binding mode was reproduced with a heavy-atom RMSD of 0.71 Å. JD5037 was subsequently docked using the same protocol. The top-ranked pose yielded a Glide SP docking score of -10.10 kcal/mol and showed an overall orientation consistent with that of MRI-1891. In particular, the bulky N-linked polar substituent followed a similar trajectory toward the extracellular region of the binding cavity. These results support the structural compatibility of the 9BA0 receptor conformation with the JD5037-like substitution topology.

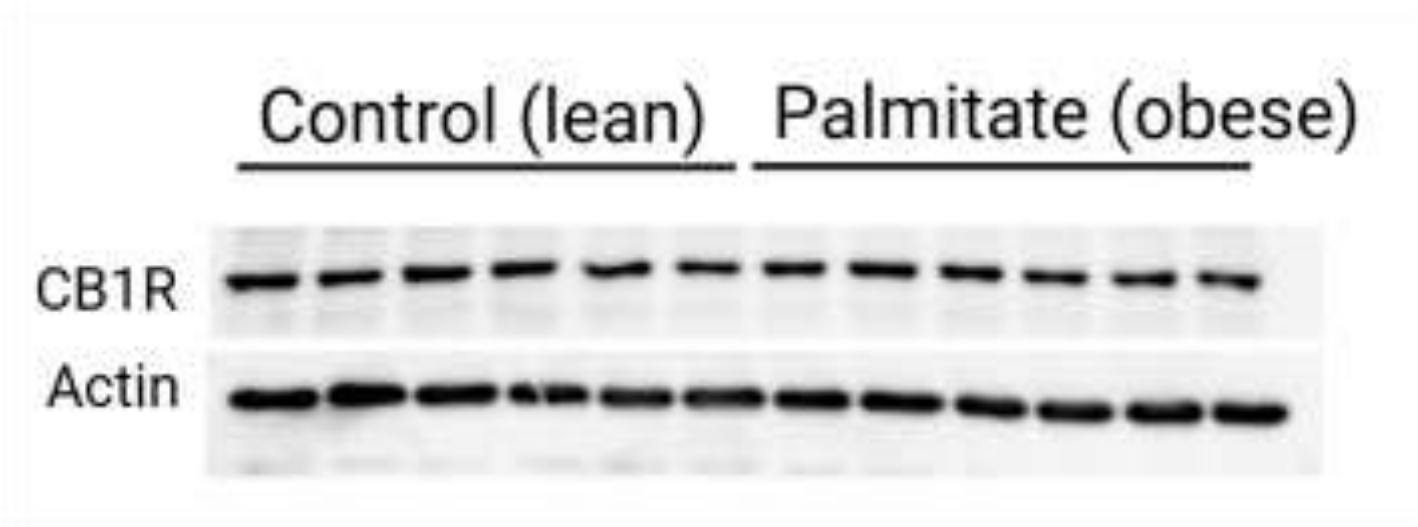

Supplementary Figure S2. Expression of CB_1R in differentiated adipocytes derived from the murine mesenchymal stem cell line C3H10T1/2 under control (lean) and palmitate-treated (obese) conditions. Representative immunoblot showing CB_1R protein levels normalized to actin as loading control.

References

(1) Elders, N.; Ruijter, E.; de Kanter, F. J. J.; Janssen, E.; Lutz, M.; Spek, A. L.; Orru, R. V. A. A Multicomponent Reaction Towards N-(Cyanomethyl)Amides. *Chem. – Eur. J.* **2009**, *15*, 6096–6099.
